# Inborn errors of OAS–RNase L in SARS-CoV-2–related multisystem inflammatory syndrome in children

**DOI:** 10.1126/science.abo3627

**Published:** 2023-02-10

**Authors:** Danyel Lee, Jérémie Le Pen, Ahmad Yatim, Beihua Dong, Yann Aquino, Masato Ogishi, Rémi Pescarmona, Estelle Talouarn, Darawan Rinchai, Peng Zhang, Magali Perret, Zhiyong Liu, Iolanda Jordan, Sefika Elmas Bozdemir, Gulsum Iclal Bayhan, Camille Beaufils, Lucy Bizien, Aurelie Bisiaux, Weite Lei, Milena Hasan, Jie Chen, Christina Gaughan, Abhishek Asthana, Valentina Libri, Joseph M. Luna, Fabrice Jaffré, H.-Heinrich Hoffmann, Eleftherios Michailidis, Marion Moreews, Yoann Seeleuthner, Kaya Bilguvar, Shrikant Mane, Carlos Flores, Yu Zhang, Andrés A. Arias, Rasheed Bailey, Agatha Schlüter, Baptiste Milisavljevic, Benedetta Bigio, Tom Le Voyer, Marie Materna, Adrian Gervais, Marcela Moncada-Velez, Francesca Pala, Tomi Lazarov, Romain Levy, Anna-Lena Neehus, Jérémie Rosain, Jessica Peel, Yi-Hao Chan, Marie-Paule Morin, Rosa Maria Pino-Ramirez, Serkan Belkaya, Lazaro Lorenzo, Jordi Anton, Selket Delafontaine, Julie Toubiana, Fanny Bajolle, Victoria Fumadó, Marta L. DeDiego, Nadhira Fidouh, Flore Rozenberg, Jordi Pérez-Tur, Shuibing Chen, Todd Evans, Frédéric Geissmann, Pierre Lebon, Susan R. Weiss, Damien Bonnet, Xavier Duval, CoV-Contact Cohort, COVID Human Genetic Effort, Qiang Pan-Hammarström, Anna M. Planas, Isabelle Meyts, Filomeen Haerynck, Aurora Pujol, Vanessa Sancho-Shimizu, Clifford L. Dalgard, Jacinta Bustamante, Anne Puel, Stéphanie Boisson-Dupuis, Bertrand Boisson, Tom Maniatis, Qian Zhang, Paul Bastard, Luigi Notarangelo, Vivien Béziat, Rebeca Perez de Diego, Carlos Rodriguez-Gallego, Helen C. Su, Richard P. Lifton, Emmanuelle Jouanguy, Aurélie Cobat, Laia Alsina, Sevgi Keles, Elie Haddad, Laurent Abel, Alexandre Belot, Lluis Quintana-Murci, Charles M. Rice, Robert H. Silverman, Shen-Ying Zhang, Jean-Laurent Casanova

**Affiliations:** 1 St. Giles Laboratory of Human Genetics of Infectious Diseases, Rockefeller Branch, The Rockefeller University, New York, NY, USA.; 2 Laboratory of Human Genetics of Infectious Diseases, Necker Branch, INSERM U1163, Paris, France.; 3 Paris City University, Imagine Institute, Paris, France.; 4 Laboratory of Virology and Infectious Disease, The Rockefeller University, New York, NY, USA.; 5 Department of Cancer Biology, Lerner Research Institute, Cleveland Clinic, Cleveland, OH, USA.; 6 Human Evolutionary Genetics Unit, Institut Pasteur, Paris City University, CNRS UMR 2000, Paris, France.; 7 Doctoral College, Sorbonne University, Paris, France.; 8 Laboratory of Immunology, Lyon Sud Hospital, Lyon, France.; 9 Pediatric Intensive Care Department, Hospital Sant Joan de Déu, Barcelona, Spain.; 10 Kids Corona Platform, Barcelona, Spain.; 11 Center for Biomedical Network Research on Epidemiology and Public Health (CIBERESP), Instituto de Salud Carlos III, Madrid, Spain.; 12 Department of Surgery and Surgical Specializations, Faculty of Medicine and Health Sciences, University of Barcelona, Barcelona, Spain.; 13 Respiratory and Immunological Dysfunction in Pediatric Critically Ill Patients, Institute of Recerca Sant Joan de Déu, Barcelona, Spain.; 14 Bursa City Hospital, Bursa, Turkey.; 15 Ankara City Hospital, Yildirim Beyazit University, Ankara, Turkey.; 16 Immunology and Rheumatology Division, Department of Pediatrics, University of Montreal, CHU Sainte-Justine, Montreal, QC, Canada.; 17 Center for Translational Research, Institut Pasteur, Paris City University, Paris, France.; 18 Department of Biochemistry and Center for RNA Science and Therapeutics, Case Western Reserve University, Cleveland, OH, USA.; 19 Department of Surgery, Weill Cornell Medical College, New York, NY, USA.; 20 Department of Pediatrics, School of Medicine, Emory University, Atlanta, GA, USA.; 21 International Center of Infectiology Research (CIRI), University of Lyon, INSERM U1111, Claude Bernard University, Lyon 1, CNRS, UMR5308, ENS of Lyon, Lyon, France.; 22 Departments of Neurosurgery and Genetics and Yale Center for Genome Analysis, Yale School of Medicine, New Haven, CT, USA.; 23 Department of Medical Genetics, School of Medicine, Acibadem Mehmet Ali Aydinlar University, Istanbul, Turkey.; 24 Department of Genetics, Yale University School of Medicine, New Haven, CT, USA.; 25 Research Unit, Nuestra Señora de la Candelaria University Hospital, Santa Cruz de Tenerife, Spain.; 26 Genomics Division, Institute of Technology and Renewable Energies (ITER), Granadilla de Abona, Spain.; 27 CIBERES, ISCIII, Madrid, Spain.; 28 Department of Clinical Sciences, University Fernando Pessoa Canarias, Las Palmas de Gran Canaria, Spain.; 29 Laboratory of Clinical Immunology and Microbiology, Division of Intramural Research, NIAID, NIH, Bethesda, MD, USA.; 30 NIAID Clinical Genomics Program, NIH, Laboratory of Clinical Immunology and Microbiology, Division of Intramural Research, NIAID, NIH, Bethesda, MD, USA.; 31 Primary Immunodeficiencies Group, University of Antioquia (UdeA), Medellin, Colombia.; 32 School of Microbiology, University of Antioquia (UdeA), Medellin, Colombia.; 33 Neurometabolic Diseases Laboratory, IDIBELL–Hospital Duran I Reynals, CIBERER U759, ISIiii, Madrid, Spain.; 34 Immunology Program, Memorial Sloan Kettering Cancer Center, New York, NY, USA.; 35 Pediatrics Department, Hospital Sant Joan de Déu, Barcelona, Spain.; 36 Department of Molecular Biology and Genetics, Bilkent University, Ankara, Turkey.; 37 Pediatric Rheumatology Division, Hospital Sant Joan de Déu, Barcelona, Spain.; 38 Study Group for Immune Dysfunction Diseases in Children (GEMDIP), Institute of Recerca Sant Joan de Déu, Barcelona, Spain.; 39 Department of Pediatrics, University Hospitals Leuven, Leuven, Belgium.; 40 Department of General Pediatrics and Pediatric Infectious Diseases, Necker Hospital for Sick Children, Assistance Publique–Hôpitaux de Paris (AP-HP), Paris City University, Paris, France.; 41 Biodiversity and Epidemiology of Bacterial Pathogens, Pasteur Institute, Paris, France.; 42 Department of Pediatric Cardiology, Necker Hospital for Sick Children, AP-HP, Paris City University, Paris, France.; 43 Pediatrics Infectious Diseases Division, Hospital Sant Joan de Déu, Barcelona, Spain.; 44 Infectious Diseases and Microbiome, Institute of Recerca Sant Joan de Déu, Barcelona, Spain.; 45 Department of Molecular and Cellular Biology, National Center for Biotechnology (CNB-CSIC), Madrid, Spain.; 46 Laboratory of Virology, Bichat–Claude Bernard Hospital, Paris, France.; 47 Laboratory of Virology, AP-HP, Cochin Hospital, Paris, France.; 48 Molecular Genetics Unit, Institute of Biomedicine of Valencia (IBV-CSIC), Valencia, Spain.; 49 CIBERNED, ISCIII, Madrid, Spain.; 50 Joint Research Unit in Neurology and Molecular Genetics, Institut of Investigation Sanitaria La Fe, Valencia, Spain.; 51 Medical School, Paris City University, Paris, France.; 52 Department of Microbiology, Perelman School of Medicine, University of Pennsylvania, Philadelphia, PA, USA.; 53 Bichat–Claude Bernard Hospital, Paris, France.; 54 University Paris Diderot, Paris 7, UFR of Médecine-Bichat, Paris, France.; 55 IAME, INSERM, UMRS1137, Paris City University, Paris, France.; 56 Infectious and Tropical Diseases Department, AP-HP, Bichat–Claude Bernard Hospital, Paris, France.; 57 Department of Biosciences and Nutrition, Karolinska Institutet, Huddinge, Sweden.; 58 Department of Neuroscience and Experimental Therapeutics, Institute for Biomedical Research of Barcelona (IIBB), Spanish National Research Council (CSIC), Barcelona, Spain.; 59 Institute for Biomedical Investigations August Pi i Sunyer (IDIBAPS), Barcelona, Spain.; 60 Department of Pediatrics, University Hospitals Leuven and Laboratory for Inborn Errors of Immunity, KU Leuven, Leuven, Belgium.; 61 Primary Immunodeficiency Research Laboratory, Center for Primary Immunodeficiency Ghent, Ghent University Hospital, Ghent, Belgium.; 62 Neurometabolic Diseases Laboratory, IDIBELL–Hospital Duran I Reynals; and Catalan Institution for Research and Advanced Studies (ICREA), Barcelona, Spain.; 63 CIBERER U759, ISCiii, Madrid, Spain.; 64 Department of Paediatric Infectious Diseases and Virology, Imperial College London, London, UK.; 65 Centre for Paediatrics and Child Health, Faculty of Medicine, Imperial College London, London, UK.; 66 The American Genome Center, Collaborative Health Initiative Research Program, Uniformed Services University of the Health Sciences, Bethesda, MD, USA.; 67 Department of Anatomy, Physiology, and Genetics, Uniformed Services University of the Health Sciences, Bethesda, MD, USA.; 68 Study Center for Primary Immunodeficiencies, Necker Hospital for Sick Children, AP-HP, Paris, France.; 69 New York Genome Center, New York, NY, USA.; 70 Pediatric Hematology-Immunology and Rheumatology Unit, Necker Hospital for Sick Children, AP-HP, Paris, France.; 71 Laboratory of Immunogenetics of Human Diseases, Innate Immunity Group, IdiPAZ Institute for Health Research, La Paz Hospital, Madrid, Spain.; 72 Interdepartmental Group of Immunodeficiencies, Madrid, Spain.; 73 Department of Immunology, University Hospital of Gran Canaria Dr. Negrín, Canarian Health System, Las Palmas de Gran Canaria, Spain.; 74 Laboratory of Human Genetics and Genomics, The Rockefeller University, New York, NY, USA.; 75 Clinical Immunology and Primary Immunodeficiencies Unit, Pediatric Allergy and Clinical Immunology Department, Hospital Sant Joan de Déu, Barcelona, Spain.; 76 Necmettin Erbakan University, Konya, Turkey.; 77 Department of Pediatrics, Department of Microbiology, Immunology and Infectious Diseases, University of Montreal and Immunology and Rheumatology Division, CHU Sainte-Justine, Montreal, QC, Canada.; 78 National Reference Center for Rheumatic, Autoimmune and Systemic Diseases in Children (RAISE), Pediatric Nephrology, Rheumatology, Dermatology Unit, Hospital of Mother and Child, Hospices Civils of Lyon, Lyon, France.; 79 Human Genomics and Evolution, Collège de France, Paris, France.; 80 Department of Pediatrics, Necker Hospital for Sick Children, Paris, France.; 81 Howard Hughes Medical Institute, The Rockefeller University, New York, NY, USA.

## Abstract

Multisystem inflammatory syndrome in children (MIS-C) is a rare and severe condition that follows benign COVID-19. We report autosomal recessive deficiencies of *OAS1*, *OAS2,* or RNASEL in five unrelated children with MIS-C. The cytosolic double-stranded RNA (dsRNA)–sensing OAS1 and OAS2 generate 2′-5′-linked oligoadenylates (2–5A) that activate the single-stranded RNA–degrading ribonuclease L (RNase L). Monocytic cell lines and primary myeloid cells with OAS1, OAS2, or RNase L deficiencies produce excessive amounts of inflammatory cytokines upon dsRNA or severe acute respiratory syndrome coronavirus 2 (SARS-CoV-2) stimulation. Exogenous 2–5A suppresses cytokine production in OAS1-deficient but not RNase L–deficient cells. Cytokine production in RNase L–deficient cells is impaired by MDA5 or RIG-I deficiency and abolished by mitochondrial antiviral-signaling protein (MAVS) deficiency. Recessive OAS–RNase L deficiencies in these patients unleash the production of SARS-CoV-2–triggered, MAVS-mediated inflammatory cytokines by mononuclear phagocytes, thereby underlying MIS-C.

## Abstract (summary)

**INTRODUCTION:**

Multisystem inflammatory syndrome in children (MIS-C) is a severe, unexplained complication of severe acute respiratory syndrome coronavirus 2 (SARS-CoV-2) infection with an estimated prevalence of ~1 per 10,000 infected children. It typically occurs 4 weeks after infection, without hypoxemic pneumonia. Affected children present with fever, rash, abdominal pain, myocarditis, and other clinical features reminiscent of Kawasaki disease, including lymphadenopathy, coronary aneurysm, and high levels of biological markers of acute inflammation. Sustained monocyte activation is consistently reported as a key immunological feature of MIS-C. A more specific immunological abnormality is the polyclonal expansion of CD4^+^ and CD8^+^ T cells bearing the T cell receptor Vβ21.3. The root cause of MIS-C and its immunological and clinical features remains unknown.

**RATIONALE:**

We hypothesized that monogenic inborn errors of immunity to SARS-CoV-2 may underlie MIS-C in some children. We further hypothesized that the identification of these inborn errors would provide insights into the molecular and cellular mechanisms underlying its immunological and clinical phenotypes. Finally, we hypothesized that a genetic and mechanistic understanding of a few patients would provide a proof of principle that would facilitate studies in other patients. We performed whole-exome or whole-genome sequencing on 558 internationally recruited patients with MIS-C (aged 3 months to 19 years). We searched for rare nonsynonymous biallelic variants of protein-coding genes, testing a hypothesis of genetic homogeneity

**RESULTS:**

We found autosomal recessive deficiencies of OAS1 (2′-5′-oligoadenylate synthetase 1), OAS2, or RNase L (ribonuclease L) in five unrelated children of four different ancestries with MIS-C (~1% of our cohort). There were no similar defects in a cohort of 1288 individuals (aged 6 months to 99 years) with asymptomatic or mild infection (*P* = 0.001) or 334 young patients (aged 0 to 21 years) with asymptomatic or mild infection or COVID-19 pneumonia (*P* = 0.046). The estimated cumulative frequency of these defects in the general population was ~0.00013. The type I interferon (IFN)–inducible double-stranded RNA (dsRNA)–sensing proteins OAS1 and OAS2 generate 2′-5′-linked oligoadenylates (2–5A), which activate the antiviral single-stranded RNA (ssRNA)–degrading RNase L, particularly in mononuclear phagocytes. Consistent with the absence of pneumonia in these patients, epithelial cells and fibroblasts defective for this pathway restricted SARS-CoV-2 normally. This contrasted with interferon alpha and beta receptor subunit 1 (IFNAR1)–deficient cells from patients prone to hypoxemic pneumonia without MIS-C. Monocytic cell lines with genetic deficiencies of OAS1, OAS2, or RNase L displayed excessive inflammatory cytokine production in response to intracellular dsRNA. Cytokine production by RNase L–deficient cells was impaired by melanoma differentiation-associated protein 5 (MDA5) or retinoic acid–inducible gene I (RIG-I) deficiency and abolished by mitochondrial antiviral-signaling protein (MAVS) deficiency. Exogenous 2–5A suppressed inflammatory responses to these stimuli in control and OAS1-deficient cells but not in RNase L–deficient cells. Finally, monocytic cell lines, primary monocytes, and monocyte-derived dendritic cells with genetic deficiencies of OAS1, OAS2, or RNase L displayed exaggerated inflammatory responses to SARS-CoV-2 as well as SARS-CoV-2–infected cells and their RNA.

**CONCLUSION:**

We report autosomal recessive deficiencies of OAS1, OAS2, or RNase L in ~1% of an international cohort of MIS-C patients. The cytosolic OAS–RNase L pathway suppresses RIG-I/MDA5–MAVS–mediated inflammation in dsRNA-stimulated mononuclear phagocytes. Single-gene recessive inborn errors of the OAS–RNase L pathway unleash the production of SARS-CoV-2–triggered inflammatory cytokines by mononuclear phagocytes, thereby underlying MIS-C.

## Graphical Abstract

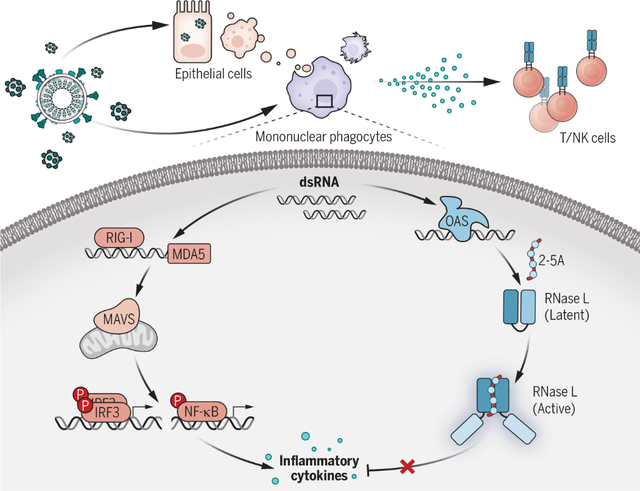

**OAS-RNase L deficiency in MIS-C.** dsRNAs from SARS-CoV-2 or SARS-CoV-2-permissive cells engulfed by mononuclear phagocytes simultaneously activate the RIG-I/MDA5-MAVS pathway, inducing inflammatory cytokine production, and the OAS-RNase L pathway, exerting posttranscriptional control over inflammatory cytokine production. OAS-RNase L deficiency results in excessive inflammatory cytokine production by myeloid cells, triggering MIS-C, including lymphoid cell activation and multiple tissue lesions. NK, natural killer; IRF3, interferon regulatory factor 3; NF-κB, nuclear factor κB.

Interindividual clinical variability in the course of primary infection with severe acute respiratory syndrome coronavirus 2 (SARS-CoV-2) is immense in unvaccinated individuals (1–4). We have shown that inborn errors of type I interferon (IFN) immunity and their phenocopies—autoantibodies against type I IFNs—collectively underlie at least 15% of cases of critical COVID-19 pneumonia in unvaccinated patients (5–9). Common genetic variants act as more modest risk factors (10–13). Children were initially thought to be rarely affected by COVID-19, as only 0.001 to 0.005% of infected children had critical pneumonia (2). However, another severe SARS-CoV-2–related phenotype, multisystem inflammatory syndrome in children (MIS-C), occurs predominantly in children, typically 4 weeks after infection (14–16). Its prevalence is estimated at ~1 per 10,000 infected children (17–19). Children with MIS-C do not suffer from hypoxemic pneumonia and typically display no detectable viral infection of the upper respiratory tract at disease onset. However, most MIS-C cases test positive for anti–SARS-CoV-2 antibodies, and almost all cases have a history of exposure to SARS-CoV-2 (17, 20). Initial reports of MIS-C described it as an atypical form of Kawasaki disease (KD) (16, 21–25), as its clinical features include fever, rash, abdominal pain, myocarditis, lymphadenopathy, coronary aneurysm, and elevated biological markers of acute inflammation.

The elevated markers frequently detected in MIS-C patients suggest that inflammation occurs in various organs (21, 22, 26–36). These markers include surrogates of cardiovascular endothelial injury [e.g., troponin and B-type natriuretic peptide (BNP)] and gastrointestinal epithelial injury [e.g., lipopolysaccharide (LPS)–binding protein (LBP) and soluble CD14] (36). Various leukocyte subsets are also affected. Sustained monocyte activation has been consistently reported as a key immunological feature of MIS-C, with high levels of proinflammatory markers, including ferritin, interleukin-1 receptor antagonist (IL-1RA), IL-6, IL-10, IL-18, monocyte chemoattractant protein 1 (MCP1, or CCL2), and tumor necrosis factor (TNF) (21, 22, 26–36). In addition, the levels of biomarkers related to type II IFN (IFN-γ) signaling, which are not necessarily specific to monocyte activation, often increase during the early phase of disease (22, 31–36). An immunological phenotype specific to MIS-C, observed in ~75% of patients, is the polyclonal expansion of CD4^+^ and CD8^+^ T cells bearing the Vβ21.3 segment (32, 34, 36–38). In this multitude of molecular, cellular, and clinical abnormalities, the root cause of MIS-C remains unknown (39). We hypothesized that monogenic inborn errors of immunity (IEIs) to SARS-CoV-2 may underlie MIS-C in some children and that the identification of these inborn errors may clarify the molecular, cellular, and immunological basis of disease (15, 40).

## Results

### Identification of homozygous rare predicted loss-of-function variants of OAS1 or RNASEL in two MIS-C patients

**Fig. 1.**
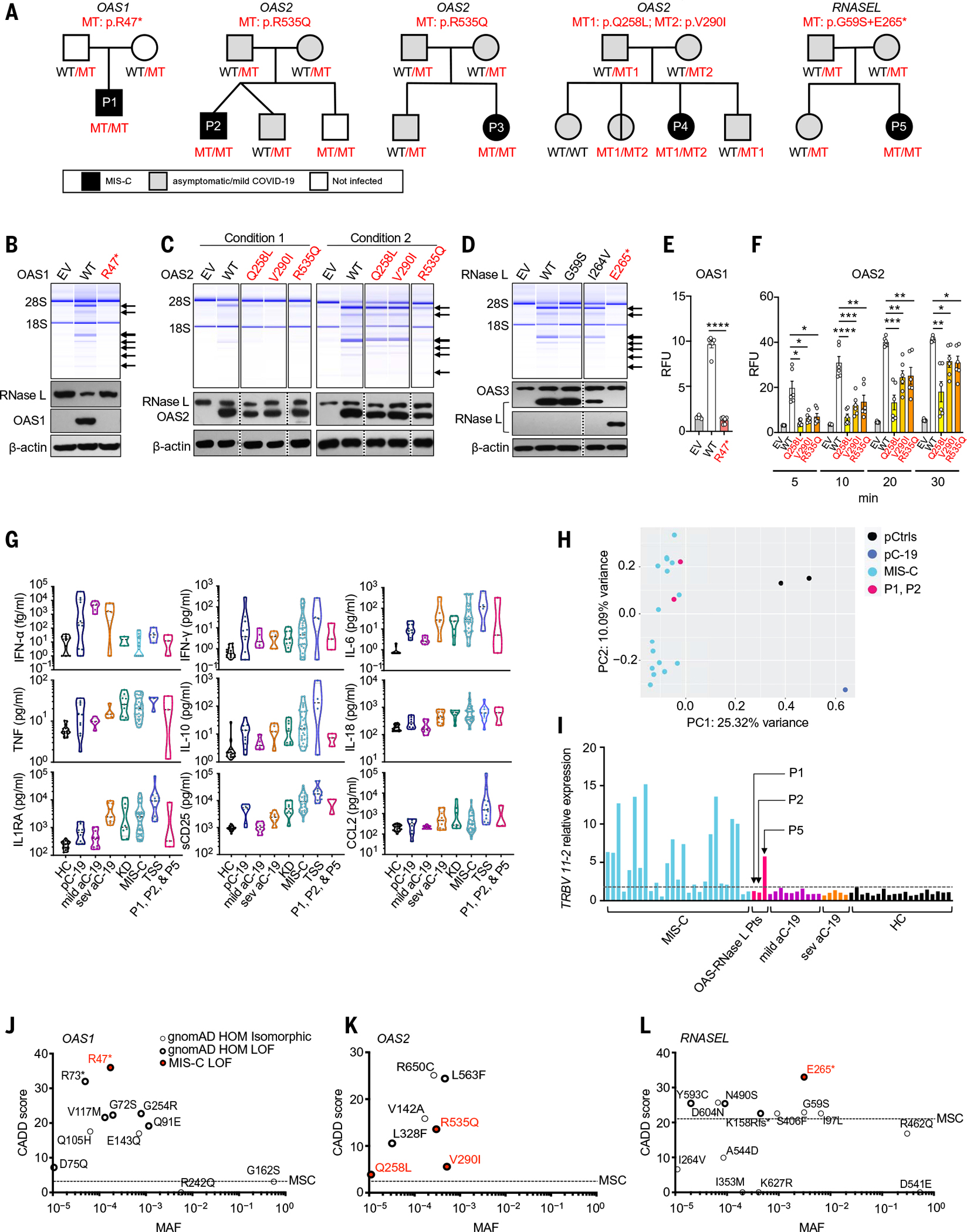
Biallelic *OAS1*, *OAS2*, and *RNASEL* variants in patients with MIS-C. **(A)** Family pedigrees with allele segregation. Mutant, “MT” in red; wild-type, “WT” in black. (**B** to **D**) Functional assays for WT and mutant OAS1 (B), OAS2 (C), and RNase L (D). Variants for which homozygotes or compound heterozygotes were present in our MIS-C cohort were tested. (Upper panels) RNase L–mediated cleavage of rRNA in a cell-free system based on transfected HeLa M cells. (Lower panels) Immunoblots of the indicated proteins. EV, empty vector. Arrows indicate degraded rRNA species. OAS2 variants (C) were tested under two different sets of conditions (see methods). The results shown in (B) to (D) are representative of three independent experiments. (**E** and **F**) FRET assay of 2–5A synthesized in response to poly(I:C) stimulation by WT and MT OAS1 (E) or OAS2 (F). RFU, relative fluorescence units. The data shown are the means ± SEM of six biological replicates. Statistical analysis was performed as described in the methods. *P < 0.05, **P < 0.01, ***P < 0.001, ****P < 0.0001. (**G**) Concentrations of various cytokines in plasma samples from OAS–RNase L–deficient patients during MIS-C (P1, P2, and P5); comparison with those of healthy controls (HC), pediatric (pC-19) or adult COVID-19 pneumonia (aC-19) patients, typical Kawasaki disease patients (KD), other MIS-C patients with no known genetic etiology (MIS-C), and patients with toxic shock syndrome (TSS). (**H**) PCA of gene expression quantified by whole-blood bulk RNA-seq for P1 and P2 during MIS-C relative to pediatric controls (pCtrls), previously published MIS-C patients, and a pediatric patient with mild COVID-19 (pC-19). (**I**) Relative levels of *TRBV 11–2* (encoding Vβ21.3) RNA in blood samples from P1, P2, and P5 during MIS-C, relative to other MIS-C patients, adults with mild or severe COVID-19 (mild aC-19, sev aC-19), and healthy controls. (**J** to **L**) CADD-MAF graph of *OAS1* (J), *OAS2* (K), and *RNASEL* (L) variants for which homozygotes are reported in gnomAD and/or found in our MIS-C cohort. Single-letter abbreviations for the amino acid residues are as follows: A, Ala; C, Cys; D, Asp; E, Glu; F, Phe; G, Gly; H, His; I, Ile; K, Lys; L, Leu; M, Met; N, Asn; P, Pro; Q, Gln; R, Arg; S, Ser; T, Thr; V, Val; W, Trp; and Y, Tyr.

We performed whole-exome or whole-genome sequencing for 558 patients with MIS-C from the international COVID Human Genetic Effort (CHGE) cohort (https://www.covidhge.com/) (fig. S1). We first searched for homozygous or hemizygous rare predicted loss-of-function (pLOF) variants with a high degree of confidence in human genes with a gene damage index of <13.83 (41). We then restricted the list to genes involved in host response to viruses (Gene Ontology term “response to virus,” GO:0009615). We identified two unrelated patients homozygous for stop-gain variants, of *OAS1* in one patient (P1, p.R47*) and *RNASEL* in the other (P5, p.E265*) (Fig. 1A, fig. S2A, and Table 1). OAS1 (2′-5′-oligoadenylate synthetase 1) is one of the four members of the OAS family (OAS1, OAS2, OAS3, and the catalytically inactive OASL). These proteins are type I IFN–inducible cytosolic proteins that produce 2′-5′-linked oligoadenylates (2–5A) upon binding to double-stranded RNA (dsRNA). The 2–5A, in turn, induce the dimerization and activation of the latent endoribonuclease RNase L, which degrades single-stranded RNA (ssRNA) of viral or human origin (42, 43). No homozygous variants fulfilling these criteria were identified in any of the 1288 subjects with asymptomatic or mild SARS-CoV-2 infection (SARS-CoV-2–infected controls) in the CHGE database (fig. S1). MIS-C patients therefore display significant enrichment (*P* = 0.013) in homozygous pLOF variants of the *OAS1* and *RNASEL* genes, suggesting that these loci are probably relevant to MIS-C pathogenesis. Moreover, although OAS1, OAS2, OAS3, and RNase L are expressed in various cell types in mice and humans, their levels are particularly high in myeloid cells, including monocytes and macrophages (44–46). Thus, autosomal recessive (AR) deficiencies of the OAS–RNase L pathway may underlie MIS-C by impairing the restriction of viral replication and/or enhancing the virus-triggered inflammatory response in monocytes, macrophages, dendritic cells, or other cell types.

### Identification of biallelic rare experimentally deleterious variants of OAS1, OAS2, or RNASEL in five MIS-C patients

*OAS1*, *OAS2*, *OAS3*, and *RNASEL* have consensus negative selection (CoNeS) scores for negative selection of 2.25, 0.79, 1.46, and 0.66, respectively, consistent with findings for known monogenic IEIs with an AR mode of inheritance (47). We therefore extended our search to all homozygous or potential compound-heterozygous nonsynonymous or essential splicing site variants with a minor allele frequency (MAF) of <0.01 at these four loci in our MIS-C cohort. We identified a total of 12 unrelated patients and 16 different variants of *OAS1*, *OAS2*, *OAS3*, and *RNASEL* (Table 1). To study the expression and function of these 16 variants in vitro, we first analyzed RNase L–mediated ribosomal RNA (rRNA) degradation after the cotransfection of RNase L–deficient HeLa M cells with the corresponding *OAS1*, *OAS2*, *OAS3*, or *RNASEL* cDNAs (48–51) (Fig. 1, B to D, and fig. S2, B and C). The p.R47* OAS1 (homozygous in P1) mutant protein was not produced and was LOF (Fig. 1B and fig. S2D). The three mutant OAS2 proteins detected (p.R535Q, p.Q258L, and p.V290I) were produced in normal amounts, but p.R535Q (homozygous in P2 and P3) had minimal activity, and p.Q258L and p.V290I (both found in P4) had lower levels of activity than the wild-type (WT) protein (Fig. 1, A and C). All the *OAS3* variants were produced in normal amounts, and all but one (p.R932Q, found in the heterozygous state in one patient) of these variants had normal levels of activity (fig. S2C). The *RNASEL* p.E265* variant (homozygous in P5) was expressed as a truncated protein and was LOF (Fig. 1D and fig. S2E), whereas the p.I264V variant was neutral in expression and function (Fig. 1D). We also quantified the function of the OAS1 and OAS2 mutants in a fluorescence resonance energy transfer (FRET) assay, which confirmed that P1’s *OAS1* variant was LOF and that the *OAS2* variants of P2, P3, and P4 were hypomorphic (21 to 43%, 32 to 76%, and 36 to 75% of WT OAS2 activity for p.Q258L, p.V290I, and p.R535Q, respectively) (Fig. 1, E and F). Thus, we identified five unrelated MIS-C patients homozygous or compound heterozygous for rare and deleterious alleles of three of the four genes controlling the OAS–RNase L pathway (Fig. 1A and fig. S2A). The patients’ genotypes were confirmed by Sanger sequencing and familial segregation. Their clinical and immunological features were consistent with those previously reported for other MIS-C patients (21, 22, 26–36, 52) (Fig. 1, G to I, and Table 2).

### Enrichment in homozygous deleterious OAS1, OAS2, and RNASEL variants in MIS-C patients

We found no homozygous rare (MAF < 0.01) deleterious variants of the three genes in the 1288 SARS-CoV-2–infected controls or in a control cohort of 334 patients under the age of 21 years with asymptomatic or mild infection or COVID-19 pneumonia (fig. S1). Thus, there was a significant enrichment in such homozygotes among MIS-C patients relative to infected controls (*P* = 0.001) or controls under 21 years old (*P* = 0.046), suggesting that AR deficiencies of three genes of the OAS–RNase L pathway (*OAS1*, *OAS2*, and *RNASEL*) specifically underlie MIS-C. We further assessed the probability of AR deficiencies of these three gene products being causal for MIS-C by evaluating the expression and function of all nonsynonymous variants of *OAS1*, *OAS2*, and *RNASEL* for which homozygotes were reported in the Genome Aggregation Database (gnomAD, v2.1.1 and v3.1.1, 28 variants in total) in our RNase L–mediated rRNA degradation assay (fig. S2, F to H, and table S1). In total, 13 *OAS1*, *OAS2*, or *RNASEL* variants were deleterious and present in the homozygous state in 19 individuals in the gnomAD database (Fig. 1, J to L). The estimated cumulative frequency of homozygous carriers of LOF variants at the three loci was ~0.00013 [95% confidence interval (CI): 7.2 × 10^−5^ to 20 × 10^−5^] in the general population. The rarity of AR OAS–RNase L deficiencies in the general population is therefore consistent with that of MIS-C. Moreover, the enrichment in these deficiencies observed in MIS-C patients relative to the individuals included in gnomAD was highly significant (*P* = 2 × 10^−6^). These findings suggest that AR deficiencies of OAS1, OAS2, and RNase L are genetic etiologies of MIS-C.

### The expression pattern for the OAS–RNase L pathway implicates mononuclear phagocytes

**Fig. 2.**
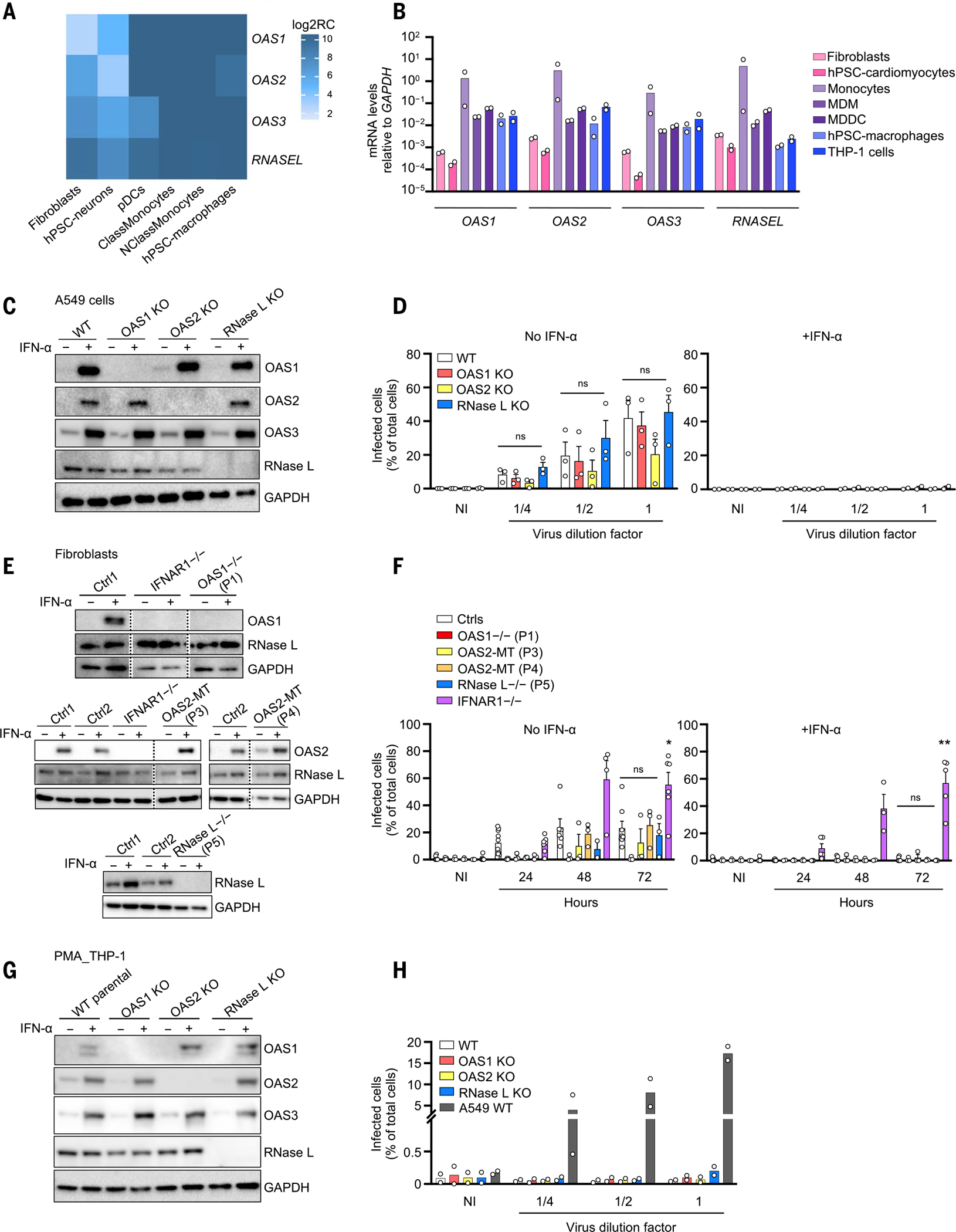
Expression pattern of the OAS–RNase L pathway genes and their role in SARS-CoV-2 restriction. (**A** and **B**) Relative *OAS1*, *OAS2, OAS3*, and *RNASEL* mRNA levels measured by bulk RNA-seq (A) or RT-qPCR (B), in various cell types. hPSC, human pluripotent stem cell; ClassMonocytes, classical monocytes; NClassMonocytes, nonclassical monocytes; MDM, monocyte-derived macrophages; MDDC, monocyte-derived dendritic cells; Log2RC, log_2_ read count. (**C** and **D**) Immunoblot of the indicated proteins (C) and immunofluorescence (IF) of SARS-CoV-2 nucleocapsid (N) protein (D) in A549+ACE2/TMPRSS2 cells with and without knockout (KO) of OAS1, OAS2, or RNase L. IF analysis for N protein was performed 24 hours after infection with various dilutions of SARS-CoV-2. Dilution factors of ¼, ½, and 1 correspond to MOI values of 0.0002, 0.0005, and 0.001, respectively. GAPDH, glyceraldehyde-3-phosphate dehydrogenase; NI, noninfected. (**E** and **F**) Immunoblot of the indicated proteins (E) and IF analysis for the SARS-CoV-2 N protein (F) in SV40-fibroblasts +ACE2 from healthy controls (Ctrl1 and Ctrl2), patients with *OAS-RNASEL* mutations (P1, P3, P4, and P5), and a previously reported patient with complete IFNAR1 deficiency (*IFNAR1*^−/−^). IF analysis for N protein was performed at various time points after infection at a MOI of 0.08. (**G** and **H**) Immunoblot of the indicated proteins (G) and IF analysis for the SARS-CoV-2 N protein (H) in THP-1 cells with and without KO of OAS1, OAS2, or RNase L. IF analyses for N protein were performed in PMA-primed THP-1 cells 24 hours after infection with various dilutions of SARS-CoV-2. Dilution factors of ¼, ½, and 1 correspond to MOI values of 0.012, 0.025, and 0.05, respectively. WT A549+ACE2/TMPRSS2 cells were included as a positive control for SARS-CoV-2 infection. The data points are means ± SEM from three [(D) and (F)] or means from two [(B) and (H)] independent experiments with three to six technical replicates per experiment. Statistical analyses were performed as described in the methods. ns, not significant; *P < 0.05, **P < 0.01.

We studied the basal expression of *OAS1*, *OAS2*, *OAS3*, and *RNASE*L in cells from different tissues. Consistent with data from public databases (44), our in-house human cell RNA sequencing (RNA-seq) and reverse transcription–quantitative polymerase chain reaction (RT-qPCR) data showed that myeloid blood cells had higher basal mRNA levels for the four genes than did the tissue-resident cells tested (Fig. 2, A and B). In all cell types studied, both type I and type II IFN treatments up-regulated the levels of mRNA for *OAS1*, *OAS2*, and *OAS3*, whereas the levels of *RNASEL* mRNA were not influenced by these IFNs (fig. S3A). Previous studies reported a relationship between cell type–dependent activation of the OAS–RNase L pathway and basal levels of expression in mice (45, 46). MIS-C occurs 3 to 6 weeks after SARS-CoV-2 infection, but the virus and/or viral proteins may still be detectable in nonrespiratory tissues, such as the intestine or heart, at disease onset in some patients (32, 34, 37). In addition, CD4^+^ and CD8^+^ T cells carrying Vβ21.3 expand, which implies a superantigen-like viral driver of MIS-C (32, 34, 36–38) and suggests that the virus or its antigens persist. Thus, AR deficiencies of the OAS–RNase L pathway may underlie MIS-C by impairing SARS-CoV-2 restriction and/or enhancing virus-triggered inflammatory responses in monocytes and other mononuclear phagocytes.

### OAS–RNase L deficiencies have no impact on SARS-CoV-2 replication in A549 epithelial cells and fibroblasts

Previous studies have shown that the overproduction of exogenous OAS1 can result in the restriction of SARS-CoV-2 replication in A549 lung epithelial cells in the absence of exogenous type I IFN (53, 54). However, the five OAS–RNase L–deficient patients had MIS-C without pneumonia. We assessed SARS-CoV-2 replication in A549 cells rendered permissive to SARS-CoV-2 by the stable expression of angiotensin-converting enzyme 2 (ACE2) and transmembrane protease serine 2 (TMPRSS2), which facilitates viral entry. Knockout (KO) of OAS1 or OAS2 did not increase the proportion of SARS-CoV-2–infected cells at 24 or 48 hours relative to that for the parental WT A549 cells, regardless of the presence or absence of exogenous IFN-α2b (Fig. 2, C and D, and fig. S3B). Only RNase L KO cells resulted in a mild increase in susceptibility to SARS-CoV-2 relative to WT cells in the absence of IFN-α2b, consistent with previous findings (55). We also used patient-specific SV40-transduced human dermal fibroblasts (SV40-fibroblasts) stably expressing ACE2 as a surrogate cell type for studying the impact of OAS–RNase L deficiencies on tissue-resident cell-intrinsic defenses against SARS-CoV-2 (5). Consistent with the lack of pneumonia in these patients, no increase in SARS-CoV-2 susceptibility was observed in any of the fibroblasts with *OAS1* (from P1), *OAS2* (P3 and P4), or *RNASEL* (P5) mutations up to 72 hours after infection in the presence or absence of exogenous IFN-α2b, despite the complete loss ofexpression of OAS1 or RNase L in the cells of P1 and P5, respectively (Fig. 2, E and F). This contrasted with the increased susceptibility reported for fibroblasts from a patient with AR complete IFNAR1 deficiency (56) and critical COVID-19 pneumonia.

### OAS–RNase L deficiencies have no impact on SARS-CoV-2 replication in THP-1 cells

Only abortive SARS-CoV-2 infection has been reported in human mononuclear phagocytes, including monocytes and macrophages, which express very little to no ACE2 (57–59). However, basal *Oas* and *Rnasel* expression levels have previously been correlated with murine coronavirus or vesicular stomatitis virus (VSV) restriction in mouse macrophages (60). We tested the hypothesis that deficiencies of OAS–RNase L might result in productive SARS-CoV-2 infection in mononuclear phagocytes by assessing the replication of SARS-CoV-2. Unlike WT A549 cells stably transduced with ACE2 and TMPRSS2, in which SARS-CoV-2 can be detected 24 hours after infection, no SARS-CoV-2 was detected in THP-1–derived macrophages (61), whether parentalor with a KO of OAS1, OAS2, or RNase L (Fig. 2, G and H, and fig. S3C). Thus, no myeloid SARS-CoV-2 replication was detected in the presence or absence of deficiencies of the OAS–RNase L pathway, at least in this cellular model of mononuclear phagocytes (60).

### OAS–RNase L deficiencies result in an exaggerated inflammatory response to intracellular dsRNA in THP-1 cells

**Fig. 3.**
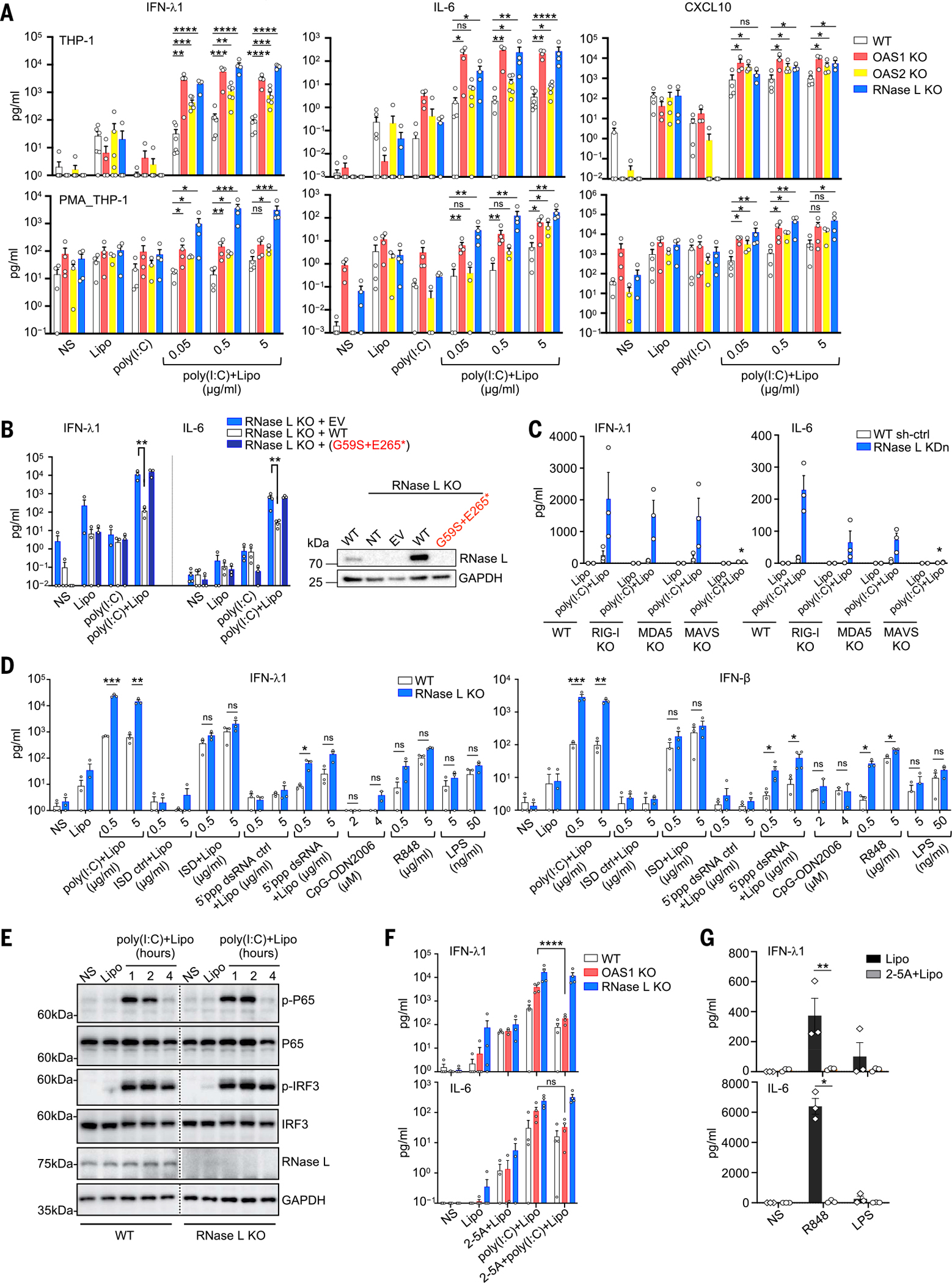
Exaggerated inflammatory responses of OAS–RNase L-deficient THP-1 cells. (**A**) Concentrations of various cytokines in the supernatant of OAS1 KO, OAS2 KO, RNase L KO, or parental THP-1 cells (upper panels) or PMA-primed THP-1 cells (lower panels) treated as indicated for 24 hours. (**B**) IFN-λ1 and IL-6 concentrations in the supernatant of RNase L KO THP-1 cells transduced with the WT or P5’s variant RNASEL cDNA, or empty vector (EV), and treated as indicated for 24 hours. On the right, RNase L protein levels, as assessed by immunoblotting. NT, not transfected. (**C**) IFN-λ1 and IL-6 concentrations in the supernatant of parental, RIG-I KO, MDA5 KO, or MAVS KO THP-1 cells with or without (WT sh-ctrl) RNase L knockdown (KDn), treated as indicated for 24 hours. (**D**) IFN-λ1 and IFN-β concentrations in the supernatant of parental or RNase L KO THP-1 cells, treated as indicated for 24 hours. (**E**) Immunoblot of phosphorylated P65 and IRF3 in parental and RNase L KO THP-1 cells treated as indicated. The results shown are representative of two independent experiments. (**F**) IFN-λ1 and IL-6 concentrations in the supernatant of parental, OAS1 KO, or RNase L KO THP-1 cells treated as indicated for 24 hours. (**G**) IFN-λ1 and IL-6 concentrations in WT THP-1 cells treated as indicated for 24 hours. In (A) to (D), (F), and (G), the data points are means ± SEM from three to five independent experiments with one to two technical replicates per experiment. Statistical analysis was performed as described in the methods. ns, not significant; *P < 0.05, **P < 0.01, ***P < 0.001, ****P < 0.0001. NS, nonstimulated; Lipo, lipofectamine only; poly(I:C), extracellularly added poly(I:C); poly(I:C)+Lipo, intracellular poly(I:C) in the presence of lipofectamine; 2–5A+Lipo, intracellular 2–5A in the presence of lipofectamine; 2–5A+poly(I:C)+Lipo, intracellular poly(I:C) in addition to intracellular 2–5A.

Sustained monocyte activation has repeatedly been reported to be a key immunological feature of MIS-C (22, 31–36). We studied the impact of OAS–RNase L deficiencies on cellular responses to intracellular (cytosolic) or extracellular (endosomal) stimulation with dsRNA in THP-1 cells. Consistent with a previous study (62), THP-1 cells and THP-1–derived macrophages with a KO for OAS1, OAS2, or RNase L displayed enhanced activation, as demonstrated by their higher levels of IFN-λ1, IFN-β, IL-1β, IL-6, CXCL9, CXCL10, and TNF secretion 24 hours after stimulation with various doses of intracellular polyinosinic:polycytidylic acid [poly(I:C)] (Fig. 3A and fig. S4A), as well as higher mRNA induction for *IL6* and *CXCL9* 8 hours after stimulation (fig. S4, B and C). Cell viability was similar to that of WT THP-1 cells after intracellular poly(I:C) stimulation (fig. S4D). Small hairpin RNA–mediated knockdown (KDn) of the expression of *OAS1*, *OAS2*, and *RNASEL* in THP-1 cells confirmed these findings (fig. S4E). The transduction of THP-1 cells with a KO of the corresponding gene with the WT cDNA of *OAS1*, *OAS2*, or *RNASEL*, respectively, resulted in cytokine secretion levels similar to those observed in parental cells, whereas transduction with mutant cDNAs corresponding to the patients’ variants had no such effect (*OAS1* variant of P1 and *RNASEL* variant of P5) or a lesser effect (*OAS2* variants of P2, P3, and P4) (Fig. 3B and fig. S5, A to C). Thus, OAS–RNase L deficiencies result in exaggerated inflammatory responses to intracellular dsRNA stimulation in THP-1 cells. Enhanced responses may also occur in the mononuclear phagocytes of our patients, underlying MIS-C.

### The inflammatory response to intracellular dsRNA in THP-1 cells is MAVS dependent

Intracellular dsRNA is known to stimulate the RIG-I/MDA5–MAVS pathway (RIG-I, retinoic acid–inducible gene I; MDA5, melanoma differentiation-associated protein 5; MAVS, mitochondrial antiviral-signaling protein), inducing type I IFNs and other cytokines in various cell types (63), in addition to the OAS–RNase L pathway (42, 64). Indeed, unlike WT THP-1 cells, MAVS KO THP-1 cells did not respond to intracellular poly(I:C) stimulation, and *RNASEL* gene KDn did not result in enhanced activation (Fig. 3C and fig. S5, D and E), confirming that the response to poly(I:C) is dependent on MAVS-mediated signaling in these cells. The enhancement of the intracellular poly(I:C) response after *RNASEL* KDn was partially attenuated in RIG-I or MDA5 KO THP-1 cells (Fig. 3C and fig. S5, D and E), suggesting that both dsRNA sensors may be involved. Another dsRNA agonist that specifically activates RIG-I, 5′ triphosphate double-stranded RNA (5′ppp-dsRNA), induced enhanced responses in RNase L KO THP-1 cells similar to those seen with poly(I:C) (Fig. 3D). By contrast, the activation of other sensing pathways, including the extracellular ssRNA-sensing toll-like receptor 7 (TLR7) and TLR8 pathways (R848), the TLR4 pathway (LPS), and the intracellular DNA agonist-sensing DAI pathway (ISD), resulted in responses in RNase L KO or KDn THP-1 cells that were similar to those of the parental WT cells (Fig. 3D and fig. S5F). Thus, the exaggerated inflammatory responses to cytosolic dsRNA observed in THP-1 cells deficient for OAS–RNase L appear to require RIG-I/MDA5 sensing and MAVS activation.

### Activation of the OAS–RNase L pathway can suppress inflammatory responses in THP-1 cells

Intracellular dsRNA stimulates both the RIG-I/MDA5–MAVS and OAS–RNase L pathways (42, 63, 64). We therefore investigated whether the dsRNA-sensing MAVS-dependent signaling pathway was itself hyperactivated as a result of OAS–RNase L deficiency. After intracellular poly(I:C) stimulation, interferon regulatory factor 3 (IRF3) and nuclear factor κB (NF-κB) phosphorylation levels were similar in RNase L KO and WT THP-1 cells (Fig. 3E). Thus, the molecular mechanisms by which OAS–RNase L deficiency results in an exaggerated inflammatory response appears to involve an impairment of RNase L activation resulting in a lack of host RNA transcriptional and/or translational inhibition (65–68), rather than a hyperactivation of the MAVS-dependent pathways. Consistent with this hypothesis, treatment with exogenous 2–5A, which is normally generated by OASs upon dsRNA sensing and activates RNase L (42, 43), rescued the inflammatory phenotype in OAS1 KO THP-1 cells after intracellular poly(I:C) stimulation (Fig. 3F). By contrast, dephosphorylated 2–5A, which is unable to activate RNase L (69, 70), had no such effect (fig. S5G). Moreover, exogenous 2–5A treatment decreased the response to TLR7/8 activation in WT THP-1 cells (Fig. 3G). Treatment with 2–5A had a much weaker effect or even no suppressive effect in RNase L KDn or KO THP-1 cells (Fig. 3F and fig. S5, G and H). Thus, the exaggerated inflammatory response in OAS–RNase L–deficient mononuclear cells appears to result from the activation of the MAVS-dependent pathway (but not of other nucleic acid–sensing pathways) and an impairment of RNase L activation by OAS1- or OAS2-derived 2–5A after dsRNA sensing. This imbalance createsa phenotype that is probably a consequence of an impairment of the posttranscriptional activities of RNase L (65–68).

### OAS–RNase L deficiencies result in an exaggerated inflammatory response to SARS-CoV-2 in THP-1 cells

**Fig. 4.**
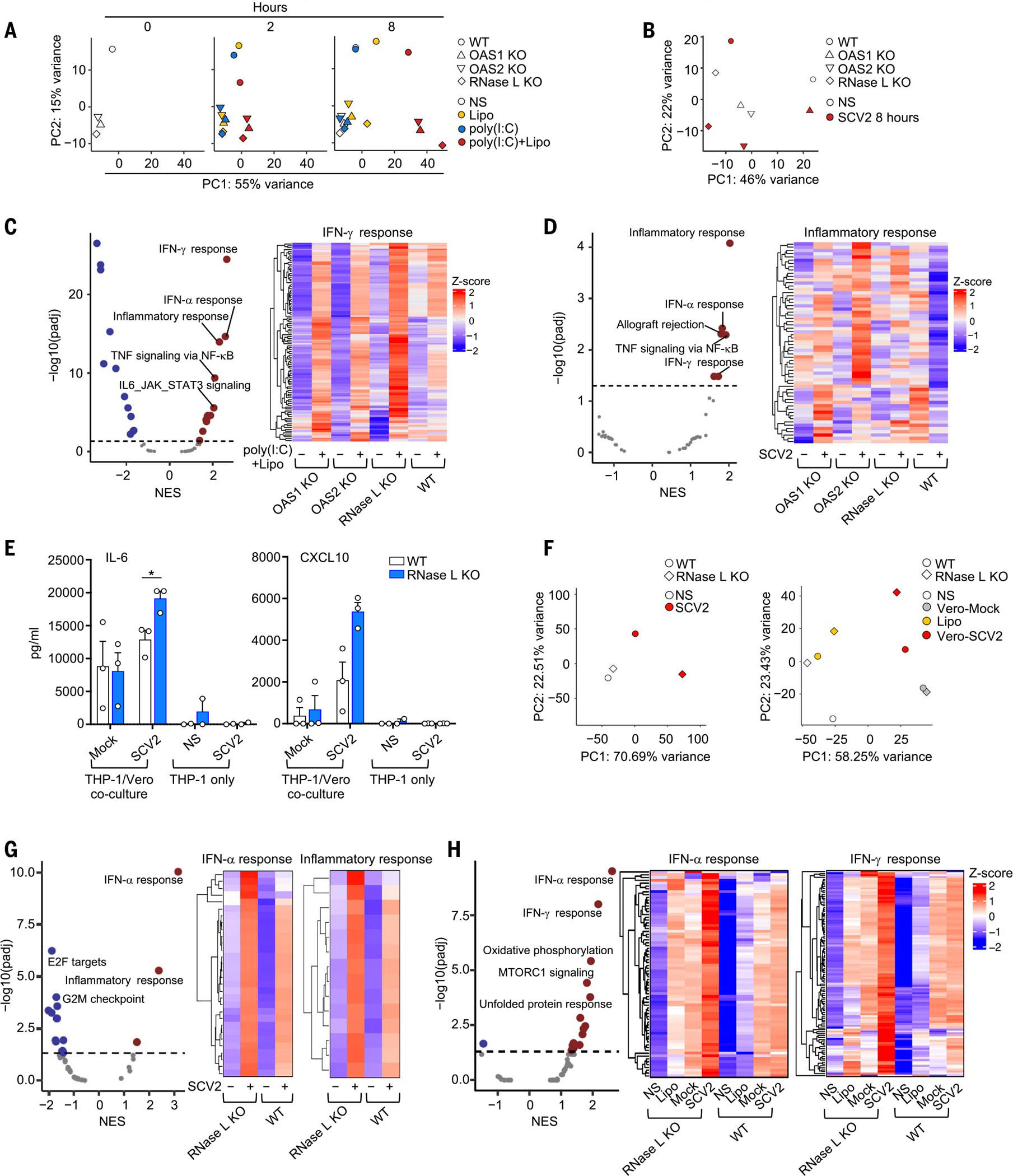
Exaggerated inflammatory responses to SARS-CoV-2 of OAS–RNase L–deficient THP-1 cells. (**A** and **B**) PCA of RNA-seq–quantified gene expression for OAS1 KO, OAS2 KO, RNase L KO, and parental (WT) THP-1 cells left nonstimulated (NS), treated as indicated for 2 or 8 hours (A), or stimulated with SARS-CoV-2 (SCV2) at a MOI of 0.01 for 8 hours (B). (**C** and **D**) Differential expression analysis (DEA) and gene set enrichment analysis (GSEA) for genes induced by 8 hours of intracellular poly(I:C) stimulation (C) or by 8 hours of SCV2 stimulation (D). The OAS1 KO, OAS2 KO, and RNase L KO THP-1 cells were compared with parental (WT) THP-1 cells. Volcano plots show immune system–related pathways. NES, normalized enrichment score. Heatmaps show gene expression for the “IFN-γ response” (C) or “inflammatory response” (D) Hallmark gene sets. (**E**) IL-6 and CXCL10 concentrations in the supernatant of parental or RNase L KO THP-1 cells treated as indicated for 24 hours. The data points are means ± SEM from three independent experiments with three technical replicates per experiment. Statistical analysis was performed as described in the methods. *P < 0.05. (**F**) PCA of RNA-seq–quantified gene expression, for RNase L KO and parental THP-1 cells cocultured with Vero cells with or without SCV2 infection for 24 hours (left) or transfected for 8 hours with RNA from Vero cells with or without SCV2-infection (right). (**G** and **H**) DEA and GSEA for genes induced in RNase L KO THP-1 cells, compared with parental THP-1 cells after 24 hours of coculture with SCV2-infected or mock-infected Vero cells (G), or after 8 hours of transfection with RNA from SCV2-infected or mock-infected Vero cells (H). Volcano plots show immune system–related pathways. Heatmaps show gene expression for the indicated Hallmark gene sets. Heatmaps represent Z-score–scaled log_2_ read counts per million. NS, nonstimulated; Lipo, lipofectamine; SCV2, SARS-CoV-2.

We investigated whether OAS–RNase L deficiencies resulted in exaggerated inflammatory responses to SARS-CoV-2 in mononuclear phagocytes. Bulk RNA-seq on THP-1 cells with KO of OAS1, OAS2, or RNase L stimulated with intracellular poly(I:C) or SARS-CoV-2 revealed transcriptomic profiles different from those of the parental cells (Fig. 4, A and B, and fig. S6A). Gene set enrichment analysis (GSEA) against Hallmark gene sets (71) revealed an enrichment in genes relating to inflammatory responses and IFN-γ signaling in OAS–RNase L–deficient cells, showing that these cells displayed an exacerbated inflammatory response not only to synthetic dsRNA but also to SARS-CoV-2 (Fig. 4, C and D). Moreover, RNase L KO THP-1 cells had higher levels of IL-6 and CXCL10 secretion than WT cells when coculturedwithSARS-CoV-2–infected Vero cells, which support SARS-CoV-2 replication (72, 73) (Fig. 4E and fig. S6, B and C). Bulk RNA-seq further confirmed this observation at the transcriptome level (Fig. 4F and fig. S6D), revealing an enrichment in the expression of genes relating to inflammatory responses and IFN-α signaling in RNase L KO cells relative to WT cells (Fig. 4G). In addition, transfection with total RNA from SARS-CoV-2–infected Vero cells, but not from uninfected Vero cells, also induced enhanced responses in RNase L KO THP-1 cells relative to parental WT cells, with an enrichment in genes relating to inflammatory responses and IFN-γ signaling (Fig. 4H and fig. S6E). These findings suggest that OAS–RNase L deficiency results in excessive inflammatory responses in mononuclear phagocytes following both abortive SARS-CoV-2 infection and coculture with SARS-CoV-2–replicating cell types. This is likely due to defective activation of the OAS-RNase L pathway following the engulfment of the virus or infection-related by-products, leading to the release of dsRNA into the cytosol (73).

### OAS–RNase L deficiencies result in an enhanced inflammatory response to intracellular dsRNA in primary mononuclear cells

**Fig. 5.**
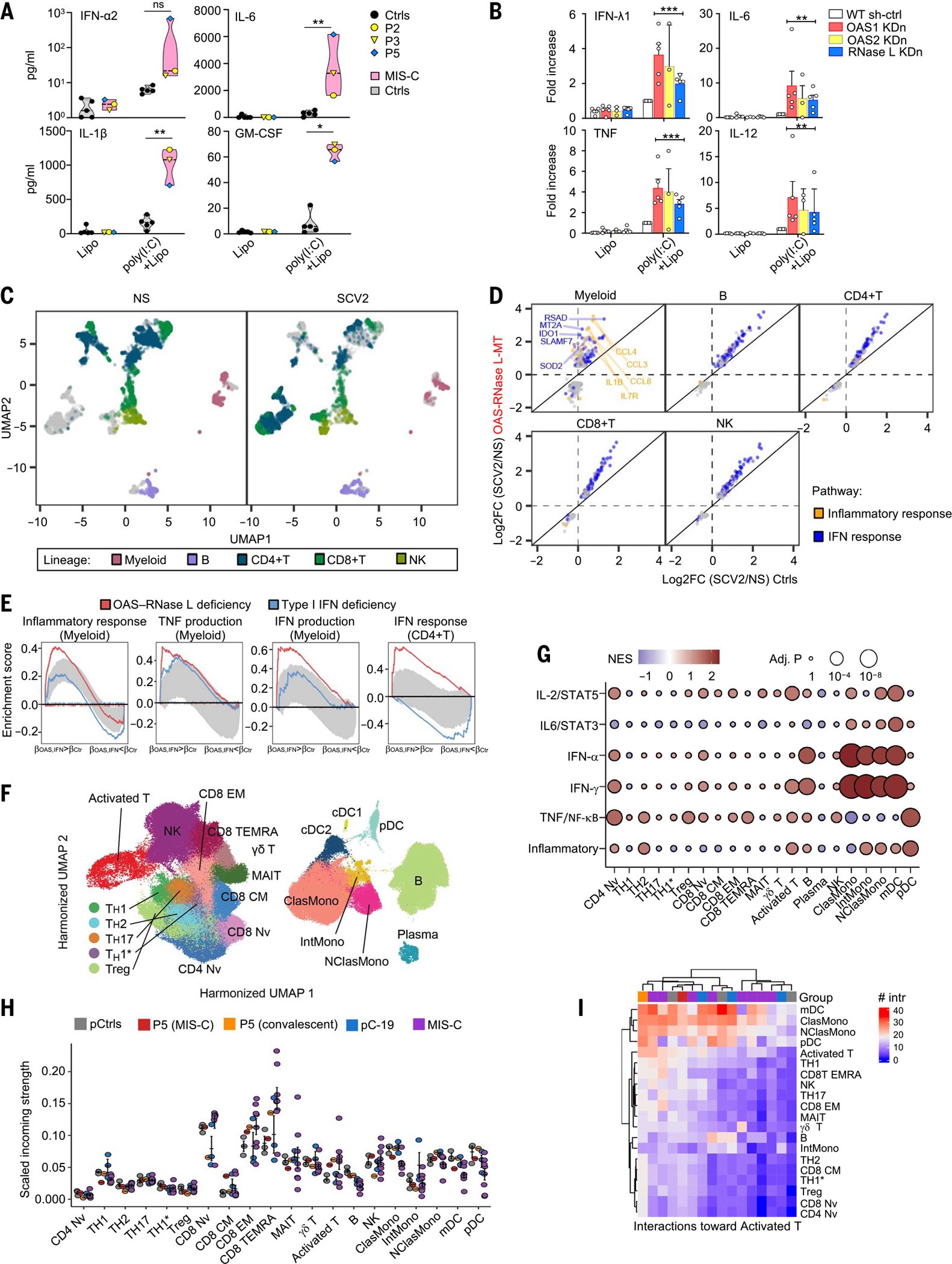
Exaggerated myeloid cell activation in response to SARS-CoV-2 underlies MIS-C. **(A)** Concentrations of cytokines in the supernatant of PBMCs from OAS–RNase L–deficient patients (grouped in the pink violin zone) and three healthy pediatric and two healthy adult controls (Ctrls; gray violin zone). The data points are means of biological duplicates. **(B)** Fold-increase in the concentrations of cytokines in the supernatant of MDDCs with KDn of OAS1, OAS2, or RNase L, or transduced with control shRNA (WT sh-ctrl). The fold-change is expressed relative to the values for poly(I:C)+lipostimulated WT sh-ctrl cells. Data shown are means ± SEM from three independent experiments, with one to two technical replicates per experiment. For (A) and (B), statistical analysis was performed as described in the methods. NS, nonstimulated. ns, not significant; *P < 0.05, **P < 0.01, ***P < 0.001. (**C** to **E**) scRNA-seq of PBMCs from OAS–RNase L–deficient patients (OAS–RNase L-MT) or healthy controls after 6 hours of incubation with SARS-CoV-2 (SCV2) or mock infection (NS). (C) Uniform manifold approximation and projection (UMAP) of single PBMC transcriptomes. (D) Cell type–specific transcriptional responses. Genes passing the FDR < 0.01 and |log2FC| > 0.5 thresholds are shown. (E) GSEA of SCV2-induced genes across immune-related Hallmark gene sets. PBMCs from three patients with type I IFN pathway deficiency are controls for defective type I IFN responses. Gray zone highlights the expected enrichment scores under the null hypothesis (95% CI calculated over 100 randomized genes). (**F** to **I**) scRNA-seq of PBMCs from P5 and from healthy controls. A published dataset for pediatric patients with acute SARS-CoV-2 infection (pC-19) and MIS-C was also integrated. (F) UMAP of clustering analysis. (G) Pseudobulk differential expression analysis with GSEA. P5 (convalescent phase) was compared with local pediatric controls (pCtrls). Immune-related pathways are shown. [(H) and (I)] Intercellular communication analysis with CellChat. (H) Incoming signal strength and (I) the number of interactions for representative cell subsets.

We then studied the impact of OAS–RNase L deficiencies on the response to intracellular poly(I:C) stimulation in human peripheral blood mononuclear cells (PBMCs). Routine blood cell counts and immunotyping for the five patients revealed no significant abnormalities in blood leukocyte subsets, a result confirmed by deep immunophenotyping by mass cytometry [cytometry by time of flight (CyTOF)] (fig. S7A and table S2). After intracellular poly(I:C) stimulation, PBMCs from P2 (OAS2 deficient), P3 (OAS2 deficient), and P5 (RNase L deficient) secreted larger amounts of the inflammatory cytokines studied than cells from healthy controls (Fig. 5A and fig. S7B). This enhanced inflammatory response to intracellular poly(I:C) stimulation was monocyte dependent, as the depletion of monocytes from the PBMCs of healthy controls strongly decreased this response (fig. S7C). Moreover, the shRNA-mediated KDn of *OAS1*, *OAS2*, or *RNASEL* in monocyte-derived dendritic cells (MDDCs) from healthy controls resulted in an enhanced inflammatory response to intracellular poly(I:C) stimulation, as shown by the higher levels of inflammatory cytokines, including IFN-λ1, IL-6, TNF, and IL-12, than were observed with WT parental cells (Fig. 5B). Thus, deficiencies of the OAS–RNase L pathway also result in exaggerated inflammatory responses to intracellular dsRNA stimulation in primary mononuclear phagocytes, or at least in monocytes and MDDCs.

### Enhanced myeloid cell activation by SARS-CoV-2 in patient PBMCs

We studied the impact of OAS–RNase L deficiencies on the responses of the various PBMC populations to SARS-CoV-2 by performing single-cell RNA sequencing (scRNA-seq) on PBMCs from P1 (OAS1), P2 (OAS2), P3 (OAS2), and P5 (RNase L) and comparing the results with those for healthy controls. Regardless of genotype, 6 hours of stimulation with SARS-CoV-2inducedastrongimmuneresponseacross all five major immune cell types including myeloid, B, CD4^+^ T, CD8^+^ T, and natural killer (NK) cells (Fig. 5C), with 1301 unique differentially expressed genes (DEGs) (data S1). OAS–RNase L deficiency significantly changed the response of 48 to 94% of the DEGs in each lineage, with myeloid cells being the most affected. Cellular responses were generally stronger in the OAS–RNase L–deficient patients and were essentially limited to the IFN-α and IFN-γ response pathways. Myeloid cell responses were characterized by a distinct proinflammatory component, such as *IL1B* and *CCL3*, that was stronger in OAS–RNase L–deficient cells (Fig. 5D and data S2). We then calculated pseudobulk estimates by cell type. Consistent with the single-cell observations, genes strongly up-regulated by SARS-CoV-2 in OAS–RNase L–deficient myeloid cells were enriched in types I and II IFN signature genes and TNF signature genes, whereas those strongly up-regulated in CD4^+^ T cells were enriched in type I IFN signature genes (Fig. 5E). Thus, there is an exaggerated inflammatory response to intracellular dsRNA or extracellular SARS-CoV-2 stimulation in primary monocytes and other mononuclear phagocytes with deficiencies of the OAS–RNase L pathway cultured alone or with other PBMC populations. This provides a plausible pathogenic mechanism for MIS-C, in which this condition is driven by the exacerbated activation of mononuclear phagocytes. This hypothesis is also supported by scRNA-seq on PBMCs from P5 (RNase L deficient) collected during MIS-C and the convalescence period. Enhanced expression levels were observed for IFN-α, IFN-γ, or TNF signature genes in monocytes, myeloid dendritic cells (mDCs), B lymphocytes, plasmacytoid dendritic cells (pDCs), and activated T cells of P5 relative to healthy pediatric controls (Fig. 5, F and G, and fig. S8, A to D). Quantitatively inferred cell–cell communications (74) revealed that MIS-C in the RNase L–deficient patient was probably driven by a signal from hyperactivated monocytes and mDCs directed at CD8^+^ αβ T cells (Fig. 5, H and I, and fig. S8, E to G). This situation differs from that observed in patients with COVID-19 pneumonia without MIS-C but is similar to reports for previously described MIS-C patients (fig. S9) (33, 34, 36), identifying exaggerated myeloid cell activation due to OAS–RNase L deficiency as the core driver of the immunological and clinical phenotypes of MIS-C in our patients.

## Discussion

We report AR deficiencies of OAS1, OAS2, and RNase L as genetic etiologies of MIS-C in five unrelated children, corresponding to ~1% of the international cohort of patients studied. OAS–RNase L–deficient monocytic cell lines, monocyte-derived dendritic cells modeling patient genotypes, and primary monocytes from patients displayed excessive inflammatory responses to intracellular dsRNA, SARS-CoV-2, SARS-CoV-2–infected cells, and their RNA, providing a plausible mechanism for MIS-C. In these patients, MIS-C may result primarily from an excessive response of monocytes and other mononuclear phagocytes to SARS-CoV-2 dsRNA intermediates or by-products, followed by the presentation of a viral superantigen to T cells, resulting in the activation and expansion of Vβ21.3^+^ CD4^+^ and CD8^+^ Tcells. The molecular basis of the exacerbated inflammatory response to SARS-CoV-2 due to OAS–RNase L deficiency in mononuclear phagocytes involves an impairment of the activation of RNase L by the dsRNA-sensing molecules OAS1 and OAS2, probably resulting in defective post-transcriptional RNase L activity (67, 68) and the unchecked RIG-I/MDA5–MAVS–mediated production of inflammatory cytokines. Alternative molecular mechanisms cannot be excluded (64, 75). The SARS-CoV-2–related RNA products that trigger phagocyte activation, the viral superantigen(s) that activate T cells, and the human leukocyte antigen (HLA) restriction elements all remain to be discovered. Our findings also do not exclude the possibility that AR OAS–RNase L deficiency additionally affects antiviral responses in cells of other tissues injured during MIS-C, such as cardiomyocytes, enterocytes, and endothelial cells. The role of this pathway in T cells themselves merits further investigation. MIS-C in other patients may result from IEIs that may or may not be related to the OAS–RNase L pathway. Our findings also suggest that other forms of Kawasaki disease may be caused by other virus-specific IEIs in other patients (15).

The notion that the OAS–RNase L pathway is essential for antiviral immunity in mononuclear phagocytic cells was first proposed nearly 40 years ago (60). Intriguingly, the OAS–RNase L pathway is apparently dispensable for protective immunity to SARS-CoV-2 in the respiratory tract. None of the five MIS-C patients had a pulmonary phenotype, and no viral replication was detectable in the upper respiratory tract of any of the five children at the onset of MIS-C. Nevertheless, genomewide association studies have suggested that common variants in the vicinity of *OAS1* may be weakly associated with COVID-19 severity (10, 11, 53, 76–79). Our finding that the human OAS–RNase L pathway is crucial for regulation of the mononuclear phagocyte response to SARS-CoV-2, but not for SARS-CoV-2 restriction in the respiratory tract, suggests that the main protective action of this pathway is mediated by the control of phagocyte-driven systemic inflammation at a later stage of disease rather than viral restriction in the respiratory tract early on. These findings are also consistent with the discovery of germline gain-of-function *OAS1* mutations in humans with an autoinflammatory syndrome involving myeloid cells (80, 81).

The five patients, now aged 1 to 15 years, are normally resistant to diseases caused by other common viruses. Since the discovery of the OAS–RNase L pathway in the 1970s (65, 82, 83), this pathway has been one of the most intensively studied type I IFN–inducible pathways (42, 84). Biochemically, the three OASs have different subcellular distributions and different dsRNA optima for activation, they synthesize 2–5A of different lengths (42, 85), and they appear to have antiviral activity against different viruses (86–88). The only well-established function of 2–5A is the activation of RNase L (66), and any of the three OASs appears to be sufficient for the biochemical activation of RNase L in human cells in vitro. RNase L has been shown to have antiviral activity against certain viruses (dengue virus and Sindbis virus), but not others (Zika virus), in murine and human cells in vitro (85, 89). In vivo RNase L deficiency in mice drives susceptibility to various viruses (e.g., encephalomyocarditis virus, coxsackievirus B4, murine coronavirus, etc.) (45, 85). Our data suggest that human OAS1, OAS2, and RNase L are each essential for the correct regulation of immunity to SARS-CoV-2 but are otherwise largely redundant in natural conditions of infection. It is also clear that the RNase L–dependent functions of OAS1 and OAS2 are crucial for the regulation of immunity to SARS-CoV-2 within the same cells, as the genetic deficiency of any of these three components results in the same immunological clinical phenotype, namely MIS-C.

## Materials and methods

### Patients

We enrolled an international cohort of 558 MIS-C patients (aged 3 months to 19 years, 60.4% boys and 39.6% girls) originating from Europe, Africa, Asia, and America and living in 16 different countries. All patients met the WHO diagnostic criteria for MIS-C (52). We focus here on five of these patients (P1 to P5). Written informed consent was obtained in the country of residence of each patient, in accordance with local regulations and with institutional review board (IRB) approval. Experiments were conducted in the United States and in France, in accordance with local regulations and with the approval of the IRB of the Rockefeller University and the Institut National de la Santé et de la Recherche Médicale, respectively. Approval was obtained from the French Ethics Committee (Comité de Protection des Personnes), the French National Agency for Medicine and Health Product Safety, the Institut National de la Santé et de la Recherche Médicale in Paris, France (protocol no. C10–13), and the Rockefeller University Institutional Review Board in New York, USA (protocol no. JCA-0700). For patients sequenced by National Institute of Allergy and Infectious Diseases (NIAID) through the American Genome Center (TAGC) other than the five patients described in this paper, written informed consent was obtained in the country of residence of each patient, in accordance with local regulations and with IRB approval: Ethics Committee of the Fondazione IRCCS Policlinico San Matteo, Pavia, Italy (protocol 20200037677); Comitato Etico Interaziendale A.O.U. Città della Salute e della Scienza di Torino, Turin, Italy (protocol 00282/2020); and IRB at Children’s Hospital of Philadelphia (protocol 18–014863).

The five patients with MIS-C and AR deficiencies of the OAS–RNase L pathway—two boys and three girls—ranged in age from 3 months to 14 years at the time of diagnosis and all fulfilled the WHO criteria for MIS-C (Table 2) (52). They originated from the Philippines (P1), Spain (P2), Turkey (P3 and P4), and Canada (of French descent) (P5) and lived in Spain, Turkey, and Canada. P1 (*OAS1* mutation) (29), P3 (OAS2), and P4 (OAS2) had a severe course of MIS-C, with coronary aneurysm, myocarditis, and polyneuropathy, respectively. P2 (*OAS2*) and P5 (*RNASEL*) had a milder course of MIS-C, with a typical Kawasaki disease presentation. None of these patients presented any clinical or radiological evidence of pneumonia. Cytokine profiling of serum obtained from P1, P2, and P5 during MIS-C revealed high levels of IFN-γ, soluble CD25, IL-18, IL-1RA, and MCP1 (CCL2) (Fig. 1G), consistent with previously published immune profiles of MIS-C and in contrast to those for pulmonary COVID-19 (21). Bulk mRNA sequencing (RNA-seq) of whole-blood RNA from P1 and P2 collected during the MIS-C phase revealed transcriptomic signatures clearly different from those of healthy controls and a pediatric case of acute COVID-19 pneumonia, but similar to those of previously reported MIS-C patients (Fig. 1H) (33). T cell receptor Vβ repertoire analysis confirmed the expansion of *TRBV 11–2* (encoding Vβ21.3) in one of the three MIS-C–phase samples available (P5, with AR RNase L deficiency) (Fig. 1I). The clinical and immunological features of the five patients were, therefore, consistent with those previously reported for other MIS-C patients (21, 22, 26–36).

### Whole-exome, whole-genome, and Sanger sequencing

Genomic DNA was extracted from whole blood. Whole-exome sequencing (WES) or whole-genome sequencing (WGS) was performed at several sequencing centers, including the Genomics Core Facility of the Imagine Institute (Paris, France), the Yale Center for Genome Analysis (USA), the New York Genome Center (NY, USA), the American Genome Center (TAGC, Uniformed Services University of the Health Sciences, Bethesda, USA), and the Genomics Division–Institute of Technology and Renewable Energies (ITER) of the Canarian Health System sequencing hub (Canary Islands, Spain). More technical details are provided in the supplementary materials. For the Sanger sequencing of *OAS1*, *OAS2*, and *RNASEL* variants, the relevant regions of *OAS1*, *OAS2*, and *RNASEL* were amplified by PCR, purified by ultracentrifugation through Sephadex G-50 Superfine resin (Amersham-Pharmacia-Biotech), and sequenced with the Big Dye Terminator Cycle Sequencing Kit on an ABI Prism 3700 apparatus (Applied Biosystems).

### Whole-exome sequencing data analysis

We performed an enrichment analysis focusing on the three candidate genes in our cohort of 558 MIS-C patients and 1288 children and adults with asymptomatic or paucisymptomatic SARS-CoV-2 infection (controls). We considered variants that were predicted to be loss-of-function or missense, with a highest population MAF < 0.01, not included in segmental duplication regions (gnomAD v2.1.1). We considered genes corresponding to the Gene Ontology term “response to virus” (GO:0009615), with a gene damage index of <13.83 (41), corresponding to the 90% least-damaged genes. We searched for all homozygous variants in MIS-C patients, SARS-CoV-2–infected controls, and the gnomAD database. We compared the proportions of patients and controls carrying experimentally confirmed deleterious homozygous variants by means of a logistic regression model, accounting for the ethnic heterogeneity of the cohorts by including the first five principal components of the principal components analysis (PCA), and for data heterogeneity (WGS and WES with various kits and calling processes) by including the two first PCs of a PCA on individual sequence-quality parameters, as previously described (9). The PCA for ethnic heterogeneity was performed with PLINK (v1.9) on WES and WGS data, with the 1000 Genomes Project phase 3 public database as a reference, using >15,000 exonic variants with a MAF > 0.01 and a call rate > 0.99. The PCA for data heterogeneity was performed with the R FactoMineR package and the following individual sequence quality parameters calculated with bcftools stats: number of alleles, number of ALT alleles, number of heterozygous variants, Ts/Tv ratio, number of indels, mean depth of coverage, number of singletons, and number of missing genotypes. We also compared the frequency of experimentally confirmed deleterious homozygous variants of the three genes between our MIS-C cohort and gnomAD using Fisher’s exact test.

### Cell culture

Primary cultures of human fibroblasts were established from skin biopsy specimens from patients or healthy controls. They were transformed with an SV40 vector, as previously described (56), to create immortalized SV40fibroblast cell lines. SV40-fibroblasts, human embryonic kidney 293T (HEK293T) cells, and A549 cells were cultured in Dulbecco’s modified essential medium (DMEM; GIBCO) with 10% fetal bovine serum (FBS) (GIBCO). THP-1 cells were cultured in RPMI 1640 medium (GIBCO) with 10% FBS. For the generation of phorbol-12-myristate-13-acetate (PMA)–primed THP-1–derived macrophages, THP-1 cells were incubated with 50 ng/ml of PMA for 48 hours then left without PMA overnight before stimulation. PBMCs were cultured in RPMI 1640 medium (GIBCO) with 10% FBS. For intracellular poly(I:C) or SARS-CoV-2 stimulation of the PBMCs, blood samples were obtained from the OAS–RNase L–deficient patients 2 months to 1 year after acute-phase MIS-C and from five healthy controls with (two pediatric controls and one adult control) or without (one pediatric control and one adult control) prior asymptomatic or mild SARS-CoV-2 infection ~6 months before sample collection. For the differentiation of monocyte-derived dendritic cells, monocytes were isolated from PBMCs with the Pan Monocyte Isolation kit (Miltenyi Biotec) and cultured with 50 ng/ml of recombinant human granulocyte-macrophage colony-stimulating factor (GM-CSF; PeproTech) and 20 ng/ml of recombinant human IL-13 (PeproTech) for 7 days before cell stimulation experiments.

### Plasmids

For overexpression studies in HEK293T cells, WT cDNAs for *OAS1* and *RNASEL* in a pCMV6 backbone were purchased from Origene. For rRNA degradation assays, human *OAS1* (GenBank accession no. BC071981.1), *OAS2* (GenBank accession no. BC049215.1), *OAS3* (GenBank accession no. BC113746), and *RNASEL* (GenBank accession no. L10381.1) cDNAs were inserted into p3X-FLAG-CMV-10 (Sigma) as previously described (75, 88). Patient-specific variants or variants from the gnomAD database were generated by site-directed mutagenesis PCR with the Super Pfx DNA Polymerase (CWbio). For stable lentivirus-mediated transduction with *ACE2* and *RNASEL*, cDNAs for WT and patient-specific *ACE2* or *RNASEL* variants were inserted into pTRIP-SFFV-CD271-P2A, a modified pTRIP-SFFV-mtagBFP-2A (Addgene 102585) in which mtagBFP is replaced with CD271, with InFusion (Takara Bio), according to the manufacturer’s instructions. We used the XhoI and BamHI restriction sites. For stable lentivirus-mediated transduction with *OAS1* and *OAS2*, cDNAs for WT and patient-specific *OAS1* or *OAS2* variants were inserted into a modified pSCRPSY vector (KT368137.1) with a PaqCI cutting site expressing blue fluorescent protein (BFP). The PaqCI site was used for cDNA insertion with InFusion. We checked the entire sequences of the *OAS1*, *OAS2*, OAS3, and *RNASEL* cDNAs in the plasmids by Sanger sequencing.

### Cell-free system assays of OAS and RNase L activity

Assays for OAS and RNase L activity were performed with a modified cell-free system assay based on HeLa M cells (49, 50). The HeLa M cells were cultured in DMEM with 10% FBS, and their identity was confirmed by the presence of short tandem repeat loci with a 94.12% match to HeLa cells (ATCC CCL2, Genetica, Burlington, NC). We previously reported that HeLa M cells have no RNase L expression (51). Cells were plated in 24-well dishes (6 × 10^4^ cells per well) with empty vector (p3X-FLAG-CMV-10) or vector containing WT or mutant human *OAS1* (GenBank accession no. BC071981.1), *OAS2* (GenBank accession no. BC049215.1), *OAS3* (GenBank accession no. BC113746), or *RNASEL* (GenBank accession no. L10381.1) cDNAs. HeLa M cells were cotransfected with cDNAs in the presence of Lipofectamine 2000 for 20 hours. Conditions were optimized for each type of enzyme assayed. RNase L assays were performed on cells cotransfected with 300 ng of WT or mutant *RNASEL* cDNA and 100 ng of WT *OAS3* cDNA. *OAS1* assays were performed with 300 ng of *OAS1* cDNA and 100 ng of *RNASEL* cDNA. OAS2 assays were performed with 300 ng (condition1) or 600 ng (condition2) of *OAS2* cDNA and 100 ng of *RNASEL* cDNA, and *OAS3* assays were performed with 300 ng of OAS3 cDNA and 100 ng of *RNASEL* cDNA. The lysis-activation-reaction (LAR) buffer contained 0.1% (by volume) Nonidet P-40, 50 mM Tris-HCl pH 7.5, 0.15 M NaCl, 2 mM EDTA, 10 mM MgCl_2_, 2 mM ATP, 400 U/ml of RNaseOUT (Thermo Fisher Scientific), and 2.5 μg/ml of poly(I): poly(C) (Millipore catalog no. 528906). LAR buffer (75 μl) was added to each well of cells on ice and the contents of the wells were then transferred to tubes on ice. The lysates were then incubated at 30°C for 30 min, except in OAS2 assays, for which lysates were incubated at 37°C (condition 1) or 30°C (condition 2) for 40 and 50 min, respectively. Total RNA was isolated with RLT buffer supplemented with guanidinium isothiocyanate and the EZ-10 Spin Columns Total RNA Minipreps Super kit (BIO BASIC). RNA was separated on RNA chips with an Agilent Bioanalyzer 2000, from which images and RNA integrity numbers (RINs) were obtained. For immunoblots, aliquots of the lysates (10 μg of protein) were separated by SDS–polyacrylamide gel electrophoresis (SDS-PAGE) in a 7% acrylamide gel. Immunoblots were probed with a monoclonal antibody against the Flag epitope or β-actin (Sigma-Aldrich).

### FRET-based OAS enzyme assays

FRET assays of the amount of 2–5A synthesized by WT and mutant isoforms of OAS1 or OAS2 were performed with lysates of transfected HeLa M cells (90). Cells were plated in 24-well dishes (6 × 10^4^ cells per well), cultured for 24 hours and transfected for 20 hours with Lipofectamine 2000 transfection reagent (Thermo Fisher Scientific) and 0.5 μg empty vector (p3X-FLAG-CMV-10), or 500 ng of vector containing WT or mutant OAS1 or OAS2. Cells were washed with cold PBS and then lysed with 100 μl of LAR buffer [containing ATP and poly(I:C)] per well on ice. The lysates were transferred to tubes on ice and incubated at 30°C for 50 min before heating at 95°C for 10 min (to stop the reaction and denature proteins) and vortexing twice. The lysates were centrifuged at 12,000g for 10 min. The supernatants were then collected and diluted 10-fold in H_2_O. Diluted samples (2 μl) were added to 45 μl of cleavage buffer (25 mMTris-HCl,pH7.4,0.1MKCl,10mMMgCl_2_, 50 μM ATP pH 7.4, and 7 mM β-mercaptoethanol) containing 40 nM RNase L and 135 nM FRET probe in 96-well plates. The probe used was a 36-nucleotide synthetic oligoribonucleotide probe with multiple RNase L cleavage sites, a fluorophore (6-FAM or 6-carboxyfluorescein) atthe5′ terminus, andthe black holequencher-1 (BHQ1) at the 3′ terminus (IDT, Inc.) (90). FRET assays were performed at room temperature, every 5 min, for 30 min. Fluorescence was measured in relative fluorescence units (RFU), with excitation at 485 nm and emission at 535 nm, with a Varioskan LUX multimode microplate reader and Skanit version 6.0.1 software (Thermo Fisher Scientific). There were six biological replicates for each treatment group. Standard curves were plotted in triplicate with 0.1 to 30 nM ppp5′A2′p5′A2′p5′A (trimer 2–5A) synthesized with isolated OAS1 and purified by high-performance liquid chromatography (HPLC) (70).

### Cytokine quantification in plasma samples

Cytokine quantification in plasma samples was performed as previously described (32). Briefly, whole blood was sampled into EDTA tubes. The plasma concentrations of IFN-γ, IL-1RA, IL-10, IL-18, IL-6, MCP-1, soluble CD25, and TNF were then determined with Simpleplex technology and an ELLA instrument (Protein Simple) according to the manufacturer’s instructions. Plasma IFN-α concentrations were determined with a single-molecule array (Simoa) on an HD-1 Analyzer (Quanterix) with a commercial kit for IFN-α2 quantification (Quanterix). Blood samples from P1, P2, and P5 were obtained on days 7, 4, and 9 after symptom onset, respectively.

### TRBV 11–2 *relative expression levels*

Whole blood was collected into PAXgene (BD Biosciences) or Tempus (Thermo Fisher Scientific) blood RNA tubes or EDTA tubes. RNA was extracted with the corresponding RNA extraction kits or with the Maxwell 16 LEV Blood RNA kit and a Maxwell extractor (Promega) and quantified by spectrometry (Nanovue). For P5, RNA was extracted from sorted T cells with the RNeasy Plus microkit (Qiagen). Relative expression levels were determined for *TRBV 11–2* with nCounter analysis technology (NanoString Technologies), by calculating *TRBV 11–2* mRNA levels relative to other *TRBV* mRNA levels and normalizing against the median value for the healthy volunteer group. Blood samples from P1, P2, and P5 were obtained on days 7, 4, and 9 after symptom onset, respectively.

### Immunoblots

Total protein extracts were prepared by lysing cells in NP40 lysis buffer (150 mM NaCl, 50 mM Tris pH 8.0, and 1.0% NP40) supplemented with cOmplete Protease Inhibitor cocktail (Roche, Mannheim, Germany). Equal amounts of protein from each sample were subjected to SDS-PAGE, and the proteins were blotted onto polyvinylidene difluoride membranes (BioRad). The membranes were then probed with the desired primary antibody followed by the appropriate secondary antibody. Primary antibodies against the following targets were used: Flag tag (Sigma-Aldrich, cat: F1804), human OAS1 (Cell Signaling, cat: 14498), OAS2 (Proteintech, cat: 19279–1-AP), RNase L (Cell Signaling, cat: 27281), RIG-I (Cell Signaling, cat: 3743), MDA5 (Cell Signaling, cat: 5321), MAVS (Cell Signaling, cat: 3993), phospho-IRF3 (Cell Signaling, cat: 4947), total IRF3 (Cell Signaling, cat: 11904), phospho-p65 (Cell Signaling, cat: 3033), and total p65 (Santa Cruz, cat: sc-372). Membranes were probed with a horseradish peroxidase (HRP)–conjugated antibody against GAPDH (Proteintech, cat: HRP-60004), as a protein loading control. Antibody binding was detected by enhanced chemiluminescence (Thermo Fisher Scientific).

### RT-qPCR

Total RNA was extracted from THP-1 cells and various other cell types with the Quick-RNA MicroPrep kit (Zymo Research). RNA was reverse-transcribed with random hexamers and the Superscript III first-strand cDNA synthesis system (Invitrogen). Quantitative real-time PCR was then performed with the TaqMan universal PCR master mix (Applied Biosystems). For gene expression assays, TaqMan probes for *OAS1*, *OAS2*, *OAS3*, *RNASEL*, *IL6*, and *CXCL9* were used (Thermo Fisher Scientific). We used β-glucuronidase (*GUSB*) for normalization (Applied Biosystems). The results were analyzed with the ΔCt or ΔΔCt method. For SARS-CoV-2 genomic RNA quantification, RNA was extracted from 3 × 10^5^ THP-1 cells infected with SARS-CoV-2 for 24 hours. Cells were washed three times with PBS and lysed for RNA extraction. Equal amounts of total RNA were reverse-transcribed with random hexamers and the Superscript III first-strand cDNA synthesis kit (Invitrogen). Equal amounts of cDNA were used for the qPCR reaction. Primers and probes for the N gene (N2 region), the RNA-dependent RNA polymerase (RdRP) gene, and their respective standards were purchased from IDT technologies. All qPCR reactions were analyzed with the QuantStudio 3 system.

### Gene knockout

OAS1 knockout THP-1 cells and the parental WT cells were kindly provided by W.-B. Lee (62). The THP-1 cells with knockouts for RIG-I, MDA5, and MAVS were purchased from Invivogen. A549 KO cells were kindly provided by S. Weiss (55). For the generation of OAS2 and RNase L KO THP-1 cells, a set of three single-guide RNAs for *OAS2* or *RNA-SEL* (Synthego) were combined with True-Cut Cas9 protein v2 (Invitrogen) and used for the nucleofection of the cells with Cell Line Nucleofection kit V (Lonza) and AMAXA Nucleofector 2b (Lonza), according to the manufacturer’s instructions. The cells were cultured for several days and then plated at clonal density in *96*-well plates and amplified. Genomic DNA was extracted from multiple clones, and genomic regions of ~450 bp around the *OAS2* or *RNASE*L single guide RNAs were subjected to Sanger sequencing. The absence of the protein was confirmed by immunoblotting. The loss of RNase L activity in RNase L KO THP-1 cells was confirmed in an rRNA degradation assay. The sequences of the guide RNAs for OAS2 and RNase L knockouts were 5′-AGCUGAGAGCAAUGGGAAAU-3′, 5′-UCAGACACUGAUCGACGAGA-3′, and 5′-UGCACCAGGGGGAACUGUUC-3′ (*OAS2*); and 5′-GCAGUGGAGAAGAAGCACUU-3′, 5′-GCAGGUGGCAUUUACCGUCA-3′, and 5′-UUUGACCUUACCAUACACAG-3′ (*RNASEL*). The sequencing primers were 5′-CAGTTTCAGTTTCCTGGCTCTGG-3′ and 5′-GCACATAATAGGCACCCAGCAC-3′ for *OAS2* and 5′-CTCTGTTGCCAGAGAATCCCAATTTAC-3′, 5′-CAATCGCTGCGAGGATAAAAGG-3′, 5′-GAGCGTGAAGCTGCTGAAAC-3′, and 5′-TGTACTGGCTCCACGTTTG-3′ for *RNASEL*.

### Gene knockdown

The shRNA-mediated silencing experiments were performed with GIPZ (Horizon Discovery) lentiviral vectors encoding microRNA-adapted shRNAs targeting the open reading frame of *OAS1* (catalog nos. 200201641 and 200293786), *OAS2* (200260991 and 200255637), and *RNASEL* (200226261 and 200226578), or a nonsilencing control shRNA (RHS4346). Lentiviral particles encoding shRNA were generated by the transient transfection of HEK293T cells with lentiviral GIPZ vectors and a mixture of packaging plasmids with X-tremeGENE 9 transfection reagent, used according to the manufacturer’s instructions. Briefly, HEK293T cells at 80 to 90% confluence in a six-well plate were transfected with 1.5 μg of the lentiviral vector GIPZ, 1μg of the packaging plasmid (psPAX2, Addgene), and 0.5 μg of the envelope plasmid (pMD2G, Addgene). The medium was changed the following day, and the virus-containing supernatant was collected 48 hours after transfection, passed through a filter with 0.45-μm pores, and used directly for cell transduction or stored at −80°C.

For the transduction of THP-1 cells, the cells were incubated with supernatants containing the lentiviral particles. The medium was replaced with fresh medium the following day, and puromycin was added 3 days after transduction, to a final concentration of 2 μg/ml. Protein production was analyzed by immunoblotting after 4 days of selection. All the experiments were performed between days 7 and 14 after transduction.

For shRNA-mediated knockdown experiments in primary monocyte-derived dendritic cells (MDDCs), a high transduction efficiency (>60% GFP^+^ cells) was achieved by cotransduction with shRNA-encoding lentiviral particles and virion-like particles (VLPs) carrying the SIV viral protein Vpx (VLP-Vpx). Vpx suppresses the SAMHD1-mediated restriction of lentiviral reverse transcription in myeloid cells. VLP-Vpx were produced by transfecting HEK293T cells with 1.5 mg of the packaging vector SIV3+ (derived from SIVmac251) and 0.5 μg of the envelope plasmid pMD2G with XtremeGENE9. Monocytes were isolated from PBMCs from healthy donors by negative selection with the Pan Monocyte Isolation Kit (Miltenyi Biotec). Freshly purified monocytes were transduced with shRNA-encoding lentiviral particles and VLP-Vpx in the presence of protamine (8 μg/ml). Transduced cells were allowed to differentiate into MDDCs in the presence of recombinant human GM-CSF (10 ng/ml) and IL-4 (25 ng/ml) for 5 days.

### Lentiviral transduction

HEK293T cells were dispensed into a six-well plate at a density of 8 × 10^5^ cells per well. The next day, cells were transfected with pCMV-VSV-G (0.2mg), pHXB2-env (0.2μg; NIH-AIDS Reagent Program; 1069), psPAX2 (1μg; Addgene plasmid no. 12260), and either pTRIP-SFFV-CD271-P2A empty vector or encoding the protein of interest (1.6μg) in Opti-MEM (Gibco; 300μl) containing X-tremeGENE 9 (Sigma Aldrich; 10μl), according to the manufacturer’s instructions. After 6 hours, the medium was replaced with 3 ml of fresh culture medium, and the cells were incubated for a further 24 hours for lentiviral particle production. The viral supernatant was collected and passed through a syringe filter with 0.2-μm pores (Pall) to remove debris. Protamine sulfate (Sigma; 10μg/ml) was added to the supernatant, which was then used immediately or stored at −80°C until use.

For the transduction of THP-1 cells with *OAS1*, *OAS2*, or *RNASEL*, the corresponding gene KO THP-1 cells were dispensed into a 12-well plate at a density of 1 × 10^6^ cells per well, in 500μl of culture medium per well. Viral supernatant was then added (500 μl per well) the next day. For the transduction of SV40-fibroblasts with ACE2, healthy control or patient-specific SV40-fibroblasts were used to seed six-well plates at a density of 5 × 10^5^ cells per well. Viral supernatant was added (500 μl per well) the next day. The cells were then incubated for a further 48 hours at 37°C. Transduction efficiency was evaluated by surface staining for CD271 (Miltenyi Biotec) for the pTRIP vector, or by flow cytometry to evaluate BFP expression levels for the pSCRPSY vector. MACS column separation was performed with selection beads for CD271-positive cells (Miltenyi Biotec) if the proportion of CD271-positive cells was <80%. Cells transduced with the pSCRPSY vector were selected with puromycin or by flow cytometry. Protein production was subsequently validated by immunoblotting.

### SARS-CoV-2 infection

The SARS-CoV-2 NYC isolate was obtained from the saliva of a deidentified patient on 28 July 2020. The sequence of the virus is publicly available (GenBank OM345241). The virus isolate was initially amplified in Caco-2 cells (passage 1, or P#1 stock). For the generation of P#2 and P#3 working stocks, Caco-2 cells were infected with the P#1 and P#2 viruses, respectively, at a multiplicity of infection (MOI) of 0.05 plaque-forming units (PFU)/cell and incubated for 6 and 7 days, respectively, at 37°C. The virus-containing supernatant was then harvested, clarified by centrifugation (3000g for 10 min), and filtered through a disposable vacuum filter system with 0.22-μm pores. The P#3 stock used in this study had a titer of 3.4 × 10^6^ PFU/ml determined on Vero E6 cells with a 1% methylcellulose overlay, as previously described (72).

A549 + ACE2/TMPSS2 cells, human SV40-fibroblasts + ACE2, or THP-1 cells were used to seed 96-well plates at a density of 1.5 × 10^4^ cells per well, 4 × 10^3^ cells per well, and 1 × 10^5^ cells per well, respectively, in the presence or absence of IFN-α2b at a concentration of 1000 IU/ml. The cells were infected with SARS-CoV-2 24 hours later by directly adding 10 μl of virus stock at various dilutions to the wells (final volume: 110 μl). Cells were infected for 24, 48, or 72 hours. The cells were fixed with neutral buffered formalin at a final concentration of 10% and stained for SARS-CoV-2 with an anti-N antibody (catalog no. GTX135357; GeneTex). An Alexa Fluor 488- or Alexa Fluor 647-conjugated secondary antibody (Invitrogen) was used. Plates were imaged with an ImageXpress micro XL and analyzed with MetaXpress (Molecular Devices).

### Cell stimulation

THP-1 cells were used to coat a 96-well plate at a density of 1 × 10^5^ cells per 100 μl of culture medium. For stimulations of PBMCs and MDDCs, we used 1 × 10^5^ cells and 5 × 10^5^ cells per 100 μl of culture medium, respectively. The cells were stimulated with the indicated stimulus at the specified concentrations, with or without lipofectamine 2000 (Invitrogen), according to the manufacturer’s instructions. Poly(I:C), 5′ppp-dsRNA, 5′ppp-dsRNA control, ISD, ISD control, R848, CPG-ODN2006, and LPS were purchased from Invivogen. For exogenous 2′5′-linked oligoadenylate (2–5A) or dephosphorylated 2–5A, we used 20 μM of 2–5A for transfection in the presence of lipofectamine simultaneously with the other stimuli [poly(I:C), R848, or LPS]. Dephosphorylated 2–5A (A2′p5′A2′p5′A) was prepared by treating 2–5A with shrimp alkaline phosphatase (SAP) (Thermo Fisher Science) to remove the 5′-triphosphoryl group from 2–5A, rendering it unable to activate RNase L (69, 70). The dephosphorylation reaction mixture contained 5 mM 2–5A incubated with five units of SAP at 37°C for 1 hour, according to the manufacturer’s protocol. Samples were denatured by incubation at 95°C for 5 min. Supernatants containing dephosphorylated 2′,5′-A3 were removed after centrifugation at 18,000g for 15 min at 4°C. Dephosphorylated 2–5A was then validated by HPLC and FRET assays for RNase L activity. After cell stimulation, the cells or supernatants were harvested, and their cytokine mRNA and protein levels were assessed by RT-qPCR and with a multiplex bead assay (BioLegend), respectively.

### Detection of secreted cytokines in a multiplex bead assay

The harvested supernatants of stimulated THP-1 cells, PBMCs, and other types of cells were prepared and used for the LEGENDplex multiplex bead assay (BioLegend), according to the manufacturer’s instructions. Samples were analyzed by flow cytometry on an Attune NxT flow cytometer, according to the manufacturer’s instructions. Data were analyzed with LEGENDplex Cloud-based Data Analysis Software.

### Luciferase assay

THP-1 cells expressing an ISRE-lucia luciferase reporter gene were purchased from Invivogen (THP1-Dual). Cells were stimulated according to the conditions specified above. The supernatant was collected and used for the luciferase assay in accordance with the manufacturer’s instructions.

### Coculture of THP-1 and SARS-CoV-2–infected cells

Vero cells were plated in a six-well plate and infected at a MOI of 0.05 (as determined by plaque assay on Vero E6 cells) for a total of 48 hours. The supernatant of the infected cells was carefully removed, and the infected cells were then transferred to fresh THP-1 culture medium. A fixed volume of the resulting cell suspension was then dispensed onto WT or RNase L KO THP-1 cells plated in a 96-well plate at a density of 1 × 10^5^ cells in 100 μl. THP-1 cells stimulated with SARS-CoV-2 only were stimulated in parallel for 24 hours. THP-1 cells were stimulated for a total of 24 hours before collection of the supernatant for cytokine determinations and cells for total RNA extraction.

### Transfection of THP-1 cells with RNA from SARS-CoV-2–infected cells

Total RNA was extracted from mock-infected Vero cells or Vero cells infected with SARS-CoV-2 at a MOI of 0.05 for a total of 72 hours. THP-1 cells were transfected with 2 μg/ml of total RNA extract for 8 hours. THP-1 cells were then collected for total RNA extraction.

### Deep immunophenotyping by mass cytometry (CyTOF)

CyTOF was performed on whole blood with the Maxpar Direct Immune Profiling Assay (Fluidigm), according to the manufacturer’s instructions, as previously described (7). Cells were frozen at −80°C after overnight staining to eliminate dead cells, and acquisition was performed on a Helios machine (Fluidigm). The antibodies used for staining are listed in table S3. All the samples were processed within 24 hours of sampling. Data analysis was performed with OMIQ software.

### Bulk RNA sequencing (RNA-seq)

Total RNA was extracted from THP-1 cells or sorted blood cell populations. Cells were left untreated or were stimulated with poly(I:C) in the presence of lipofectamine or infected with SARS-CoV-2. RNA was extracted with the Quick-RNA MicroPrep kit (Zymo Research) or the RNeasy Micro Kit (Qiagen) and treated with DNase I (Zymo Research and Qiagen) to remove residual genomic DNA. RNA-seq libraries were prepared with the Illumina RiboZero TruSeq Stranded Total RNA Library Prep Kit (Illumina) and sequenced on the Illumina NovaSeq platform in the 100 nucleotide, pairedend configuration. Each library was sequenced twice.

The RNA-seq FASTQ files were first inspected with fastqc to ensure that the raw data were of high quality. The sequencing reads of each FASTQ file were then aligned with the GENCODE human reference genome GRCh37.p13 with STAR aligner v2.6 and the alignment quality of each BAM file was evaluated with RSeQC. Reads were quantified with featureCounts v1.6.0 to generate gene-level feature counts from the read alignment, based on GENCODE GRCh37.p13 gene annotation. The gene-level feature counts were then normalized and log_2_-transformed with DESeq2, to obtain gene expression values for all genes and all samples. Differential gene expression analyses were conducted by contrasting the intracellular poly(I:C)-stimulated samples or the SARS-CoV-2–infected samples with the nonstimulated samples. For each gene expression analysis, we performed trimmed mean of M values (TMM) normalization and gene-wise generalized linear model regression by edgeR, and the genes displaying significant differential expression were selected according to the following criteria: FDR ≤ 0.05 and |log2(FoldChange)| ≥ 1. Differential gene expression was plotted as a heatmap with ComplexHeatmap, and genes and samples were clustered according to complete linkage and the Euclidean distances of gene expression values. GSEA was conducted with the fgsea package, by projecting the ranking of fold-change in expression onto the Hallmark gene sets (71).

### Single-cell RNA sequencing of PBMCs

We performed scRNA-seq on SARS-CoV-2– and mock-stimulated PBMCs sampled from four individuals with inborn errors of the OAS–RNaseL pathway (P1 with OAS1 deficiency, P2 and P3 with OAS2 deficiency, P5 with RNase L deficiency), three individuals with inborn errors of type I IFN immunity, and eight healthy donors—one pediatric control and one adult control with a history of past asymptomatic SARS-CoV-2 infection, and two pediatric controls and four adult controls with no history of prior SARS-CoV-2 infection. The cryopreserved PBMCs were thawed, stimulated, and processed for scRNA-seq. Across all samples, we captured 46,157 high-quality single-cell transcriptomes that were classified into five major immune cell lineages: myeloid, B, CD4^+^ T, CD8^+^ T, and NK cells. The data were then analyzed as described in detail in the supplementary materials.

We also performed scRNA-seq on cryopreserved PBMCs from P5 (RNase L–deficient, aged 4 years) sampled during the acute (9 days after MIS-C onset) and convalescent (~1 month after onset) phases, together with cells from one healthy adult and two pediatric controls. We compared the data obtained with a previously published dataset for patients with pediatric acute SARS-CoV-2 infection or MIS-C (33). Clustering analysis showed lower levels of monocytes and type 1 and type 2 conventional dendritic cells (cDCs) in these patients and an expansion of the activated T cell population strongly expressing *MKI67* (Fig. 5F and fig. S8, A and B). Other subsets were largely unaffected. Pseudobulk differential expression analysis was performed at the single-cell level for monocytes, mDCs, B lymphocytes, plasmacytoid dendritic cells (pDCs), and activated T cells. Bulk RNA-seq was performed on sorted nonclassical monocytes and pDCs to further confirm the scRNA-seq findings. We also quantitatively inferred cell–cell communications with Cell-Chat (74) to identify the signal-outgoing and the signal-receiving cell subsets. The data generated during this study were analyzed in an integrative manner with historical controls from the laboratory (one pediatric and seven adult controls), publicly available control PBMC datasets downloaded from the 10X Genomics web portal (https://support.10xgenomics.com/single-cell-gene-expression/datasets), and a previously published dataset for patients with acute SARS-CoV-2 infection and MIS-C (GEO accession: GSE167029), as described in detail in the supplementary materials. In addition, two other previously published sets of scRNA-seq data for pediatric healthy controls and children with acute SARS-CoV-2 infection or MIS-C (GSE166489) (91) were used for an independent cohort analysis.

### Statistical analysis

For experiments performed in vitro, quantitative data were obtained for cells carrying the different mutations and control cells, or cells treated with different stimuli, from at least three biological replicates. For each biological replicate, up to six technical replicates were performed and averaged for downstream analysis. Cytokine determinations were log-transformed after subtracting the limit of detection for the experiment concerned. Mean quantitative values were compared between cells carrying the various mutations and control cells or cells treated with different stimuli in unequal-variance *t* tests. Where relevant, statistical test results are indicated in the corresponding figures (ns, not significant; *P < 0.05, **P < 0.01, ***P < 0.001, ****P < 0.0001).

## Supplementary Material

Data S2

Data S1

Mdar_reproducibility_checklist

Supplemental material

Data_captions

## Figures and Tables

**Table 1.** Homozygous or potentially compound-heterozygous rare nonsynonymous variants of the *OAS* and *RNASEL* genes in MIS-C patients. Homozygous or potentially compound-heterozygous nonsynonymous variants with a minor allele frequency (MAF) < 0.01 (gnomAD) found in our cohort of MIS-C patients. CADD_Phred, combined annotation-dependent depletion Phred score; Exp function, experimental function of each variant as tested in the RNase L–dependent rRNA degradation assay (OAS1, OAS2, RNase L) and FRET assay (OAS1, OAS2); Hom, homozygous; Het, heterozygous.

Gene	Nucleotide change	Amino acid change	Zygosity	MAF (gnomAD)	CADD_Phred	Exp function
*OAS1*	c.139C>T	p.Arg47* (R47*)	Hom	0.00017327	36	LOF
*OAS2*	c.1604G>A	p.Arg535Gln (R535Q)	Hom	0.00028695	13.58	Hypomorph
*OAS2*	c.773A>T	p.Gln258Leu (Q258L)	Het	–	3.888	Hypomorph
*OAS2*	c.868G>A	p.Val290Ile (V290I)	Het	0.0005153	5.585	Hypomorph
*OAS3*	c.145G>A	p.Ala49Thr (A49T)	Het	0.00243639	9.48	Isomorph
*OAS3*	c.1475G>A	p.Arg492His (R492H)	Het	0.0054987	9.95	Isomorph
*OAS3*	c.1703G>A	p.Arg568Lys (R568K)	Het	0.00104951	0.472	Isomorph
*OAS3*	c.2795G>A	p.Arg932Gln (R932Q)	Het	0.0094	23.2	LOF
*OAS3*	c.3089A>G	p.Gln1030Arg (Q1030R)	Het	–	23.9	Isomorph
*OAS3*	c.1586A>G	p.Gln529Arg (Q529R)	Het	0.00000401	5.85	Isomorph
*OAS3*	c.792C>A	p.His264Gln (H264Q)	Het	0.001001261	0.924	Isomorph
*OAS3*	c.442C>T	p.Pro148Ser (P148S)	Het	0.000036	22.9	Isomorph
*OAS3*	c.3259G>A	p.Val1087Met (V1087M)	Het	0.003936537	22.5	Isomorph
*RNASEL*	c.790A>G	p.Ile264Val (I264V)	Hom	0.00000401	6.597	Isomorph
*RNASEL*	c.793G>T	p.Glu265* (E265*)^†^	Hom	0.0031	33	LOF
*RNASEL*	c.175G>A	p.Gly59Ser (G59S)^†^	Hom	0.0031	22.9	Isomorph

† *RNASEL* variants p.E265* and p.G59S were in complete linkage disequilibrium (https://www.internationalgenome.org), forming a haplotype.

**Table 2.** Demographic and clinical information for MIS-C patients biallelic for deleterious variants of the OAS–RNase L pathway. IEI, inborn error of immunity; SCV2, SARS-CoV-2; IVIG, intravenous immunoglobulins; ND, not determined; CRP, C-reactive protein; sCD25, soluble IL-2Rα.

Patient	P1	P2	P3	P4	P5
IEI (inheritance mode)	*OAS1* (AR)	*OAS2* (AR)	*OAS2* (AR)	*OAS2* (AR)	*RNASEL* (AR)
Age at MIS-C diagnosis	3 months	3 years	14 years	9 years	4 years
Sex	Male	Male	Female	Female	Female
Ethnicity	Filipino	Spanish	Turkish	Turkish	French Canadian
Resident country	Spain	Spain	Turkey	Turkey	Canada
SCV2 virology	Nasal swab PCR (−); blood PCR (−); blood anti-SCV2 IgG (+); blood antigen N (−)	Nasal swab PCR (−); blood PCR (−); blood anti-SCV2 IgG (+); blood antigen N (−)	Nasal swab PCR (−); blood PCR (ND); blood total anti-SCV2 (+); blood antigen N (ND)	Nasal swab PCR (−); blood PCR (ND); blood anti-SCV2 IgM and IgG (+); blood antigen N (ND)	Nasal swab PCR (−); blood PCR (−); blood anti-SCV2 IgG (+); blood antigen N (−)
Hemogram	Normal	Normal	Normal	Normal	Normal
Increased markers of multiorgan inflammation	CRP, ferritin, pro-BNP, GM-CSF, IL-1RA, MCP1, sCD25, IL-18, TNF	CRP, ferritin, pro-BNP, MCP1, sCD25, IL-1RA, IL-18, TNF	CRP, ferritin, troponin	Ferritin, troponin, pro-BNP	sCD25
*TRBV 11-2* expansion	(−)	(−)	ND	ND	(+)
Clinical presentation	Kawasaki-like disease: fever, gastrointestinal symptoms, hepatosplenomegaly, aseptic meningitis with neurological symptoms (irritability), peripheral edema, lymphadenopathy, bilateral coronary aneurysm (Z score +8, +8.7), possible cerebral arterial aneurysm	Kawasaki disease: fever, rash, bilateral eyelid edema and erythema, conjunctival hyperemia	Kawasaki-like disease: fever, rash, bilateral nonpurulent conjunctivitis, strawberry tongue, abdominal pain, vomiting, dyspnea, mild mitral insufficiency. One and a half months prior, the patient had fever, headache, and sore throat when her mother had COVID-19. The patient developed oligoarticular juvenile idiopathic arthritis 5 months after MIS-C.	Fever, vomiting, coughing, myocarditis, left ventricular failure, pulmonary edema with paracardiac infiltration, polyneuropathy	Kawasaki disease: fever, rash, erythema and edema of the feet, anterior uveitis, cervical lymphadenopathy
Treatment	IVIG, aspirin, corticosteroids, anticoagulation therapy	IVIG, aspirin	IVIG, methylprednisolone, heparin	IVIG, pulse steroid, anakinra, mechanical ventilation	IVIG
Outcome	Recovery	Recovery	Recovery, but with persistent arthralgia in both knees 1.5 years after MIS-C	Recovery	Recovery

## Data Availability

All data are available in the manuscript or the supplementary materials. The materials and reagents used are commercially available and nonproprietary, with the exception of SARS-CoV-2 working stock and the gene-KO or patient-specific cell lines generated from this study. The SARS-CoV-2 working stock is available from C.M.R. under a material transfer agreement (MTA) with the Rockefeller University. The cell lines generated from this study are available from S.-Y.Z. and J.-L.C. upon request under MTAs from the Rockefeller University and the Imagine Institute. Patient-specific cellular materials from patients enrolled at NIAID are available from H.C.S. under a MTA with the NIH, provided that the request fulfills all articles listed in a MTA with the originating institute where the materials were collected. WGS data for patients sequenced by NIAID through TAGC were deposited under database of Genotypes and Phenotypes (dbGaP) accession number phs002245. Other genomic sequences of the patients reported in this paper are available from the authors upon request under a data transfer agreement. The raw RNA-seq data generated from this study are deposited in the NCBI database under the NCBI-SRA project PRJNA898284.

## CoV-Contact Cohort

Loubna Alavoine^1^, Sylvie Behillil^2^, Charles Burdet^3^, Charlotte Charpentier^3,4^, Aline Dechanet^5^, Diane Descamps^3,6^, Xavier Duval^1,3,7^, Jean-Luc Ecobichon^1^, Vincent Enouf^8^, Wahiba Frezouls^1^, Nadhira Houhou^5^, Ouifiya Kafif^5^, Jonathan Lehacaut^1^, Sophie Letrou^1^, Bruno Lina^9^, Jean-Christophe Lucet^10^, Pauline Manchon^5^, Mariama Nouroudine^1^, Valentine Piquard^5^, Caroline Quintin^1^, Michael Thy^11^, Sarah Tubiana^1^, Sylvie van der Werf^8^, Valérie Vignali^1^, Benoit Visseaux^3,10^, Yazdan Yazdanpanah^3,10^, Abir Chahine^12^, Nawal Waucquier^12^, Maria-Claire Migaud^12^, Dominique Deplanque^12^, Félix Djossou^13^, Mayka Mergeay-Fabre^14^, Aude Lucarelli^15^, Magalie Demar^13^, Léa Bruneau^16^, Patrick Gérardin^17^, Adrien Maillot^16^, Christine Payet^18^, Bruno Laviolle^19^, Fabrice Laine^19^, Christophe Paris^19^, Mireille Desille-Dugast^19^, Julie Fouchard^19^, Denis Malvy^20^, Duc Nguyen^20^, Thierry Pistone^20^, Pauline Perreau^20^, Valérie Gissot^21^, Carole Le Goas^21^, Samatha Montagne^22^, Lucie Richard^23^, Catherine Chirouze^24^, Kévin Bouiller^24^, Maxime Desmarets^25^, Alexandre Meunier^26^, Benjamin Lefèvre^27^, Hélène Jeulin^28^, Karine Legrand^29^, Sandra Lomazzi^30^, Bernard Tardy^31^, Amandine Gagneux-Brunon^32^, Frédérique Bertholon^33^, Elisabeth Botelho-Nevers^32^, Kouakam Christelle^34^, Leturque Nicolas^34^, Layidé Roufai^34^, Karine Amat^35^, Sandrine Couffin-Cadiergues^34^, Hélène Espérou^36^, Samia Hendou^34^

^1^Centre d’Investigation Clinique, INSERM CIC 1425, HôpitalBichat Claude Bernard, AP-HP, Paris, France. ^2^Institut Pasteur, Paris, France. ^3^Université de Paris, IAME, INSERM U1137, Paris, France, Hôpital Bichat Claude Bernard, AP-HP, Paris, France. ^4^Service de Virologie, Université de Paris, INSERM, IAME, UMR 1137, AP-HP, Hôpital Bichat Claude Bernard, F-75018 Paris, France. ^5^Hôpital Bichat Claude Bernard, AP-HP, Paris, France. ^6^IAME INSERM U1140, Hôpital Bichat Claude Bernard, AP-HP, Paris, France. ^7^Centre d’Investigation Clinique, INSERM CIC 1425, AP-HP, IAME, Paris University, Paris, France. ^8^Institut Pasteur, U3569 CNRS, Université de Paris, Paris, France. ^9^Virpath Laboratory, International Center of Research in Infectiology, Lyon University, INSERM U1111, CNRS U5308, ENS,UCBL, Lyon, France. ^10^IAME INSERM U1138, Hôpital Bichat Claude Bernard, AP-HP, Paris, France. ^11^Center for Clinical Investigation, AP-HP, Hôpital Bichat Claude Bernard, Paris, France. ^12^Centre d’Investigation Clinique, INSERM CIC 1403, Centre Hospitalo Universitaire de Lille, Lille, France. ^13^Service des maladies infectieuses, Centre Hospitalo Universitaire de Cayenne, Guyane, France. ^14^Centre d’Investigation Clinique, INSERM CIC 1424, Centre Hospitalier de Cayenne, Cayenne, Guyane Française. ^15^Service Hôpital de jour Adulte, Centre Hospitalier de Cayenne, Guyane, France. ^16^Centre d’Investigation Clinique, INSERM CIC 1410, Centre Hospitalo universitaire de la Réunion, La Réunion, France. ^17^Centre d’Investigation Clinique, INSERM CIC 1410, CHU Reunion, Saint-Pierre, Reunion Island. ^18^Centre d’Investigation Clinique, INSERM CIC 1410, Centre de Ressources Biologiques, Centre Hospitalo universitaire de la Réunion, La Réunion, France. ^19^Centre d’Investigation Clinique, INSERM CIC 1414, Centre Hospitalo universitaire de Rennes, Rennes, France. ^20^Service des maladies infectieuses, Centre Hospitalo universitaire de Bordeaux, Bordeaux, France. ^21^Centre d’Investigation Clinique, INSERM CIC 1415, CHRU Tours, Tours, France. ^22^CRBT, Centre Hospitalo universitaire de Tours, Tours, France. ^23^Pole de Biologie Médicale, Centre Hospitalo universitaire de Tours, Tours, France. ^24^Service des maladies infectieuses, Centre Hospitalo universitaire de Besançon, Besançon, France. ^25^Service des maladies infectieuses, Centre d’investigation clinique, INSERM CIC1431, Centre Hospitalier Universitaire de Besançon, Besançon, France. ^26^Centre de Ressources Biologiques - Filière Microbiologique de Besançon, Centre Hospitalier Universitaire, Besançon, France. ^27^Université de Lorraine, CHRU-Nancy and APEMAC, Infectious and Tropical Diseases, Nancy, France. ^28^Laboratoire de Virologie, CHRU de Nancy Brabois, Vandoeuvre-lès-Nancy, France. ^29^INSERM CIC-EC 1433, Centre Hospitalo universitaire de Nancy, Nancy, France. ^30^Centre de ressources Biologiques, Centre Hospitalo universitaire de Nancy, Nancy, France. ^31^Centre d’Investigation Clinique, INSERM CIC 1408, Centre Hospitalo universitaire de Saint-Étienne, Saint-Étienne, France. ^32^Service des maladies infectieuses, Centre Hospitalo universitaire de Saint-Étienne, Saint-Étienne, France. ^33^Service des maladies infectieuses, CRB42-BTK, Centre Hospitalo Universitaire de Saint-Étienne, Saint-Étienne, France. ^34^Pole Recherche Clinique, INSERM, Paris, France. ^35^IMEA Fondation Léon M’Ba, Paris, France. ^36^INSERM Pôle Recherche Clinique, Paris, France.

## COVID Human Genetic Effort

Laurent Abel^1^, Hassan Abolhassani^2^, Sergio Aguilera-Albesa^3^, Alessandro Aiuti^4^, Ozge Metin Akcan^5^, Nihal Akcay^6^, Gulsum Alkan^7^, Suzan A. Alkhater^8^, Luis Miguel Allende^9^, Yosunkaya Alper^5^, Naima Amenzoui^10^, Mark S. Anderson^11^, Lisa Arkin^12^, Melodie Aubart^13^, Iryna Avramenko^14^, Şehnaz Aydemir^15^, Zeynep Gökçe Gayretli Aydin^16^, Caner Aytekin^17^, Gökhan Aytekin^18^, Selma Erol Aytekin^5^, Silvia Yumi Bando^19^, Kathie Beland^20^, Serkan Belkaya^21^, Catherine M. Biggs^22^, Agurtzane Bilbao Aburto^23^, Geraldine Blanchard-Rohner^24^, Daniel Blázquez-Gamero^9^, Marketa Bloomfield^25^, Dusan Bogunovic^26^, Anastasia Bondarenko^27^, Alessandro Borghesi^28^, Amed Aziz Bousfiha^29^, Oksana Boyarchuk^30^, Petter Brodin^31^, Yenan Bryceson^32^, Giorgia Bucciol^33^, Valeria Calcaterra^34^, Giorgio Casari^4^, Andre Cavalcanti^35^, Jale Bengi Celik^36^, George P. Chrousos^37^, Roger Colobran^38^, Antonio Condino-Neto^39^, Francesca Conti^40^, Megan Cooper^41^, Taner Coskuner^42^, Cyril Cyrus^43^, Enza D’Auria^44^, Selket Delafontaine^45^, Beth A. Drolet^12^, Burcu Bursal Duramaz^46^, Loubna El Zein^47^, Marwa H. Elnagdy^48^, Melike Emiroglu^7^, Emine Hafize Erdeniz^49^, Marianna Fabi^50^, Hagit Baris Feldman^51^, Jacques Fellay^52^, Filip Fencl^25^, Filippos Filippatos^53^, Julie Freiss^54^, Jiri Fremuth^55^, Alenka Gagro^56^, Blanca Garcia-Solis^57^, Gianluca Vergine^58^, Rafaela González-Montelongo^59^, Yahya Gul^60^, Belgin Gülhan^61^, Sara Sebnem Kilic Gultekin^62^, Marta Gut^63^, Rabih Halwani^64^, Lennart Hammarström^65^, Nevin Hatipoğlu^66^, James Heath^67^, Sarah E. Henrickson^68^, Elisa Hernandez-Brito^69^, Ilse Hoffman^70^, Levi Hoste^71^, Elena Hsieh^72^, Antonio Íñigo-Campos^59^, Yuval Itan^73^, Petr Jabandziev^74^, Bahar Kandemir^60^, Saliha Kanık-Yüksek^61^, Hasan Kapakli^75^, Adem Karbuz^76^, Ozgur Kasapcopur^77^, Robin Kechiche^78^, Yasemin Kendir Demirkol^79^, Omer Kilic^80^, Stella Kim Hansen^81^, Adam Klocperk^25^, Yu-Lung Lau^82^, Jan Lebl^25^, José M. Lorenzo-Salazar^59^, Carrie L. Lucas^83^, Majistor Maglorius^84^, Laura Marque^85^, Yeray Novoa Medina^86^, Abián Montesdeoca Melián^87^, Alexios-Fotios A. Mentis^37^, Michele T. Pato^81^, Athanasios Michos^53^, Joshua D. Milner^88^, Trine H. Mogensen^89^, Adrián Muñoz-Barrera^59^, Serdar Nepesov^90^, João Farela Neves^91^, Ashley Ng^12^, Lisa F. P. Ng^92^, Antonio Novelli^93^, Giuseppe Novelli^94^, Fatma Nur Oz^95^, J. Gonzalo Ocejo-Viñals^96^, Satoshi Okada^97^, Zerrin Orbak^98^, Ahmet Osman Kilic^60^, Hind Ouair^29^, Şadiye Kübra Tüter Öz^7^, Tayfun Özçelik^99^, Esra Akyüz Özkan^49^, Aslınur Özkaya Parlakay^100^, Carlos N. Pato^80^, Estela Paz-Artal^9^, Simon Pelham^101^, Isabelle Pellier^54^, Quentin Philippot^84^, Laura Planas-Serra^102^, Samira Plassart^103^, Petra Pokorna^104^, Meltem Polat^95^, Cecilia Poli^105^, Carolina Prando^106^, Laurent Renia^107^, Jacques G. Rivière^108^, Agustí Rodríguez-Palmero^109^, Lucie Roussel^110^, Luis A. Rubio-Rodriguez^59^, Moro Salifu^81^, Lumir Sasek^55^, Laura Sasia^111^, Anna Scherbina^112^, Erica Schmitt^41^, Anna Sediva^25^, Esra Sevketoglu^113^, Katerina Slaba^74^, Ondrej Slaby^114^, Ali Sobh^115^, Jordi Solé-Violán^116^, Pere Soler-Palacin^108^, Lien De Somer^117^, Betül Sözeri^42^, András N. Spaan^118^, Yuriy Stepanovskiy^27^, Stuart G. Tangye^119^, Gonul Tanir^95^, Elizabeth-Barbara Tatsi^53^, Christian W. Thorball^120^, Selda Hancerli Torun^121^, Stuart Turvey^22^, Ahmad Abou Tayoun^122^, Sathishkumar Ramaswamy^123^, Mohammed J. Uddin^124^, Emel Uyar^61^, Juan Valencia-Ramos^125^, Ana Maria Van Den Rym^57^, Hulya Vatansev^60^, Martín Castillo de Vera^126^, François Vermeulen^33^, Donald C. Vinh^110^, Alla Volokha^27^, Horst von Bernuth^127^, Carine Wouters^33^, Aysun Yahşi^61^, Volkan Yarar^75^, Osman Yesilbas^128^, Mehmet Yıldız^77^, Mayana Zatz^129^, Pawel Zawadzki^130^, Gianvincenzo Zuccotti^131^, Shen-Ying Zhang^132^, Jean-Laurent Casanova^133^

^1^Laboratory of Human Genetics of Infectious Diseases, Necker Branch, INSERM U1163, Necker Hospital for Sick Children, Paris, France. ^2^Department of Biosciences and Nutrition, Karolinska Institutet, SE14183, Huddinge, Sweden. ^3^Pediatrics Department, Navarra Health Service Hospital, Pamplona, Spain. ^4^Pediatric Immunohematology, San Raffaele Hospital, Salute San Raffaele University, Italy. ^5^Necmettin Erbakan University, Konya, Turkey. ^6^Bakirkoy Dr. Sadi Konuk Research and Training Hospital, Pediatric Intensive Care Unit, Istanbul, Turkey. ^7^Division of Pediatric Infectious Diseases, Department of Pediatrics, Selcuk University Faculty of Medicine, Konya, Turkey. ^8^College of Medicine, Imam Abdulrahman Bin Faisal University, Dammam, Saudi Arabia; Department of Pediatrics, King Fahad Hospital of the University, Al-Khobar, Saudi Arabia. ^9^Department of Pediatrics, Hospital Universitario 12 de Octubre, Madrid, Spain. ^10^Children Infectious and Clinical Immunology Department, Abderrahim Harouchi Hospital, Faculty of Medicine and Pharmacy, Averroes University Hospital, Hassan 2 University, Casablanca, Morocco. ^11^Diabetes Center, University of California, San Francisco, CA, USA. ^12^University of Wisconsin School of Medicine, Madison, WI, USA. ^13^Laboratory of Human Genetics of Infectious Diseases, Necker Branch, INSERM U1163; Pediatric Neurology Department, Necker-Enfants Malades Hospital, AP-HP, Paris, France. ^14^Department of Propedeutics of Pediatrics and Medical Genetics, Danylo Halytsky Lviv National Medical University, Lviv, Ukraine. ^15^Dr. Ali Kemal Belviranli State Hospital, Konya Turkey. ^16^Department of Pediatrics, Division of Pediatric Infectious Disease, Faculty of Medicine, Karadeniz Technical University, Trabzon, Turkey. ^17^Department of Pediatric Immunology, Dr. Sami Ulus Maternity and Children’s Health and Diseases Training and Research Hospital, Ankara, Turkey. ^18^Konya City Hospital, Konya, Turkey.^19^Laboratory of Pediatric Genomics, Faculty of Medicine, University of Sao Paulo, Sao Paulo, Brazil. ^20^CHU Sainte-Justine, Montreal, QC, Canada. ^21^Department of Molecular Biology and Genetics, Bilkent University, Ankara, Turkey. ^22^Department of Pediatrics, University of British Columbia, Vancouver, BC, Canada; BC Children’s Hospital Research Institute, Vancouver, BC, Canada. ^23^Servicio de Pediatría, Hospital Universitario Cruces, Spain. ^24^Unit of Immunology and Vaccinology, Division of General Pediatrics, Department of Pediatrics, Gynecology and Obstetrics, Geneva University Hospitals, University of Geneva, Geneva, Switzerland. ^25^Department of Pediatrics, 2nd Faculty of Medicine, Charles University in Prague and Motol University Hospital, Prague, Czech Republic. ^26^Center for Inborn Errors of Immunity, Precision Immunology Institute, Mindich Child Health and Development Institute, Department of Microbiology, Department of Pediatrics, Icahn School of Medicine at Mount Sinai, New York, NY, USA. ^27^Pediatric Infectious Disease and Pediatric Immunology Department, Shupyk National Healthcare University of Ukraine, Kyiv, Ukraine. ^28^Neonatal Intensive Care Unit, Fondazione IRCCS Policlinico San Matteo, Pavia, Italy; Fellay Lab, Ecole Polytechnique Fédérale de Lausanne (EPFL), Lausanne, Switzerland. ^29^Clinical Immunology Unit, Casablanca Children’s Hospital, Ibn Rochd Medical School, King Hassan II University, Casablanca, Morocco. ^30^Department of Children’s Diseases and Pediatric Surgery, I. Horbachevsky Ternopil National Medical University, Ukraine. ^31^Science for Life Laboratory, Department of Women’s and Children’s Health, Karolinska Institutet, Stockholm, Sweden. ^32^Center for Hematology and Regenerative Medicine, Department of Medicine, Karolinska Institute, Stockholm, Sweden. ^33^Department of Pediatrics, University Hospitals Leuven, Belgium. ^34^Department of Pediatrics, Buzzi Children’s Hospital, Milan, Italy; Department of Internal Medicine, University of Pavia, Pavia, Italy. ^35^Department of Pediatrics, Clinical Hospital of Federal University of Pemambuco, Recife, Brazil. ^36^Department of Anesthesiology and Reanimation, Selcuk University Faculty of Medicine, Konya, Turkey. ^37^University Research Institute of Maternal and Child Health and Precision Medicine, National and Kapodistrian University of Athens, Athens, Greece. ^38^Immunology Division, Genetics Department, Vall d’Hebron Research Institute, Vall d’Hebron Barcelona Hospital Campus, Universitat Autònoma de Barcelona, Barcelona, Spain. ^39^Department of Immunology, Institute of Biomedical Sciences, University of Sao Paulo, Sao Paulo, Brazil. ^40^Pediatric Unit-IRCCS Azienda OspedalieroUniversitaria di Bologna, Bologna, Italy. ^41^Division of Rheumatology and Immunology, Department of Pediatrics, Washington University in St. Louis, St. Louis, MO, USA. ^42^Division of Pediatric Rheumatology, Umraniye Training and Research Hospital, University of Health Sciences, Istanbul, Turkey. ^43^Department of Biochemistry, College of Medicine, Imam Abdulrahman Bin Faisal University, Saudi Arabia. ^44^Department of Pediatrics, Buzzi Children’s Hospital, Milan, Italy. ^45^Department of Pediatrics, University Hospitals Leuven, Laboratory for Inborn Errors of Immunity, KU Leuven, Leuven, Belgium. ^46^University of Health Sciences, Kanuni Sultan Suleyman Training and Research Hospital, Istanbul, Turkey. ^47^Lebanese University, Faculty of Sciences I, Biology Department, Rafic Hariri Campus, Beirut, Lebanon. ^48^Medical Biochemistry and Molecular Biology Department, Faculty of Medicine, Mansoura University, Mansoura, Egypt. ^49^Ondokuz Mayis University, Samsun, Turkey. ^50^Pediatric Emergency Unit, Scientific Institute for Research and Healthcare (IRCCS), Sant’Orsola Hospital, Bologna, Italy. ^51^The Genetics Institute, Tel Aviv Sourasky Medical Center and Sackler Faculty of Medicine, Tel Aviv University, Tel Aviv, Israel. ^52^School of Life Sciences, École Polytechnique Fédérale de Lausanne, Lausanne, Switzerland; Precision Medicine Unit, Biomedical Data Sciences Center, Lausanne University Hospital and University of Lausanne, Lausanne, Switzerland; Swiss Institute of Bioinformatics, Lausanne, Switzerland. ^53^First Department of Pediatrics, National and Kapodistrian University of Athens, Athens, Greece; University Research Institute of Maternal and Child Health and Precision Medicine, National and Kapodistrian University of Athens, Athens, Greece. Unité d’Hématologie-Immunologie-Oncologie Pédiatrique, Centre Hospitalier Universitaire d’Angers, Angers, France. ^55^Department of Pediatrics - PICU, Faculty of Medicine in Pilsen, Charles University in Prague, Czech Republic. ^56^Department of Pediatrics, University of Zagreb School of Medicine, Children’s Hospital Zagreb, Zagreb, Josip Juraj Strossmayer University of Osijek, Medical Faculty Osijek, Osijek, Croatia. ^57^Laboratory of Immunogenetics of Human Diseases, IdiPAZ Institute for Health Research, La Paz Hospital, Madrid, Spain. ^58^Unità Operativa Complessa Pediatria, Ospedale degli Infermi di Rimini, Rimini, Italy. ^59^Genomics Division, Instituto Tecnológico y de Energías Renovables (ITER), Santa Cruz de Tenerife, Spain. ^60^Necmettin Erbakan University, Konya, Turkey. ^61^Ankara City Hospital, Ankara, Turkey. ^62^Pediatric Immunology-Rheumatology Division, Uludag University Medical Faculty, Department of Pediatrics, Bursa, Turkey. ^63^CNAG-CRG, Centre for Genomic Regulation (CRG), The Barcelona Institute of Science and Technology (BIST), Barcelona, Spain. ^64^College of Medicine, University of Shajah, Shajah, UAE. ^65^Division of Clinical Immunology, Department of Laboratory Medicine, Karolinska University Hospital Huddinge, Stockholm, Sweden. ^66^Pediatric Infectious Diseases Unit, Bakirkoy Dr. Sadi Konuk Training and Research Hospital, University of Health Sciences, Istanbul, Turkey. ^67^Institute of Systems Biology, Seattle, WA, USA. ^68^Children’s Hospital of Philadelphia, Division of Allergy Immunology, Philadelphia, PA, USA; Department of Microbiology, Perelman School of Medicine, University of Pennsylvania, Philadelphia, PA, USA. ^69^Department of Immunology, Hospital Universitario de Gran Canaria Dr. Negrín, Canarian Health System, Las Palmas de Gran Canaria, Spain. ^70^Department of Pediatrics, University Hospitals Leuven, Belgium. ^71^Primary Immunodeficiency Research Lab, Center for Primary Immunodeficiency Ghent, Ghent University Hospital, Ghent, Belgium. ^72^Department of Pediatrics, Section of Allergy and Immunology, Department of Immunology and Microbiology, University of Colorado Anschutz Medical Campus, Children’s Hospital Colorado, Aurora, CO, USA. ^73^Icahn School of Medicine at Mount Sinai, New York, NY, USA. ^74^Department of Pediatrics, University Hospital Brno and Faculty of Medicine, Masaryk University, Brno, Czech Republic. ^75^Balikesir City Hospital, Balikesir, Turkey. ^76^Prof. Dr. Cemil Taşcıoglu City Hospital, Istanbul, Turkey. ^77^Department of Pediatric Rheumatology, Cerrahpasa Medical School, Istanbul University-Cerrahpasa, Istanbul, Turkey. ^78^Service de Rhumatologie Pediatrique, CHU Bicetre, France. Department of Pediatric Genetics, Health Sciences University, Umraniye Education and Research Hospital, Istanbul, Turkey. ^80^Eskişehir Osmangazi University, Faculty of Medicine, Clinic of Pediatric Infectious Diseases, Eskişehir, Turkey. ^81^Institute for Genomic Health, SUNY Downstate, Health Science University, Brooklyn, NY, USA. ^82^Department of Paediatrics and Adolescent Medicine, LKS Faculty of Medicine, the University of Hong Kong, Hong Kong. ^83^Department of Immunobiology, Yale University School of Medicine, New Haven, CT, USA. ^84^Laboratory of Human Genetics of Infectious Diseases, Necker Branch, INSERM U1163, Necker Hospital for Sick Children; Paris Descartes University, Imagine Institute, Paris, France. ^85^Immunodeficiencies and Infectious Diseases Unit, Pediatric Department, Centro Materno-Infantil do Norte, Centro Hospitalar Universitario do Porto, Porto, Portugal. ^86^Department of Pediatrics, Complejo Hospitalario Universitario Insular-Materno Infantil, Canarian Health System, Las Palmas de Gran Canaria, Spain. ^87^Guanarteme Health Care Center, Canarian Health System, Las Palmas de Gran Canaria, Spain. ^88^Department of Pediatrics, Columbia University Irving Medical Center, New York, NY, USA. ^89^Department of Biomedicine, Aarhus University, Aarhus, Denmark. ^90^Pediatric Allergy and Immunology Unit, Istanbul Medipol Üniversity, Istanbul, Turkey. ^91^Primary Immunodeficiencies Unit, Hospital Dona Estefânia, CHULC, EPE; CEDOC, Center for Chronic Diseases, Lisbon, Portugal. ^92^A*STAR Infectious Disease Labs, Agency for Science, Technology and Research, Singapore. ^93^Translational Cytogenomics Research Unit, Bambino Gesù Children’s Hospital, IRCCS, Rome, Italy. ^94^Department of Biomedicine and Prevention, Tor Vergata University of Rome, Italy. ^95^Department of Pediatric Infectious Disease, SBU Ankara Dr. Sami Ulus Maternity Child Health and Diseases Training and Research Hospital, Ankara, Turkey. ^96^Department of Immunology, Hospital Marques de Valdecilla, Santander, Spain. ^97^Department of Pediatrics, Hiroshima University Graduate School of Biomedical and Health Sciences, Hiroshima, Japan. ^98^Department of Pediatrics, Ataturk University, Erzurum, Turkey. ^99^Department of Molecular Biology and Genetics, Bilkent University, Ankara, Turkey. ^100^Yildirim Beyazit University, Ankara City Hospital, Ankara, Turkey. ^101^St. Giles Laboratory of Human Genetics of Infectious Diseases, Rockefeller Branch, The Rockefeller University, New York, NY, USA. ^102^Neurometabolic Diseases Laboratory, IDIBELL–Hospital Duran I Reynals; CIBERER U759, ISIiii Madrid, Spain. ^103^Service de Rhumatologie pédiatrique, Hôpital Femme-Mère-Enfant, Groupement Hospitalier Est - Bâtiment “Pinel”, Bron, France. ^104^Central European Institute of Technology, Masaryk University, Brno, Czech Republic. ^105^Immunogenetics and Translational Immunology Program, Instituto de Ciencias e Innovación en Medicina (ICIM), School of Medicine, Clínica Alemana-Universidad del Desarrollo, Santiago de Chile, Chile. ^106^Faculdades Pequeno Príncipe, Instituto de Pesquisa Pelé Pequeno Príncipe, Curitiba, Brazil. ^107^A*STAR Infectious Disease Labs, Agency for Science, Technology and Research, Singapore; Lee Kong Chian School of Medicine, Nanyang Technological University, Singapore; School of Biological Sciences, Nanyang. Technological University, Singapore. ^108^Pediatric Infectious Diseases and Immunodeficiencies Unit, Vall d’Hebron Research Institute, Vall d’Hebron Barcelona Hospital Campus, Universitat Autònoma de Barcelona, Barcelona, Spain. ^109^Palmero Pediatrics Department, University Hospital Germans Trias i Pujol, Badalona, Barcelona, Spain. ^110^Department of Medicine, Division of Infectious Diseases, McGill University Health Centre, Montréal, QC, Canada; Infectious Disease Susceptibility Program, Research Institute, McGill University Health Centre, Montréal, QC, Canada. ^111^Hospital Infantil Municipal de Córdoba, Córdoba, Argentina. ^112^Dmitry Rogachev National Medical Research Center of Pediatric Hematology, Moscow, Russia. ^113^Pediatric Intensive Care Unit, Bakirkoy Dr. Sadi Konuk Training and Research Hospital, University of Health Sciences, Istanbul, Turkey. ^114^Department of Biology UKB Kamenice, Masaryk University / Faculty of Medicine, Brno, Czech Republic. ^115^Department of Pediatrics, Mansoura University Children’s Hospital, Faculty of Medicine, Mansoura University, Mansoura, Egypt. ^116^Critical Care Unit, Hospital Universitario de Gran Canaria Dr. Negrín, Canarian Health System, Las Palmas de Gran Canaria, Spain; Universidad Fernando Pessoa, Canarias, Spain; CIBER de Enfermedades Respiratorias. Instituto de Salud Carlos III, Madrid, Spain. ^117^Department of Pediatrics, University Hospitals Leuven, Leuven, Belgium. ^118^St. Giles Laboratory of Human Genetics of Infectious Diseases, Rockefeller Branch, The Rockefeller University, New York, NY, USA; Department of Medical Microbiology, University Medical Center Utrecht, Utrecht, Netherlands. ^119^Garvan Institute of Medical Research, Darlinghurst, NSW, Australia; St Vincent’s Clinical School, Faculty of Medicine, UNSW Sydney, Sydney, NSW, Australia. ^120^Precision Medicine Unit, Biomedical Data Sciences Center, Lausanne University Hospital and University of Lausanne, Lausanne, Switzerland. ^121^Department of Pediatric Infectious Disease, Faculty of Medicine, Istanbul University, Istanbul, Turkey. ^122^Genomics Center, Al Jalila Children’s Specialty Hospital, Dubai, UAE; Center for Genomic Discovery, Mohammed Bin Rashid University, Dubai, UAE. ^123^Genomics Center, Al Jalila Children’s Specialty Hospital, Dubai, UAE. ^124^College of Medicine, Mohammed Bin Rashid University of Medicine and Health Sciences, Dubai, UAE; Cellular Intelligence (Ci) Lab, GenomeArc Inc., Toronto, ON, Canada. ^125^Pediatrics Department, Division Pediatric Intensive Care Unit, Hospital Universitario de Burgos, Burgos, Spain. ^126^Doctoral Health Care Center, Canarian Health System, Las Palmas de Gran Canaria, Spain. ^127^Department of Pediatric Pneumology, Immunology and Intensive Care, Charité Universitätsmedizin, Berlin University Hospital Center, Berlin, Germany; Labor Berlin GmbH, Department of Immunology, Berlin, Germany; Berlin Institutes of Health (BIH), Berlin-Brandenburg Center for Regenerative Therapies, Berlin, Germany. ^128^Department of Pediatrics, Division of Pediatric Critical Care Medicine, Faculty of Medicine, Karadeniz Technical University, Trabzon, Turkey. ^129^Biosciences Institute, University of São Paulo, São Paulo, Brazil. ^130^MNM Diagnostics, Poznań, Poland. ^131^Department of Pediatrics, Buzzi Children’s Hospital, Milan, Italy; Department of Biomedical and Clinical Sciences, University of Milan, Milan, Italy. ^132^Laboratory of Human Genetics of Infectious Diseases, Necker Branch, INSERM U1163, Necker Hospital for Sick Children, Paris, France; Laboratory of Human Genetics of Infectious Diseases, Rockefeller Branch, The Rockefeller University, New York, NY, USA. ^133^Necker Hospital for Sick Children and INSERM, Paris, France; The Rockefeller University and Howard Hughes Medical Institute, New York, NY, USA.
