# Supplementary material for: Inborn errors of OAS–RNase L in SARS-CoV-2–related multisystem inflammatory syndrome in children
Source: Science. 2023 Feb 10;379(6632):eabo3627. doi: 10.1126/science.abo3627 (PMC10451000; doi:10.1126/science.abo3627)
Supplement: Supplementary file 2 — Materials and Methods Figs. S1 to S9 Tables S1 to S3 References (92–100) [file science.abo3627_sm.pdf]

Science AAAS

Supplementary Materials for

Inborn errors of OAS–RNase L in SARS-CoV-2–related multisystem inflammatory syndrome in children

Danyel Lee *et al*.

Corresponding author: Shen-Ying Zhang, shzh289@rockefeller.edu

Science **379**, eabo3627 (2023)
DOI: 10.1126/science.abo3627

The PDF file includes:

Materials and Methods
Figs. S1 to S9
Tables S1 to S3
References

Other Supplementary Material for this manuscript includes the following:

MDAR Reproducibility Checklist
Data S1 and S2

Materials and Methods

Whole-exome and whole-genome sequencing

Genomic DNA was extracted from whole blood. Whole-exome sequencing (WES) or whole-genome sequencing (WGS) was performed at several sequencing centers, including the Genomics Core Facility of the Imagine Institute (Paris, France), the Yale Center for Genome Analysis (USA), the New-York Genome Center (NY, USA), the American Genome Center (TAGC, USUHS, Bethesda, USA), and the Genomics Division-ITER of the Canarian Health System sequencing hub (Canary Islands, Spain). For WES, libraries were prepared with the TruSeq DNA Sample Prep Kit (Illumina), the Twist Bioscience kit (Twist Human Core Exome Kit), the xGen Exome Research Panel from Integrated DNA Technologies (IDT xGen), the Agilent SureSelect V6 kit, the Agilent SureSelect V7 kit, the Agilent SureSelect Human All Exon 50 Mb kit, the SeqCap EZ MedExome kit from Roche, the SeqCap EZ Human Exome Kit v3.0 from Roche, or the Nextera Flex for Enrichment-Exome kit (Illumina). Massively parallel sequencing was performed on a HiSeq 2000-4000 or NovaSeq6000 system (Illumina). The sequences were analyzed with the Genome Analysis Software Kit (GATK) (version 3.4-46 or 4) best-practice pipeline for WES data (*92*). We aligned the reads obtained with the human reference genome (hg19), using the maximum exact matches algorithm in the Burrows–Wheeler aligner (BWA) (*93*). PCR duplicates were removed with Picard tools (picard.sourceforge.net). The GATK base quality score recalibrator was applied to correct sequencing artifacts. Genotyping was performed with GATK GenotypeGVCFs in the interval intersecting all the capture kits ±50 bp. Sample genotypes with a coverage <8X, a genotype quality (GQ)<20, or a proportion of reads for the least covered allele (reference or variant allele) over the total number of reads covering the position (minor read ratio, MRR) <20% were filtered out. We filtered out variant sites that (i) fell in low-complexity or decoy regions, (ii) were multiallelic, with more than four alleles, (iii) had more than 10% missing genotypes in our cohort, and (iv) spanned more than 15 nucleotides. Variant effects were predicted with the Ensembl Variant Effect Predictor (VEP)(*94*) and Loss-Of-Function Transcript Effect Estimator (LOFTEE) Plugin, with the Ensembl GRCh37.75 reference database, retaining the most deleterious annotation obtained from the Ensembl protein-coding transcripts overlapping the RefSeq transcripts. Predicted loss-of-function variants flagged as being of low confidence by LOFTEE were excluded, together with variants curated as "not LoF" in gnomAD v2.1.

In vitro peripheral blood mononuclear cell stimulation with SARS-CoV-2

We performed single-cell RNAseq (scRNAseq) on SARS-CoV-2- and mock-stimulated PBMCs sampled from four individuals with inborn errors of the OAS–RNaseL pathway (P1 with OAS1 deficiency, P2 and P3 with OAS2 deficiency, P5 with RNase L deficiency), three individuals with inborn errors of type I IFN immunity (one TLR7-deficient patient (*7*), one IRF9-deficient patient (*95*), one IFNAR1-deficient patient (*57*)), and eight healthy donors: one pediatric control and one adult control with a history of past asymptomatic SARS-CoV-2 infection, two pediatric controls and four adult controls with no history of prior SARS-CoV-2 infection. Cryopreserved PBMCs were thawed in a 37°C water bath, centrifuged at 300*g* for 10 min at room temperature, and resuspended at a density of 2×10^6 cells/ml in 25 cm^2 flasks. Cells were left to rest overnight (i.e., approximately 14 hours) at 37°C in RPMI medium supplemented with 10% heat-inactivated fetal bovine serum (R10). The following morning, the PBMCs were washed and resuspended at a density of 3.3×10^6 cells/ml in R10. Then, 120 µl of suspension from each sample, containing 4×10^5 cells, was plated in a 96-well untreated plate for each of the three conditions. We added 80 µl of R10 (mock) or SARS-CoV-2 (4×10^5 focus-forming units diluted in R10) to the cells to achieve a MOI of 1 and an optimal density of PBMCs at 2×10^6 cells/ml. The cells were incubated at 37°C for 6 hours, in a biosafety level 3 (BSL-3) facility at Institut Pasteur, Paris. The plates were centrifuged at 300*g* for 10 min. Samples were then resuspended in phosphate-buffered saline (PBS) supplemented with 0.04% bovine serum albumin (BSA) and multiplexed in eight pools according to a pre-established design, to control for effects related to processing and sequencing batches. The cells in each pool were counted with a Cell Countess II automated cell counter, and cell density was adjusted to 1000 viable cells/µl in PBS supplemented with 0.04% BSA.

ScRNAseq library preparation and sequencing of PBMCs stimulated in vitro with SARS-CoV-2

ScRNA-sequencing cDNA libraries were generated with a Chromium Controller (10X Genomics, Pleasanton, CA) according to the manufacturer’s instructions for the Chromium Single Cell 3′ Library and Gel Bead Kits (v3). Library quality and concentration were assessed with an Agilent 2100 Bioanalyzer and a Qubit fluorometer. The final products were sent to Macrogen Inc. for high-throughput sequencing on a HiseqX platform (Illumina Inc.).

Data processing for scRNAseq of PBMCs stimulated with SARS-CoV-2

Paired-end sequencing reads from each of the eight scRNA-seq cDNA libraries were independently mapped onto the concatenated human (GRCh38) and SARS-CoV-2 genome sequences with *CellRanger* (v4.0.0). Cell-containing droplets were assigned to their sample of origin with Demuxlet (v0.1), making use of the genotyping data available for every individual. We loaded feature-barcode matrices for all cell-containing droplets identified as singlets by Demuxlet in each scRNA-seq library onto a SingleCellExperiment (v1.14.1) object. Barcodes associated with low-quality or dying cells were removed from the data set by a hard threshold-based filtering strategy with three metrics: within each sequencing library, cells with fewer than 1500 total counts, 500 detected features, or with a mitochondrial gene content exceeding 20% were removed from the analysis.

We then log-normalized raw UMI counts with a unit pseudocount and library size factors (i.e., the number of reads associated with each barcode) were calculated with *quickClusters* and *computeSumFactors* from the *scran* package (v1.20.1). We then calculated the mean and variance of log-counts for each gene and broke the variance down into a biological and a technical component with the *fitTrendPoisson* and *modelGeneVarByPoisson* functions of *scran*. Briefly, this approach assumes that technical noise is Poisson-distributed and simulates Poisson-distributed data to derive the mean-variance relationship expected in the absence of biological variation. The excess variance relative to the null hypothesis is then considered to be the biological variance. We retained only those genes for which the biological variance component was >0 with a false discovery rate (FDR) below 1%. We used this filtered feature set and the technical variance component modeled from the data to perform principal component analysis (PCA), discarding later principal components (PCs), which probably capture technical noise, with *denoisePCA* in *scran*.

We found that doublets (i.e., barcodes assigned to cells from different individuals captured in the same droplet) were likely to be nearest neighbors (NN) when projected onto a reduced-dimension space. We therefore used a *k*-NN approach to weed out cryptic doublets (i.e., barcodes assigned for different cells from the same individual captured in the same doublet). Barcodes assigned as

singlets by Demuxlet but with more than 5/25 doublet NNs in the transcriptomic PC space were reassigned as doublets and excluded from further analyses.

Following this first round of preprocessing, we performed a second round of UMI count normalization, feature selection, and dimensionality reduction on the cleaned data, to prevent bias due to the presence of low-quality cells and cryptic doublets. We homogenized sequencing depth between batches (i.e., sequencing libraries) by using *multiBatchNorm* from *batchelor* (v1.8.1) to scale library size factors according to the ratio of mean counts between batches. Differences in mean-variance trends between batches were taken into account by applying *modelGeneVarByPoisson* separately for each sequencing library, and then combining the results for all batches with *combineVar*from *scran*. We then bound all eight separate preprocessed feature-barcode matrices into a single merged SingleCellExperiment object, log-normalized UMI counts with the scaled size factors, and selected genes with a mean log-expression over 0.01 or a biological variance compartment over 0.001. Based on this set of highly variable genes and the variance breakdown, we performed PCA on the whole data set with *denoisePCA* and corrected PCs using Harmony (v0.1.0) to adjust for library effects.

<u>Data analysis for scRNAseq of PBMCs stimulated with SARS-CoV-2</u>

We performed cluster-based cell-type identification in each set of conditions according to a four-step procedure. We first performed low-resolution (res. parameter=0.8) shared nearest neighbors graph-based (*k*=25) clustering with *FindClusters* from *Seurat* (v4.1.1) with assignment to one of three meta-clusters (i.e., myeloid, B lymphoid, and T/NK lymphoid) based on the transcriptional profiles of canonical markers (e.g., *CD3E-F*, *CD14*, *FCGR3A*, *MS4A1*). We then performed a second round of clustering at higher resolution (res. parameter=3) within each meta-cluster and set of conditions. We systematically tested for differential expression between each cluster and the other clusters in the same meta-cluster and conditions to define unbiased markers ($|\log_2 FC| > 0$, $FDR < 0.01$) for each cluster. We then used these genes (both upregulated and downregulated) to assign each cluster manually to one of six different cell types, which we collapsed into five major immune lineages. Thus, by calling cell types on the basis of high-resolution, homogeneous clusters assigned independently for each lineage and set of conditions, we were able to preserve much of the diversity in our data set, while avoiding potentially confusing effects of stimulation conditions.

However, we did observe clusters with marker profiles suggestive of mixed identity. Some of these profiles were consistent with mixtures of biologically similar cell types (e.g., *CD3E*, *CD8A*, *NKG7*, and *CD16* for a mixture of cytotoxic CD8$^+$ T and NK cells), whereas others (fewer in number) were considered inconsistent (e.g., *CD3E*, *CD19*) and were discarded. For the resolution of consistent mixtures, the fourth and final step of our procedure was linear discriminant analysis. For any cluster of mixed identity AB, we built a training data set from 2000 observations sampled from the set of cells called as A or B, preserving their respective frequencies in the whole data set. We then used a model trained on these data to predict identities in the mixed cluster.

For the pseudobulk analysis, pseudobulk estimation, normalization, and batch correction were performed as follows: individual variation in gene expression was quantified at two different resolutions (five immune lineages and six cell types). We aggregated raw UMI counts from all high-quality single-cell transcriptomes (n=46,157) into bulk expression estimates by summing gene expression values across all cells assigned to the same immune lineage/cell type and sample (i.e., individual and conditions) with the *aggregateAcrossCells* function from *scuttle*. We then normalized the raw aggregated UMI counts by library size, generating 160 lineage-wise (16 donors×2 conditions×5 lineages) and 192 cell type-wise (32 samples×6 cell types) pseudobulk counts-per-million (CPM) values for all 14,557 genes in our data set. CPM values were then $\log_2$-transformed, with an offset of 1 to avoid values of infinity and to stabilize variation for genes with low levels of expression. Genes with a mean CPM below 1 across all conditions and immune lineages/cell types were considered to be not expressed and were discarded from further analyses. Unwanted experimental variation was minimized by using the *lmer* function from the *lme4* package to fit a linear model of the form $log_2(CPM_i+1)=\alpha+IID_i+LIB_i+\varepsilon_i$ to each set of conditions and immune lineage/cell type, where CPM_i is the expression in sample i (i.e., one replicate of a given individual and set of experimental conditions), α is the intercept, $IID_i\sim N(0,\sigma^2_{IND})$ captures the effect of individual i on gene expression, and $LIB_i\sim N(0,\sigma^2_{LIB})$ captures the effect of 10X Genomics library preparation. We then subtracted the estimated value for library effects (provided by the *ranef* function) from the transformed CPMs for each sample, to obtain batch-corrected CPM values. Finally, we averaged the batch-corrected CPM values obtained across different replicates for the same individual and set of conditions to obtain final estimates of gene expression. We define as differentially expressed genes those genes with an absolute log2-fold change (FC) in expression upon SARS-CoV-2 stimulation greater than 0.2 at a false discovery rate (FDR) of 1%

(|log2FC(COV|> 0.2, FDR < 0.01). We assessed the effect of OAS–RNase L deficiency on the transcriptional response to SARS-CoV-2 stimulation by calculating a t statistic for each gene in each cell type with the FC estimated by our linear model in the OAS–RNase L-deficient (OAS–RNase L MT) and control (Ctrls) contexts, respectively, and the standard errors of the corresponding estimators. We considered genes to be significantly affected by OAS–RNase L deficiency if $|t|\geq1.96$.

We modeled the effects of immunodeficiency on gene expression and the response to stimuli as follows: we estimated the effects of OAS–RNaseL/IFN pathway deficiencies on gene expression in each individual (n=16) and set of conditions by constructing, for each gene, lineage (i.e., myeloid, B, $CD4^+$ T, $CD8^+$ T or NK cells) and set of conditions (i.e., SARS-CoV-2–stimulated or mock-stimulated) a separate linear model of the form $Y_{i,j,k,l}=\alpha+\beta^{OAS}\cdot I^{OAS}{}_i+\beta^{IFN}\cdot I^{IFN}+Z^T{}_i\cdot\gamma+\varepsilon_i$. In this model, $Y_{i,j,k,l}$ is the expression (log-normalized counts) of gene j in individual i's cells from lineage k in condition l. $I^{OAS}{}_i$ and $I^{IFN}{}_i$ are dummy variables equal to 1 for OAS–RNaseL- and IFN-deficient individuals, respectively, and equal to 0 otherwise. The set of core covariates of individual i, including age, sex, COVID-19 status, and cellular mortality (i.e., proportion of dying cells in each thawed vial, as a proxy for sample quality) are encoded in Z_i. In addition, ε_i corresponds to the normally distributed residuals, and α, β^{OAS}, β^{IFN}, γ are the fitted parameters of the models. In particular, α is the intercept, β^{OAS} (β^{IFN}) is the $\log_2$ fold-change difference in expression between groups (OAS–RNase L-deficient, IFN-deficient, and control individuals), and γ captures the effects of the set of core covariates on gene expression.

We reasoned that differences in the variance of gene expression between immune deficiency statuses would inflate the number of false positives. We therefore used the *vcovHC* function from *sandwich* (v2.5-1) with the type='HC3' option to calculate sandwich estimators of variance robust to residual heteroskedasticity and estimated the coefficients and their standard error with the *coeftest* function of *lmtest* (v0.9-40). We estimated the effect of OAS–RNase L or IFN pathway deficiencies on the response to stimulation of each gene in each sample by fitting the linear model described above, but with the gene's $\log_2$-fold change in expression upon stimulation as the response variable. Due to the small sample size, genes with a nominal $p<1\%$ and $|\beta|>0.2$ were considered to be differentially expressed/responding.

scRNAseq on PBMCs obtained from P5 during the acute and convalescent phases of MIS-C

Cryopreserved PBMCs from P5 (aged 4 years) sampled during the acute (9 days after MIS-C onset) and convalescent (~1 month after onset) phases were analyzed, together with cells from one healthy adult and two pediatric controls. The cells were subjected to scRNAseq. Briefly, thawed cells were washed with medium and filtered through a MACS SmartStrainer with 70-µm pores (Miltenyi Biotec, Cat: 130-098-462) to remove large debris. Cells were then washed three times with PBS + 0.5% FBS and finally filtered again with a Falcon 40-µm-mesh Cell Strainer (Corning, Cat: 352340) before capture with the 10X Genomics Chromium chip. Libraries were prepared with the Chromium Single-Cell 3′ Reagent Kit (v3 Chemistry) and sequenced with an Illumina NovaSeq 6000 sequencer (S1 flowcell). Sequences were preprocessed with CellRanger v6.1.2 on the 10X Genomics Cloud Analysis platform (https://www.10xgenomics.com/products/cloud-analysis). Approximately 11,000–16,000 cells were captured per sample, with a mean of at least ~22,000 reads per cell.

The data generated during this study were analyzed in an integrative manner with historical controls from the laboratory (one pediatric and seven adult controls), publicly available control PBMC datasets downloaded from the 10X Genomics web portal (https://support.10xgenomics.com/single-cell-gene-expression/datasets), and a previously published dataset for patients with acute SARS-CoV-2 infection and MIS-C (GEO accession: GSE167029). In addition, two other previously published sets of scRNAseq data for pediatric healthy controls and children with acute SARS-CoV-2 infection or MIS-C (GSE166489; Zenodo DOI: https://doi.org/10.5281/zenodo.5524378) were used for an independent cohort analysis. Data were manually curated according to the percentage of mitochondrial genes and the numbers of genes and transcripts detected. Curated datasets were integrated with Harmony (*96*). Two sequential graph-based clustering analyses were performed. The second round of clustering focused on memory and effector T cells and NK cells to achieve the highest resolution of cellular subsets. Clusters were identified manually with the SingleR pipeline (*97*) guided by MonacoImmuneData (*98*). The TotalSeq datasets derived from 10X also provided information about the identity of each cluster. Pseudobulk differential expression analysis was conducted with DESeq2 (*99*), with the exclusion of all public datasets. GSEA was conducted with the fgsea package by projecting the ranking of the fold-change in expression onto the Hallmark genesets

(*71*). Intercellular communication analysis was conducted with CellChat (*74*). All analyses were performed in R v4 (*100*).

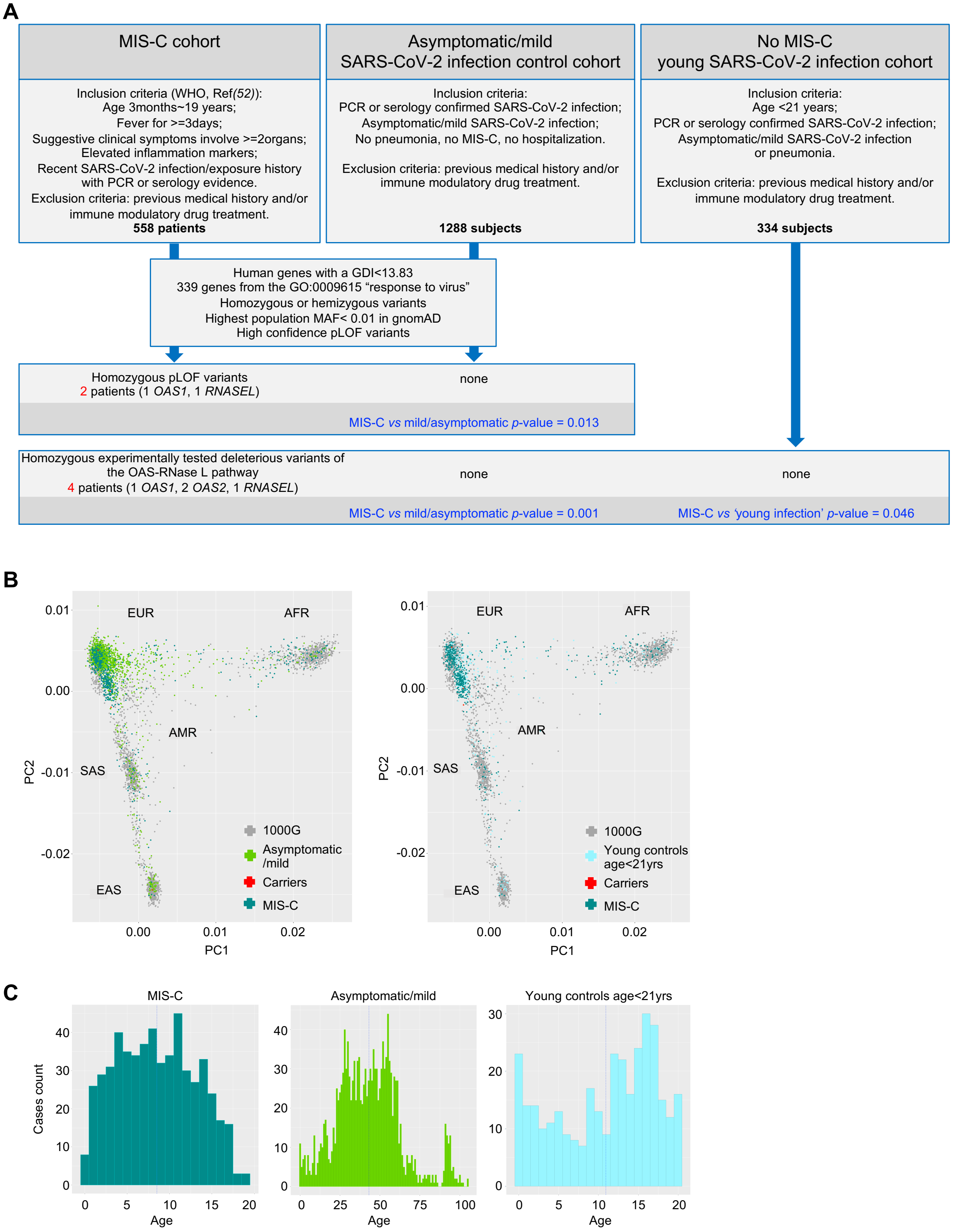
A
MIS-C cohort
Inclusion criteria (WHO, Ref(52)):
Age 3months~19 years;
Fever for >=3days;
Suggestive clinical symptoms involve >=2organs;
Elevated inflammation markers;
Recent SARS-CoV-2 infection/exposure history
with PCR or serology evidence.
Exclusion criteria: previous medical history and/or
immune modulatory drug treatment.
558 patients
Asymptomatic/mild
SARS-CoV-2 infection control cohort
Inclusion criteria:
PCR or serology confirmed SARS-CoV-2 infection;
Asymptomatic/mild SARS-CoV-2 infection;
No pneumonia, no MIS-C, no hospitalization.
Exclusion criteria: previous medical history and/or
immune modulatory drug treatment.
1288 subjects
No MIS-C
young SARS-CoV-2 infection cohort
Inclusion criteria:
Age <21 years;
PCR or serology confirmed SARS-CoV-2 infection;
Asymptomatic/mild SARS-CoV-2 infection
or pneumonia.
Exclusion criteria: previous medical history and/or
immune modulatory drug treatment.
334 subjects
Human genes with a GDI<13.83
339 genes from the GO:0009615 "response to virus"
Homozygous or hemizygous variants
Highest population MAF< 0.01 in gnomAD
High confidence pLOF variants
Homozygous pLOF variants
2 patients (1 OAS1, 1 RNASEL)
none
MIS-C vs mild/asymptomatic p-value = 0.013
Homozygous experimentally tested deleterious variants of
the OAS-RNase L pathway
4 patients (1 OAS1, 2 OAS2, 1 RNASEL)
none
none
MIS-C vs mild/asymptomatic p-value = 0.001
MIS-C vs 'young infection' p-value = 0.046
B
EUR
AFR
AMR
SAS
EAS
PC2
PC1
0.01
0.00
-0.01
-0.02
0.00
0.01
0.02
1000G
Asymptomatic /mild
Carriers
MIS-C
Young controls age<21yrs
C
MIS-C
Asymptomatic/mild
Young controls age<21yrs
Cases count
Age

Fig. S1. Enrichment in homozygous variants of the OAS–RNase L pathway in MIS-C patients

(**A**) Schematic overview of the genetic approaches leading to the discovery of an enrichment in homozygous deleterious variants of the OAS–RNase L pathway in MIS-C patients. (**B**) Principal component analysis (PCA) of ethnic heterogeneity for the MIS-C cohort, 1288 SARS-CoV-2–infected controls, and a control cohort of 334 young patients with asymptomatic/mild infection (175 patients) or COVID-19 pneumonia (159 patients). Data from the 1000 Genomes project are shown as ethnicity controls. (**C**) Age distribution of the MIS-C cohort, 1288 SARS-CoV-2–infected controls, and a control cohort of 334 young patients with asymptomatic/mild infection (175 patients) or COVID-19 pneumonia (159 patients).

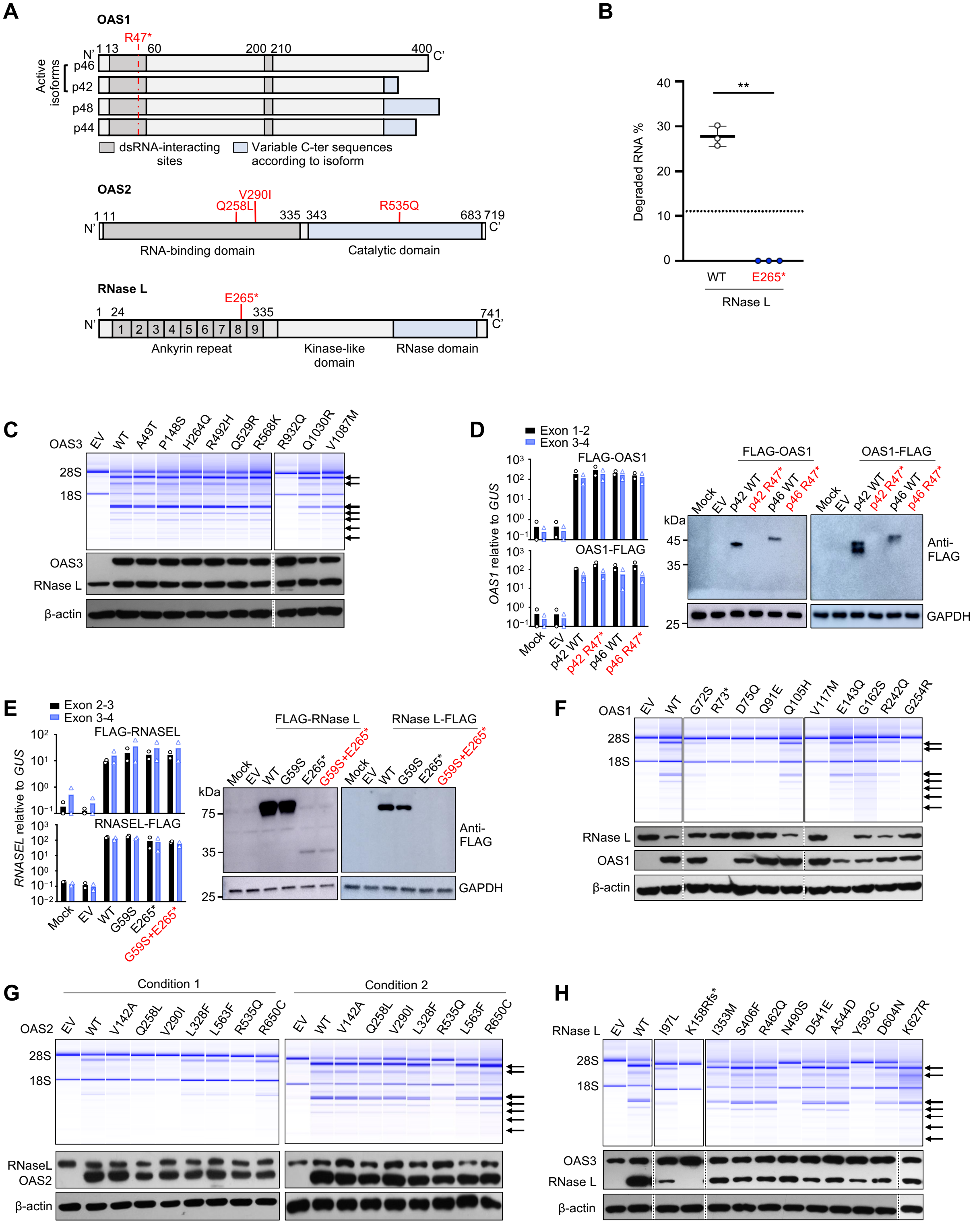
A
OAS1
R47*
Active isoforms
p46
p42
p48
p44
dsRNA-interacting sites
Variable C-ter sequences according to isoform
OAS2
V290I
Q258L
R535Q
RNA-binding domain
Catalytic domain
RNase L
E265*
Ankyrin repeat
Kinase-like domain
RNase domain
B
Degraded RNA %
WT
E265*
RNase L
C
OAS3
28S
18S
RNase L
β-actin
D
Exon 1-2
Exon 3-4
FLAG-OAS1
OAS1-FLAG
OAS1 relative to GUS
Anti-FLAG
GAPDH
E
Exon 2-3
Exon 3-4
FLAG-RNASEL
RNASEL-FLAG
RNASEL relative to GUS
FLAG-RNase L
RNase L-FLAG
F
OAS1
G
Condition 1
Condition 2
OAS2
RNaseL
H
RNase L
OAS3

Fig. S2. Enrichment in homozygous deleterious *OAS1*, *OAS2,* and *RNASEL* variants in MIS-C patients

(**A**) Schematic diagram of the structure of the OAS1, OAS2 and RNase L proteins and the location of deleterious mutations specific to the MIS-C patients. (**B**) Semi-quantification of the levels of rRNA degradation by WT or mutant (MT) *RNASEL* cDNA. Area under the curve (AUC) of the major degraded rRNA species peak as a percentage of the total AUC for 28S and 18S rRNA. The data shown are means ± SEM from three independent experiments with one technical replicate per experiment. Statistical tests were performed as described in the Methods. $^{**}P<0.01$. (**C**) Functional assays for WT and mutant OAS3. Variants potentially compound heterozygous in our MIS-C cohort were tested. Upper panels: RNase L-mediated cleavage of rRNA. Lower panels: immunoblots for the indicated proteins. (**D** and **E**) mRNA and protein levels for WT and MT OAS1 and RNase L in HEK293 cells following transient transfection with a plasmid encoding N-terminally or C-terminally Flag-tagged OAS1 (isoform p42 or p46) (**D**) or RNase L (**E**). (**F** to **H**) Functional assays for the various OAS1 (**F**), OAS2 (**G**), and RNase L (**H**) variants, and the corresponding WT gene products. Variants for which homozygotes are reported in gnomAD were tested. Upper panels: RNase L-mediated cleavage of rRNA. Lower panels: immunoblots for the indicated proteins. OAS2 variants (**G**) were tested under two different sets of conditions, as described in the Methods. The results shown in (**C**, **F**, and **G**) are representative of three independent experiments. The data points in (**D** and **E**) are means from two independent experiments with two technical replicates per experiment. EV: empty vector.

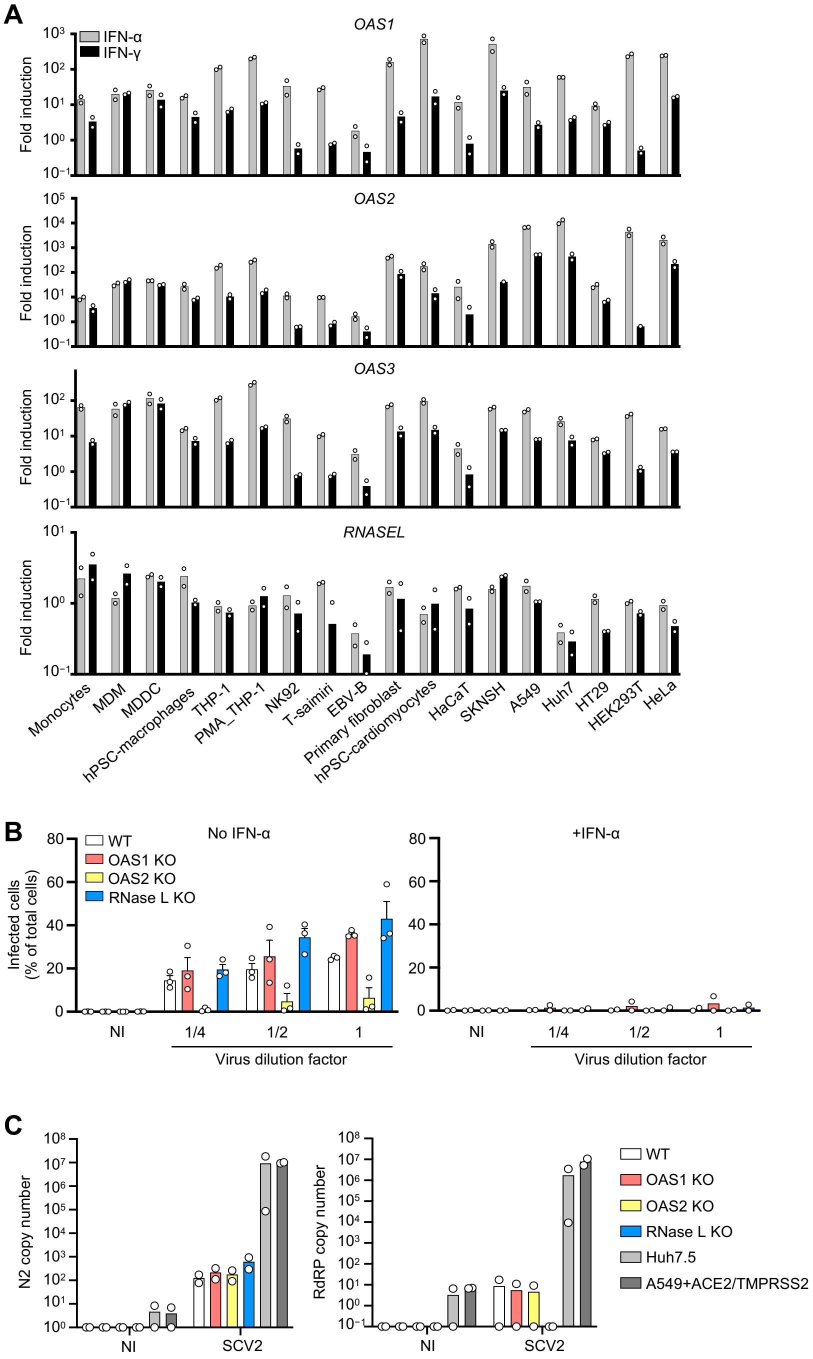
A
IFN-α
IFN-γ
OAS1
OAS2
OAS3
RNASEL
Fold induction
Monocytes
MDM
MDDC
hPSC-macrophages
THP-1
PMA_THP-1
NK92
T-saimiri
EBV-B
Primary fibroblast
hPSC-cardiomyocytes
HaCaT
SKNSH
A549
Huh7
HT29
HEK293T
HeLa
B
WT
OAS1 KO
OAS2 KO
RNase L KO
No IFN-α
+IFN-α
Infected cells
(% of total cells)
NI
1/4
1/2
1
Virus dilution factor
C
N2 copy number
RdRP copy number
NI
SCV2
WT
OAS1 KO
OAS2 KO
RNase L KO
Huh7.5
A549+ACE2/TMPRSS2

Fig. S3. Expression pattern of the OAS–RNase L pathway genes and their role in SARS-CoV-2 restriction

(**A**) *OAS1*, *OAS2*, *OAS3*, and *RNASEL* mRNA induction after 8 hours of stimulation with IFN-α2b or IFN-γ, as quantified by RT-qPCR. Each data point represents a technical duplicate. MDM: monocyte-derived macrophages, MDDC: monocyte-derived dendritic cells, hPSC: human pluripotent stem cells, PMA_THP-1: PMA-primed THP-1 cells. (**B**) Percentage of cells positive for SARS-CoV-2, as determined by measuring the immunofluorescence of SARS-CoV-2 N-protein in OAS1 KO, OAS2 KO, RNase L KO, or parental (WT) A549 +ACE2/TMPRSS2 cells, 48 hours after infection with various dilutions of SARS-CoV-2. Dilution factors 1/4, 1/2 and 1 correspond to a MOI of 0.0002, 0.0005 and 0.001, respectively. NI, noninfected. The data shown are means ± SEM from three independent experiments with three to six technical replicates per experiment. (**C**) SARS-CoV-2 N2 and RdRP RNA levels as measured by RT-qPCR in THP-1 cells, Huh7.5 cells, and A549+ACE2/TMPRSS2 cells infected with SARS-CoV-2 for 24 hours. The data points shown are biological replicates from one (A549+ACE2/TMPRSS2 cells) to two independent experiments (THP-1 cells and Huh7.5 cells).

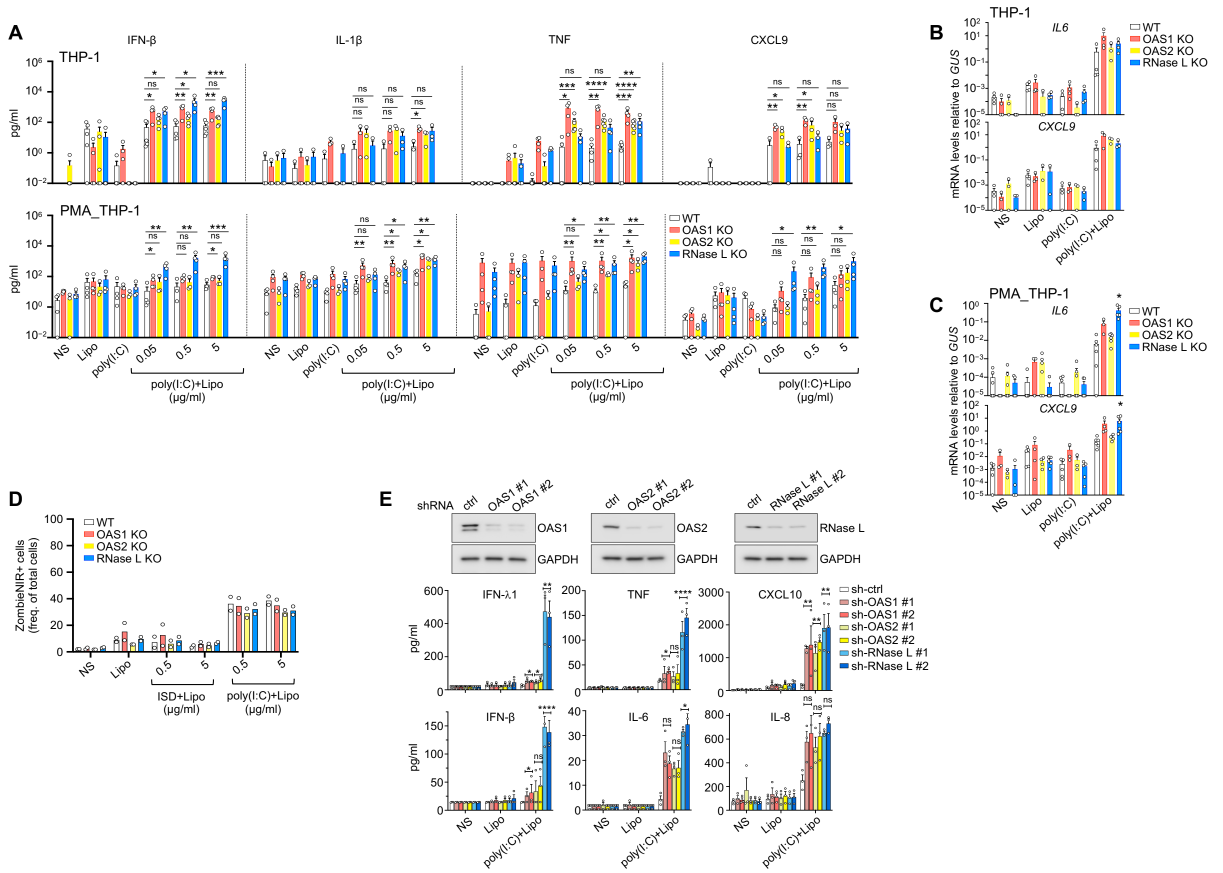

Fig. S4. Exaggerated inflammatory response of THP-1 cells with OAS–RNase L deficiency

(A) Concentrations of various cytokines in the supernatant of OAS1 KO, OAS2 KO, RNase L KO, or parental (WT) THP-1 cells (upper panel) or PMA-primed THP-1 cells (lower panel) treated as indicated for 24 hours. The data shown are means ± SEM from three to five independent experiments, with one to two technical replicates per experiment. **(B** to **C)** *IL6* and *CXCL9* relative mRNA levels as determined by RT-qPCR in OAS1 KO, OAS2 KO, RNase L KO, or parental (WT) THP-1 cells (**B**) or PMA-primed THP-1 cells (**C**). The data shown are means ± SEM from three to six independent experiments with two technical replicates per experiment. **(D)** Live–dead staining of THP-1 cells after stimulation with lipofectamine, lipofectamine with control nucleic acid (ISD), or lipofectamine with poly(I:C) in WT parental, OAS1 KO, OAS2 KO, and RNase L KO THP-1 cells. Cells were stained with ZombieNIR after 24 hours of stimulation and acquired by flow cytometry. The data shown are from two independent experiments with two technical replicates per experiment. **(E)** Concentrations of various cytokines in the supernatant of THP-1 cells transduced with a shRNA targeting *OAS1*, *OAS2*, *RNASEL* (OAS1 KDn, OAS2 KDn, RNase

L KDn), or a scrambled control shRNA (sh-ctrl), treated as indicated. The data shown are means ± SEM from three independent experiments with two technical replicates per experiment. At the top, immunoblots of THP-1 cells transduced with shRNA targeting *OAS1*, *OAS2*, *RNASEL* or sh-ctrl. GAPDH was used as a loading control. In (**A** to **C** and **E**), statistical tests were performed as described in the Methods. ns: not significant, *$P<0.05$, **$P<0.01$, ***$P<0.001$, ****$P<0.0001$. NS: nonstimulated; Lipo: lipofectamine only; poly(I:C): extracellularly added poly(I:C); poly(I:C)+Lipo: intracellular poly(I:C) in the presence of lipofectamine.

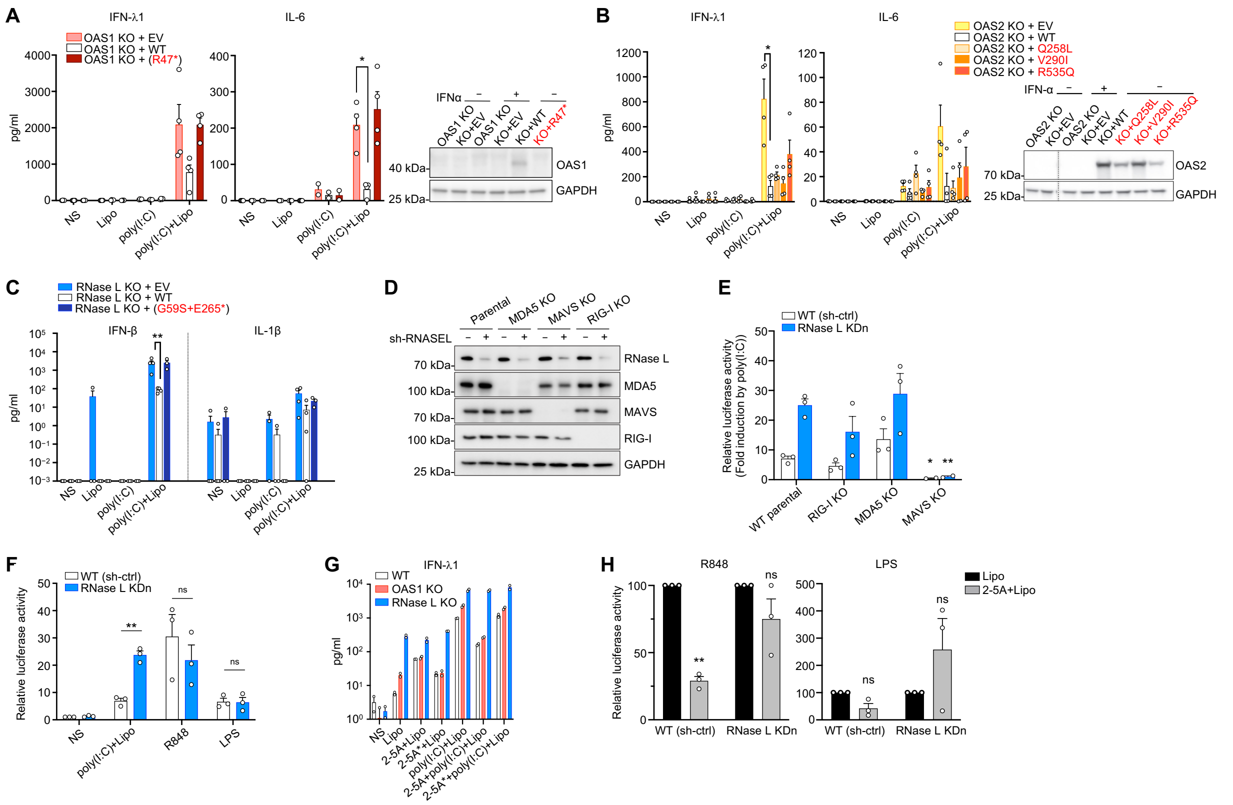

Fig. S5. Defective activation of the OAS–RNase L pathway results in an exaggerated inflammatory response of THP-1 cells upon intracellular dsRNA stimulation

(**A**) IFN-λ1 and IL-6 concentrations in the supernatant of OAS1 KO THP-1 cells transduced with the WT or P1's variant *OAS1* cDNA (R47*), or an empty vector (EV), and treated as indicated for 24 hours. The data shown are means ± SEM of four biological replicates from two independent experiments. (**B**) IFN-λ1 and IL-6 concentrations in the supernatant of OAS2 KO THP-1 cells transduced with the WT or the patient-specific variant *OAS2* cDNAs, or an EV, and treated as indicated for 24 hours. The data shown are means ± SEM from four biological replicates from two independent experiments. (**C**) IFN-β, IL-1β, and TNF concentrations in the supernatant of RNase L KO THP-1 cells transduced with the WT or P5's variant *RNASEL* cDNA, or an EV, treated as indicated for 24 hours. The data shown are means ± SEM from three to four independent experiments with one to two biological replicates per experiment. (**D**) Protein levels for RNase L, RIG-I, MDA5, and MAVS as assessed by immunoblotting on WT parental or RIG-I KO, MDA5 KO, or MAVS KO THP-1 cells, with or without RNase L knockdown (KDn). GAPDH was used

as a loading control. (**E**) Relative luciferase activity induced by intracellular poly(I:C) in parental, RIG-I KO, MDA5 KO, or MDA5 KO THP-1 cells expressing an ISRE-luciferase reporter, with or without RNase L KDn. Data are represented as a fold-change relative to cells stimulated with lipofectamine only. The data shown are means ± SEM from three independent experiments with two biological replicates per experiment. (**F**) Relative luciferase activity induced by intracellular poly(I:C), R848, or LPS in parental THP-1 cells expressing an ISRE-luciferase reporter, with or without RNase L KDn. Data are represented as activity relative to NS conditions for WT sh-ctrl. The data shown are means ± SEM from three independent experiments, with one to two technical replicates per experiment. (**G**) IFN-λ1 and IL-6 concentrations in the supernatant of parental, OAS1 KO, or RNase L KO THP-1 cells treated for 24 hours as indicated. Each data point represents a technical duplicate. (**H**) Relative luciferase activity induced by R848 or LPS in parental THP-1 cells with or without (WT sh-ctrl) RNase L KDn, in the presence of intracellular 2-5A or lipofectamine only. Data are expressed as a percentage of the value in lipofectamine-only conditions. The data shown are means ± SEM from three independent experiments with one to two technical replicates per experiment. In (**A** to **C**, **E** to **H**), statistical tests were performed as described in the Methods. ns: not significant, *$P<0.05$, **$P<0.01$. NS: nonstimulated; Lipo: lipofectamine only; poly(I:C): extracellularly added poly(I:C); poly(I:C)+Lipo: intracellular poly(I:C) in the presence of lipofectamine; 2-5A+Lipo: intracellular 2-5A in the presence of lipofectamine; 2-5A*+Lipo: intracellular dephosphorylated 2-5A in the presence of lipofectamine; 2-5A+poly(I:C)+Lipo or 2-5A*+poly(I:C)+Lipo: intracellular poly(I:C) in addition to intracellular 2-5A or 2-5A*.

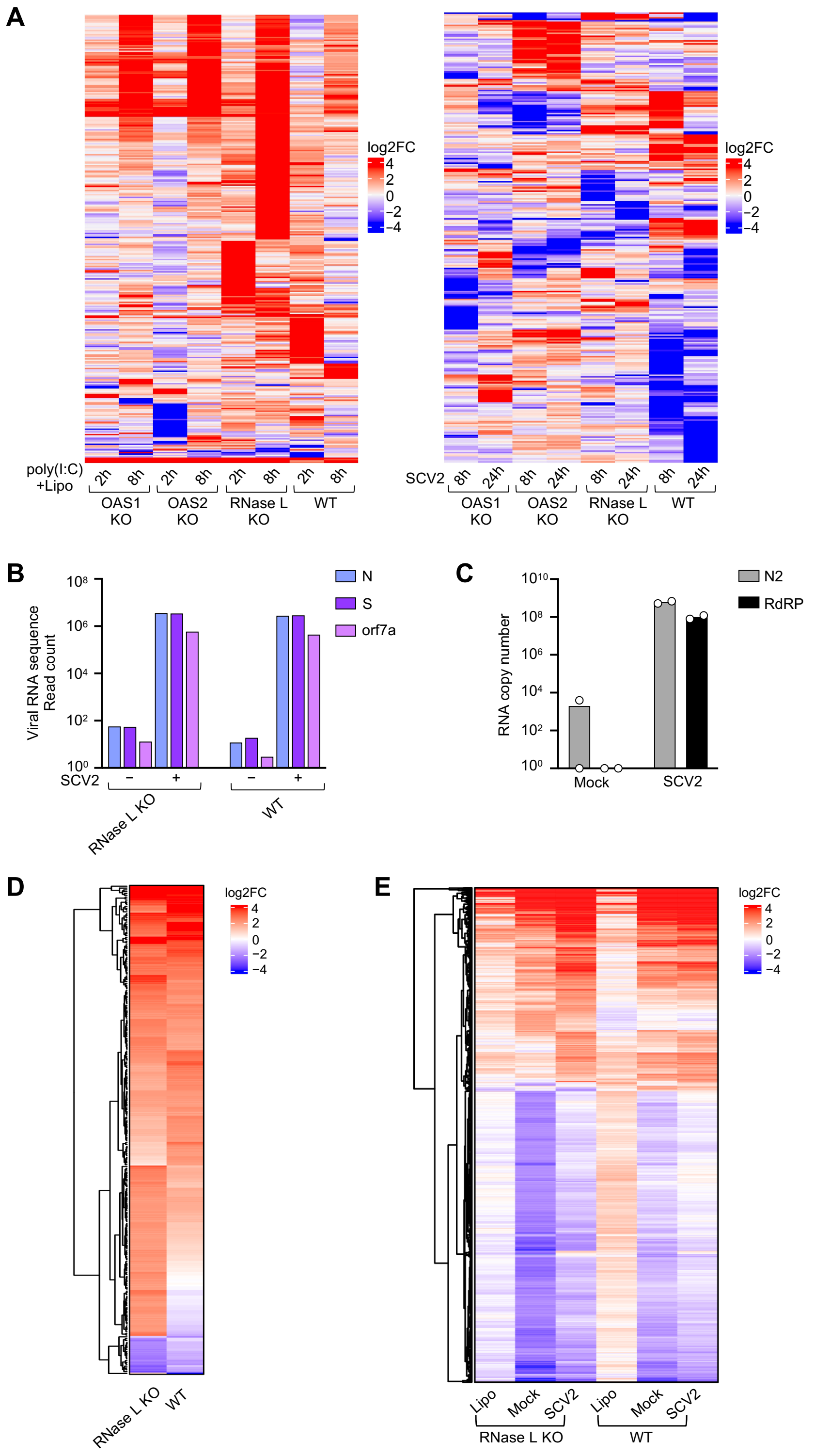

A
log2FC
4
2
0
−2
−4
poly(I:C)
+Lipo
2h
8h
OAS1 KO
OAS2 KO
RNase L KO
WT
SCV2
24h
B
Viral RNA sequence
Read count
N
S
orf7a
RNase L KO
WT
C
RNA copy number
N2
RdRP
Mock
SCV2
D
E
Lipo
Mock

Fig. S6. Exaggerated inflammatory responses to SARS-CoV-2 in THP-1 cells with OAS–RNase L deficiencies

(**A**) Heatmaps of RNAseq-quantified gene induction ($\log_2$FC) relative to nonstimulated conditions for OAS1 KO, OAS2 KO, RNase L KO, and parental THP-1 cells following stimulation with intracellular poly(I:C) for 2 or 8 hours (left), or live SARS-CoV-2 for 8 or 24 hours (right). (**B**) Detection of SCV2 viral RNA from bulk RNAseq data for total RNA from THP-1 and Vero cell coculture samples, with (SCV2) or without (Mock) SARS-CoV-2 infection for 24 hours. (**C**) SARS-CoV-2 N2 and RdRP RNA levels as measured by RT-qPCR in Vero cells infected with SARS-CoV-2 or mock-infected for 72 hours. Datapoints are means of technical duplicates. (**D**) Heatmaps of RNAseq-quantified gene induction ($\log_2$FC) relative to nonstimulated conditions, for RNase L KO and parental (WT) THP-1 cells cocultured for 24 hours with Vero cells infected for 48 hours with SARS-CoV-2 (SCV2) or mock-infected. (**E**) Heatmaps of RNAseq-quantified gene induction ($\log_2$FC) relative to nonstimulated conditions for RNase L KO and parental (WT) THP-1 cells transfected with total RNA extracted from SARS-CoV-2–infected (SCV2) or mock-infected (Mock) Vero cells, or treated with lipofectamine only (Lipo) for 8 hours.

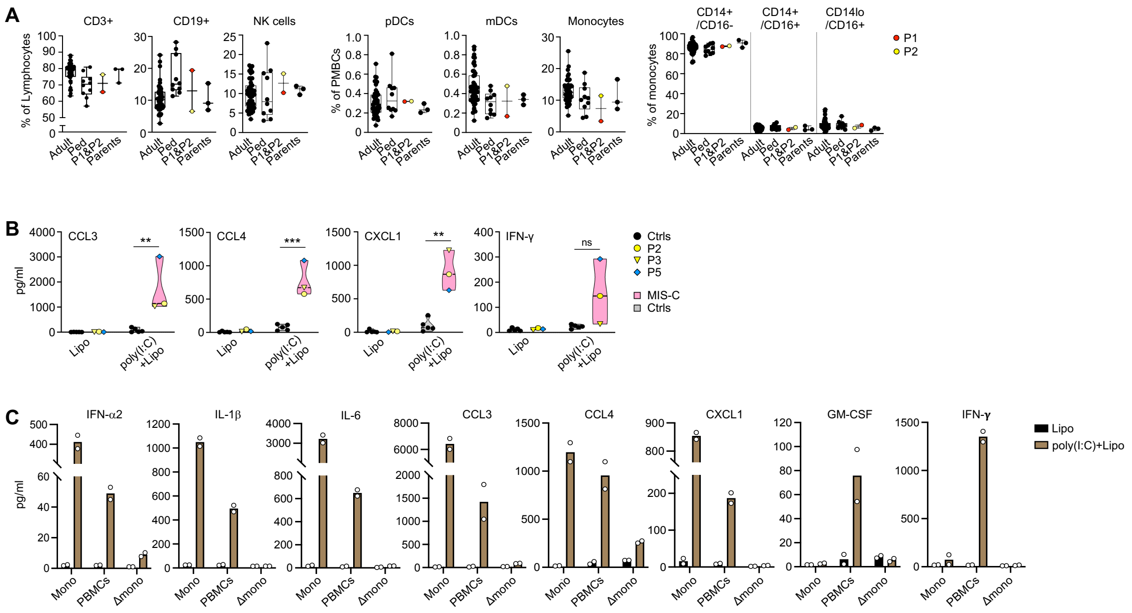

Fig. S7. Exaggerated myeloid cell activation in response to SARS-CoV-2 underlies MIS-C

(**A**) Blood leukocyte subpopulation analysis based on deep immunophenotyping by mass cytometry (CyTOF) for P1 (red) and P2 (yellow) relative to healthy adult (Adult) and pediatric controls (Ped), both parents of P1, and the mother of P2 (Parents). (**B**) Concentrations of various cytokines in the supernatant of PBMCs from OAS–RNase L-deficient patients (grouped in pink violin zone), and three healthy pediatric and two healthy adult controls (Ctrls) (gray violin zone), treated as indicated for 24 hours. Each data point represents the mean of biological duplicates. Statistical tests were performed to compare the patient group with the control group, as described in the Methods. ns: not significant. ***P*<0.01, ****P*<0.001. (**C**) Concentrations of various cytokines in the supernatant of monocytes (Mono), PBMCs, and monocyte-depleted PBMCs (Δmono) from two healthy controls treated as indicated for 24 hours. The data shown are from two independent experiments with two different donors, each tested with duplicates. Each data point represents the mean of biological duplicates for each donor. Lipo: lipofectamine only; poly(I:C)+Lipo: intracellular poly(I:C) in the presence of lipofectamine.

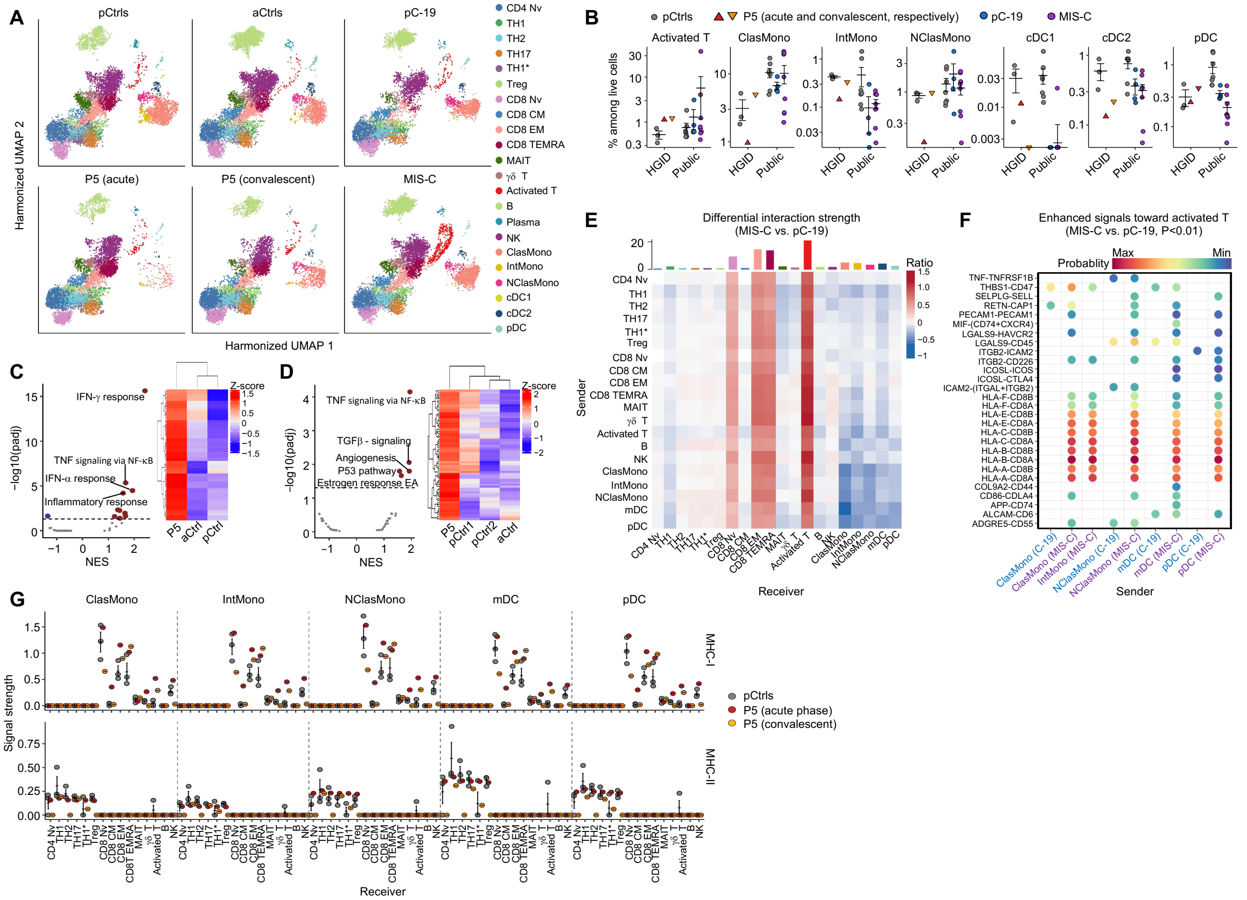

Fig. S8. Single-cell transcriptome analysis of a patient with MIS-C and inherited RNase L deficiency

(**A** to **F**) scRNAseq of cryopreserved PBMCs from P5 sampled during the acute and convalescent phases; comparison with cells from healthy adult and pediatric controls (aCtrls, pCtrls). A published dataset for pediatric patients with acute SARS-CoV-2 infection (pC-19) or MIS-C was integrated. (**A**) Cellular composition. After excluding irrelevant cell types, 10,000 cells were randomly sampled from each category for visual comparison. (**B**) Cell abundance. Data from our laboratory (HGID) are shown separately from the previously published data (Public). (**C** and **D**) Differential expression analysis and gene set enrichment analysis (GSEA) for genes with significantly higher or lower levels of expression in P5's nonclassical monocytes (**C**) or pDCs (**D**) sorted from her convalescent phase PBMCs, than in healthy adult and pediatric controls. Volcano plot: immune system-related pathways. NES: normalized enrichment score. Heatmaps: Gene expression (Z-score-scaled log_2 read counts per million, cpm) for the Hallmark "IFN-γ response" gene set. (**E** and **F**) Deconvolution of intercellular communications inferred with CellChat,

comparing MIS-C with pediatric acute SARS-CoV-2 infection (pC-19). (**G**) Signals relating to the MHC-I and MHC-II pathways are derived from myeloid cell subsets and directed toward lymphoid cell subsets.

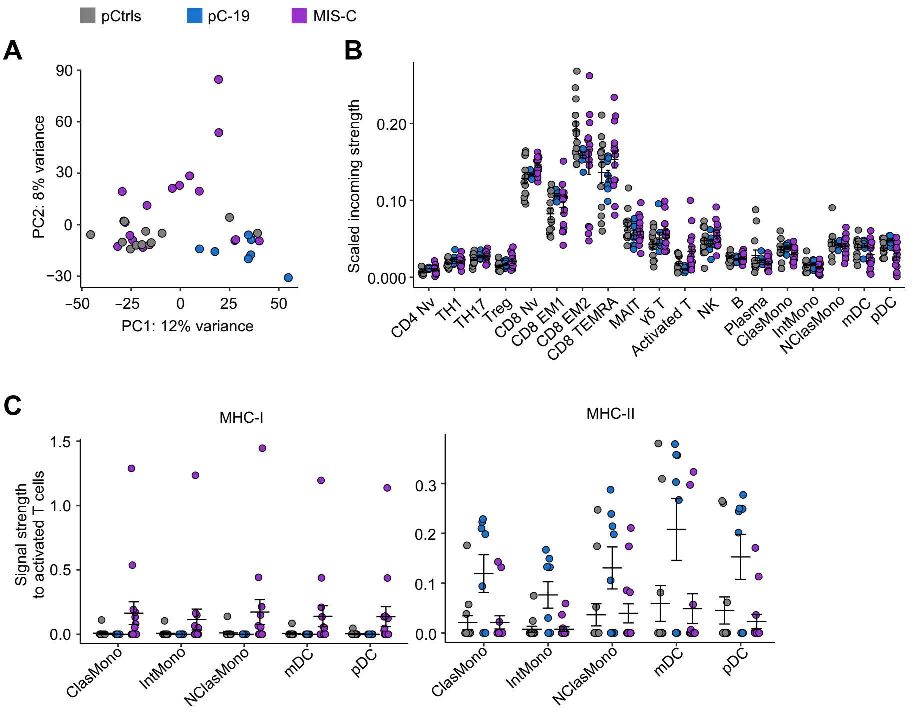

Fig. S9. Single-cell transcriptome analysis of public datasets for MIS-C patients and controls

(**A** to **C**) Intercellular communication analysis with CellChat was independently repeated with two other sets of scRNAseq data for healthy pediatric controls (pCtrls) and children with acute SARS-CoV-2 infection (pC-19) or MIS-C (GSE166489). (**A**) PCA plot of all predicted interactions between all sender–receiver pairs of cell subsets. (**B**) The incoming signal strength for representative cell subsets is shown. (**C**) Signals relating to the MHC-I and MHC-II pathways are derived from myeloid cell subsets and directed toward activated T cells.

Table S1.

Nonsynonymous variants of *OAS1*, *OAS2* and *RNASEL* with homozygous carriers reported in the gnomAD database

Gene	Nucleotide change	Amino acid change	MAF (gnomAD)	Number of homozygotes in gnomAD	CADD_Phred	Exp function
OAS1	c.225C>A	p.Asp75Glu (D75Q)	0.000011	1	7.19	LOF
OAS1	c.217C>T	p.Arg73* (R73*)	0.00005	1	32	LOF
OAS1	c.315G>C	p.Gln105His (Q105H)	0.000064	1	17.58	Isomorph
OAS1	c.428A>G	p.Glu143Gly (E143Q)	0.00071	1	16.98	Isomorph
OAS1	c.349G>A	p.Val117Met (V117M)	0.000131	1	21.6	LOF
OAS1	c.214G>A	p.Gly72Ser (G72S)	0.000195	1	22.3	Hypomorph
OAS1	c.760G>A	p.Gly254Arg (G254R)	0.000787	2	22.7	LOF
OAS1	c.271C>G	p.Gln91Glu (Q91E)	0.001149	3	19.16	LOF
OAS1	c.725G>A	p.Arg242Gln (R242Q)	0.005707	18	0.001	Isomorph
OAS1	c.484G>A	p.Gly162Ser (G162S)	0.562148	46873	3.09	Isomorph
OAS2	c.984A>C	p.Leu328Phe (L328F)	0.0000321	1	10.55	Hypomorph
OAS2	c.425T>C	p.Val142Ala (V142A)	0.0001676	1	15.85	Isomorph
OAS2	c.1948C>T	p.Arg650Cys (R650C)	0.0002653	1	25.1	Isomorph
OAS2	c.1689G>T	p.L563Phe (L563F)	0.0004657	2	24.4	Hypomorph
OAS2	c.868G>A	p.Val290Ile (V290I)	0.0005153	2	5.58	Hypomorph
RNASEL	c.1778A>G	p.Tyr593Cys (Y593C)	0.0000198	1	25.4	LOF
RNASEL	c.1810G>A	p.Asp604Asn (D604N)	0.0000672	1	25.7	Isomorph
RNASEL	c.1631C>A	p.Ala544Asp (A544D)	0.0000856	1	9.91	Isomorph
RNASEL	c.1469A>G	p.Asn490Ser (N490S)	0.0000916	1	25.4	LOF
RNASEL	c.1059A>G	p.Ile353Met (I353M)	0.0001995	1	0.002	Isomorph
RNASEL	c.1880A>G	p.Lys627Arg (K627R)	0.0004102	2	0.001	Isomorph
RNASEL	c.471_474delAAAG	p.Lys158ArgfsTer6 (K158Rfs*)	0.0004459	1	32	LOF
RNASEL	c.1217C>T	p.Ser406Phe (S406F)	0.0009347	2	22.6	Isomorph
RNASEL	c.175G>A	p.Gly59Ser (G59S)	0.0031	2	22.9	Isomorph
RNASEL	c.793G>T	p.Glu265* (E265*)	0.0031	2	33	LOF
RNASEL	c.289A>C	p.Ile97Leu (I97L)	0.0065401	14	22.6	Isomorph
RNASEL	c.1385G>A	p.Arg462Gln (R462Q)	0.3044157	14337	16.81	Isomorph
RNASEL	c.1623T>G	p.Asp541Glu (D541E)	0.5371442	41570	0.003	Isomorph

MAF, minor allele frequency.
Exp function, experimental function of each variant as tested in the RNase L-dependent rRNA degradation assay.

Table S2.

Characterization of the patient and control PBMC populations by CyTOF

Population	Adult controls	Pediatric controls	P1 (OAS1−/−)	P2 (OAS2-MT)	Parents
NK cells/Lymphocytes	9.965 (3.43, 17.2)	7.93 (3.06, 22.9)	10.2	15.1	11.2 (9.7, 11.9)
CD56bright/NK	5.155 (2.18, 17.7)	9.17 (2.16, 25.8)	6.81	3.57	4.62 (1.92, 8.91)
CD56dim/NK	94.85 (82.5, 97.8)	90.75 (74.2, 97.9)	93.1	96.4	95.4 (90.9, 98)
pDC/PBMCs	0.292 (0.072, 0.706)	0.3235 (0.165, 0.81)	0.321	0.318	0.233 (0.204, 0.297)
mDC/PBMCs	0.4295 (0.122, 0.883)	0.317 (0.149, 0.48)	0.167	0.479	0.34 (0.285, 0.389)
Monocytes/PBMCs	12.45 (5.17, 25.5)	10.4 (4.43, 18.7)	3.38	11.4	9.41 (7.23, 16.6)
CD14+CD16-/Monocytes	86.5 (71.3, 96.5)	84.15 (78, 90.7)	87.1	87.9	90.9 (86.1, 93.7)
CD14+CD16+/Monocytes	5.575 (1.22, 9.12)	6.23 (3.78, 11.1)	3.88	6.27	4.13 (3.43, 7.78)
CD14lowCD16+/Monocytes	7.055 (1.28, 24.1)	8.045 (3.93, 17.4)	8.51	5.43	4.83 (2.88, 5.88)
CD3+/Lymphocytes	79.2 (63.3, 88.1)	70.4 (57.1, 80.9)	65.7	76.3	79.6 (71.2, 79.7)
Naïve T cells/CD3+	35.75 (11.8, 56.9)	61 (51.9, 84.4)	69.5	48.9	26.7 (21.2, 39.4)
γδ+/Total naïve T cells	0.5305 (0.0269, 2.08)	1.43 (0.664, 5.21)	1.67	1.67	0.605 (0.439, 0.955)
CD4-CD8-/γδ- naïve T cells	0.5835 (0.199, 1.49)	0.921 (0.541, 1.41)	1.76	1.01	0.98 (0.702, 1.15)
Memory T cells/CD3+	65.3 (44.8, 89.3)	40 (17.9, 50.3)	33.1	53.6	73 (62.1, 81.2)
γδ+/Total memory T cells	7.295 (0.996, 19.4)	24.95 (17.5, 35.1)	22.3	13.2	4.73 (4.45, 9.66)
CD4-CD8-/γδ- memory T cells	1.26 (0.353, 6.69)	3.695 (2.02, 4.56)	4.34	3.15	2.11 (1.22, 2.55)
γδ+/CD3+	5.435 (0.67, 14.7)	11.35 (3.33, 16.1)	6.98	8.11	3.77 (3.1, 6.73)
γδ-CD4-CD8-/CD3+	1.055 (0.33, 4.35)	5.27 (0.906, 11.8)	2.74	23.4	2.36 (1.63, 9.98)
CD4+CD8-/CD3+	58.95 (41.8, 81.2)	55.25 (42.6, 71.5)	55.4	55.1	58.5 (40.3, 62.8)
Th1/Memory CD4 T cells	26.05 (14.5, 53.6)	22.25 (18.2, 26.5)	24	26.8	20.6 (16.2, 21.7)
Th1*/Memory CD4 T cells	25.2 (8.64, 47.6)	16.8 (5.48, 28.3)	9.29	30.2	32.4 (29.6, 36.7)
Th17/Memory CD4 T cells	10.18 (3.7, 19.2)	10.65 (8.1, 18.1)	8.36	7.59	9.49 (7.08, 14.3)
Th2/Memory CD4 T cells	7.615 (2.7, 18.1)	10.08 (5.26, 16.7)	18.1	5.01	3.62 (3.59, 7.39)
Tfh/Memory CD4 T cells	20.15 (11.2, 36.5)	23.15 (17.4, 28.6)	19.2	21.7	24 (21.9, 30.7)
Treg/CD4+CD8-	6.73 (4.75, 10.9)	8.615 (4.64, 9.74)	8.02	7.26	9.37 (6.49, 10.7)
Naïve/CD4+CD8-	44.6 (14.8, 68.9)	71.25 (54.7, 84.2)	85.5	54.6	39 (22.8, 46.5)
Central memory/CD4+CD8-	40.2 (20.4, 67.1)	21.7 (13.6, 32.4)	9.75	32.8	49.1 (38.7, 61.9)
Effector memory/CD4+CD8-	12.9 (5.44, 33.7)	6.835 (1.95, 12.4)	3.52	11.8	13.4 (10.2, 14.2)
TEMRA/CD4+CD8-	0.7525 (0.0886, 26.9)	0.3515 (0.116, 0.638)	1.19	0.74	1.42 (1.01, 1.65)
CD4-CD8-/CD3+	32.2 (16.5, 46.7)	29.65 (18.5, 40.7)	33.4	33.8	35.1 (30.8, 47.9)
Naïve T/CD4-CD8+	33.05 (4.77, 67.8)	70.6 (51.9, 87.5)	54.8	44	24.2 (19.1, 34.9)
Central memory/CD4-CD8+	15.85 (7.06, 40.1)	8.16 (4.89, 11.7)	3.88	13.9	17.8 (12.1, 21.3)
Effector memory/CD4-CD8+	20.85 (4.16, 51.4)	10.67 (2.28, 27.7)	5.43	21.1	21 (19.1, 26.2)
TEMRA/CD4-CD8+	28.1 (2.82, 75.6)	8.98 (4.77, 19)	36	21.2	38.7 (27, 38.9)
CD19+/Lymphocytes	10.2 (2.74, 24.2)	15.05 (11.3, 28.2)	19.4	6.54	9.09 (6.97, 15.3)
Naïve B cells/CD19+	72.45 (11.3, 85.7)	85.25 (66.4, 92.3)	89.5	67.5	69.8 (56.2, 83.1)
Transitional B cells/CD19+	3.415 (0.275, 16.1)	6.945 (3.76, 30.6)	13.7	7.02	4.43 (2, 13.5)
Memory B cells/CD19+	25.95 (11.2, 87.8)	13.45 (6.16, 32.9)	9.97	31.8	29.2 (16.2, 43.4)
Plasmablasts/CD19+	0.711 (0.143, 4.17)	1.395 (0.111, 2.12)	0.432	0.564	0.37 (0.0642, 0.698)

Note: Values are percentages and presented as median (min, max) unless otherwise specified.
Adult controls: n= 48; pediatric controls: n = 10.

Table S3.

Antibodies used for mass cytometry on fresh whole blood

Metal	Target	Titration	Clone	Manufacturer	Catalog
**163Dy**	CXCR3	1/800	G025H7	Fluidigm	3163004B
**152Sm**	TCRgd	1/800	11F2	Fluidigm	3152008B
**142Nd**	CD19	1/400	HIB19	Fluidigm	3142001B
**144Nd**	CD38	1/400	HIT2	Fluidigm	3144014B
**151Eu**	CD123	1/400	6H6	Fluidigm	3151001B
**153Eu**	Va7.2	1/400	3C10	Fluidigm	3153024B
**154Sm**	CD3	1/400	UCHT1	Fluidigm	3154003B
**155Gd**	CD45RA	1/400	HI100	Fluidigm	3155011B
**158Gd**	CD27	1/400	L128	Fluidigm	3158010B
**159Tb**	CD1c	1/400	L161	Biolegend	331502
**161Dy**	CLEC9A	1/400	8F9	Fluidigm	3161018B
**164Dy**	CD161	1/400	HP-3G10	Fluidigm	3164009B
**168Er**	CD8	1/400	SK1	Fluidigm	3168002B
**170Er**	iNKT	1/400	6B11	Fluidigm	3170015B
**175Lu**	CCR4	1/400	L291H4	Fluidigm	3175035A
**174Yb**	CD4	1/400	RPA-T4	Biolegend	300502
**162Dy**	CD21	1/400	REA940	Miltenyi Biotec Inc.	130-124-315
**165Ho**	NKG2C	1/400	REA205	Miltenyi Biotec Inc.	130-122-278
**148Nd**	CD20	1/200	2H7	Biolegend	302302
**173Yb**	HLA-DR	1/200	L243	Fluidigm	3173005B
**156Gd**	CCR10	1/200	REA326	Miltenyi Biotec Inc.	130-122-317
**089Y**	CD45	1/200	HI30	Fluidigm	3089003B
**116Cd**	CD66b	1/200	QA17A51	Biolegend	396902
**141Pr**	CCR6	1/200	G034E3	Fluidigm	3141003A
**143Nd**	CD127	1/200	A019D5	Fluidigm	3143012B
**147Sm**	CD11c	1/200	Bu15	Fluidigm	3147008B
**149Sm**	CD25	1/200	2A3	Fluidigm	3149010B
**150Nd**	NKVFS1	1/200	NKVFS1	Bio Rad	MCA2243GA
**167Er**	CCR7	1/200	G043H7	Fluidigm	3167009A
**169Tm**	NKG2A	1/200	Z199	Fluidigm	3169013B
**171Yb**	CXCR5	1/200	RF8B2	Fluidigm	3171014B
**166Er**	CD24	1/100	ML5	Fluidigm	3166007B
**145Nd**	CD31	1/100	WM59	Fluidigm	3145004B
**160Gd**	CD14	1/100	M5E2	Fluidigm	3160001B
**176Yb**	CD56	1/100	NCAM16.2	Fluidigm	3176008B
**172Yb**	CD57	1/100	HNK-1	Biolegend	359602
**150Nd**	KIR3DL1L2	1/100	REA970	Miltenyi Biotec Inc.	130-126-489
**146Nd**	IgD	1/50	IA6-2	Fluidigm	3146005B
**209Bi**	CD16	1/50	3G8	Fluidigm	3209002B

Titration represents the final dilution of the original antibody used in our experiments.

Data S1. SARS-CoV-2–induced immune responses across all PBMC cell types

Data S2. OAS–RNase L-deficient myeloid cells display enhanced pro-inflammatory responses to SARS-CoV-2

References and Notes

1. A. T. Levin, W. P. Hanage, N. Owusu-Boaitey, K. B. Cochran, S. P. Walsh, G. Meyerowitz-Katz, Assessing the age specificity of infection fatality rates for COVID-19: Systematic review, meta-analysis, and public policy implications. *Eur. J. Epidemiol.* **35**, 1123–1138 (2020). doi:10.1007/s10654-020-00698-1 Medline

2. M. O'Driscoll, G. Ribeiro Dos Santos, L. Wang, D. A. T. Cummings, A. S. Azman, J. Paireau, A. Fontanet, S. Cauchemez, H. Salje, Age-specific mortality and immunity patterns of SARS-CoV-2. *Nature* **590**, 140–145 (2021). doi:10.1038/s41586-020-2918-0 Medline

3. K. Bhaskaran, S. Bacon, S. J. W. Evans, C. J. Bates, C. T. Rentsch, B. MacKenna, L. Tomlinson, A. J. Walker, A. Schultze, C. E. Morton, D. Grint, A. Mehrkar, R. M. Eggo, P. Inglesby, I. J. Douglas, H. I. McDonald, J. Cockburn, E. J. Williamson, D. Evans, H. J. Curtis, W. J. Hulme, J. Parry, F. Hester, S. Harper, D. Spiegelhalter, L. Smeeth, B. Goldacre, Factors associated with deaths due to COVID-19 versus other causes: Population-based cohort analysis of UK primary care data and linked national death registrations within the OpenSAFELY platform. *Lancet Reg. Health Eur.* **6**, 100109 (2021). doi:10.1016/j.lanepe.2021.100109 Medline

4. E. J. Williamson, A. J. Walker, K. Bhaskaran, S. Bacon, C. Bates, C. E. Morton, H. J. Curtis, A. Mehrkar, D. Evans, P. Inglesby, J. Cockburn, H. I. McDonald, B. MacKenna, L. Tomlinson, I. J. Douglas, C. T. Rentsch, R. Mathur, A. Y. S. Wong, R. Grieve, D. Harrison, H. Forbes, A. Schultze, R. Croker, J. Parry, F. Hester, S. Harper, R. Perera, S. J. W. Evans, L. Smeeth, B. Goldacre, Factors associated with COVID-19-related death using OpenSAFELY. *Nature* **584**, 430–436 (2020). doi:10.1038/s41586-020-2521-4 Medline

5. Q. Zhang, P. Bastard, Z. Liu, J. Le Pen, M. Moncada-Velez, J. Chen, M. Ogishi, I. K. D. Sabli, S. Hodeib, C. Korol, J. Rosain, K. Bilguvar, J. Ye, A. Bolze, B. Bigio, R. Yang, A. A. Arias, Q. Zhou, Y. Zhang, F. Onodi, S. Korniotis, L. Karpf, Q. Philippot, M. Chbihi, L. Bonnet-Madin, K. Dorgham, N. Smith, W. M. Schneider, B. S. Razooky, H.-H. Hoffmann, E. Michailidis, L. Moens, J. E. Han, L. Lorenzo, L. Bizien, P. Meade, A.-L. Neehus, A. C. Ugurbil, A. Corneau, G. Kerner, P. Zhang, F. Rapaport, Y. Seeleuthner, J. Manry, C. Masson, Y. Schmitt, A. Schlüter, T. Le Voyer, T. Khan, J. Li, J. Fellay, L. Roussel, M. Shahrooei, M. F. Alosaimi, D. Mansouri, H. Al-Saud, F. Al-Mulla, F. Almourfi, S. Z. Al-Muhsen, F. Alsohime, S. Al Turki, R. Hasanato, D. van de Beek, A. Biondi, L. R. Bettini, M. D'Angio', P. Bonfanti, L. Imberti, A. Sottini, S. Paghera, E. Quiros-Roldan, C. Rossi, A. J. Oler, M. F. Tompkins, C. Alba, I. Vandernoot, J.-C. Goffard, G. Smits, I. Migeotte, F. Haerynck, P. Soler-Palacin, A. Martin-Nalda, R. Colobran, P.-E. Morange, S. Keles, F. Çölkesen, T. Ozcelik, K. K. Yasar, S. Senoglu, Ş. N. Karabela, C. Rodríguez-Gallego, G. Novelli, S. Hraiech, Y. Tandjaoui-Lambiotte, X. Duval, C. Laouénan, A. L. Snow, C. L. Dalgard, J. D. Milner, D. C. Vinh, T. H. Mogensen, N. Marr, A. N. Spaan, B. Boisson, S. Boisson-Dupuis, J. Bustamante, A. Puel, M. J. Ciancanelli, I. Meyts, T. Maniatis, V. Soumelis, A. Amara, M. Nussenzweig, A. García-Sastre, F. Krammer, A. Pujol, D. Duffy, R. P. Lifton, S.-Y. Zhang, G. Gorochov, V. Béziat, E. Jouanguy, V. Sancho-Shimizu, C. M. Rice, L. Abel, L. D. Notarangelo, A. Cobat, H. C. Su, J.-L. Casanova; COVID-STORM Clinicians; COVID Clinicians; Imagine COVID Group; French COVID Cohort Study Group; CoV-Contact

Cohort; Amsterdam UMC Covid-19 Biobank; COVID Human Genetic Effort; NIAID-USUHS/TAGC COVID Immunity Group, Inborn errors of type I IFN immunity in patients with life-threatening COVID-19. *Science* **370**, eabd4570 (2020). doi:10.1126/science.abd4570 Medline

6. P. Bastard, L. B. Rosen, Q. Zhang, E. Michailidis, H.-H. Hoffmann, Y. Zhang, K. Dorgham, Q. Philippot, J. Rosain, V. Béziat, J. Manry, E. Shaw, L. Haljasmägi, P. Peterson, L. Lorenzo, L. Bizien, S. Trouillet-Assant, K. Dobbs, A. A. de Jesus, A. Belot, A. Kallaste, E. Catherinot, Y. Tandjaoui-Lambiotte, J. Le Pen, G. Kerner, B. Bigio, Y. Seeleuthner, R. Yang, A. Bolze, A. N. Spaan, O. M. Delmonte, M. S. Abers, A. Aiuti, G. Casari, V. Lampasona, L. Piemonti, F. Ciceri, K. Bilguvar, R. P. Lifton, M. Vasse, D. M. Smadja, M. Migaud, J. Hadjadj, B. Terrier, D. Duffy, L. Quintana-Murci, D. van de Beek, L. Roussel, D. C. Vinh, S. G. Tangye, F. Haerynck, D. Dalmau, J. Martinez-Picado, P. Brodin, M. C. Nussenzweig, S. Boisson-Dupuis, C. Rodríguez-Gallego, G. Vogt, T. H. Mogensen, A. J. Oler, J. Gu, P. D. Burbelo, J. I. Cohen, A. Biondi, L. R. Bettini, M. D'Angio, P. Bonfanti, P. Rossignol, J. Mayaux, F. Rieux-Laucat, E. S. Husebye, F. Fusco, M. V. Ursini, L. Imberti, A. Sottini, S. Paghera, E. Quiros-Roldan, C. Rossi, R. Castagnoli, D. Montagna, A. Licari, G. L. Marseglia, X. Duval, J. Ghosn, J. S. Tsang, R. Goldbach-Mansky, K. Kisand, M. S. Lionakis, A. Puel, S.-Y. Zhang, S. M. Holland, G. Gorochov, E. Jouanguy, C. M. Rice, A. Cobat, L. D. Notarangelo, L. Abel, H. C. Su, J.-L. Casanova; HGID Lab; NIAID-USUHS Immune Response to COVID Group; COVID Clinicians; COVID-STORM Clinicians; Imagine COVID Group; French COVID Cohort Study Group; Milieu Intérieur Consortium; CoV-Contact Cohort; Amsterdam UMC Covid-19 Biobank; COVID Human Genetic Effort, Autoantibodies against type I IFNs in patients with life-threatening COVID-19. *Science* **370**, eabd4585 (2020). doi:10.1126/science.abd4585 Medline

7. T. Asano, B. Boisson, F. Onodi, D. Matuozzo, M. Moncada-Velez, M. R. L. Maglorius Renkilaraj, P. Zhang, L. Meertens, A. Bolze, M. Materna, S. Korniotis, A. Gervais, E. Talouarn, B. Bigio, Y. Seeleuthner, K. Bilguvar, Y. Zhang, A.-L. Neehus, M. Ogishi, S. J. Pelham, T. Le Voyer, J. Rosain, Q. Philippot, P. Soler-Palacín, R. Colobran, A. Martin-Nalda, J. G. Rivière, Y. Tandjaoui-Lambiotte, K. Chaïbi, M. Shahrooei, I. A. Darazam, N. A. Olyaei, D. Mansouri, N. Hatipoğlu, F. Palabiyik, T. Ozcelik, G. Novelli, A. Novelli, G. Casari, A. Aiuti, P. Carrera, S. Bondesan, F. Barzaghi, P. Rovere-Querini, C. Tresoldi, J. L. Franco, J. Rojas, L. F. Reyes, I. G. Bustos, A. A. Arias, G. Morelle, K. Christèle, J. Troya, L. Planas-Serra, A. Schlüter, M. Gut, A. Pujol, L. M. Allende, C. Rodriguez-Gallego, C. Flores, O. Cabrera-Marante, D. E. Pleguezuelo, R. P. de Diego, S. Keles, G. Aytekin, O. M. Akcan, Y. T. Bryceson, P. Bergman, P. Brodin, D. Smole, C. I. E. Smith, A.-C. Norlin, T. M. Campbell, L. E. Covill, L. Hammarström, Q. Pan-Hammarström, H. Abolhassani, S. Mane, N. Marr, M. Ata, F. Al Ali, T. Khan, A. N. Spaan, C. L. Dalgard, P. Bonfanti, A. Biondi, S. Tubiana, C. Burdet, R. Nussbaum, A. Kahn-Kirby, A. L. Snow, J. Bustamante, A. Puel, S. Boisson-Dupuis, S.-Y. Zhang, V. Béziat, R. P. Lifton, P. Bastard, L. D. Notarangelo, L. Abel, H. C. Su, E. Jouanguy, A. Amara, V. Soumelis, A. Cobat, Q. Zhang, J.-L. Casanova; COVID Human Genetic Effort; COVID-STORM Clinicians; COVID Clinicians; Imagine COVID Group; French COVID Cohort Study Group; CoV-Contact Cohort; Amsterdam UMC Covid-; Biobank; NIAID-USUHS COVID Study Group, X-linked recessive TLR7 deficiency in ~1% of

men under 60 years old with life-threatening COVID-19. *Sci. Immunol.* **6**, eabl4348 (2021). doi:10.1126/sciimmunol.abl4348 Medline

8. P. Bastard, A. Gervais, T. Le Voyer, J. Rosain, Q. Philippot, J. Manry, E. Michailidis, H.-H. Hoffmann, S. Eto, M. Garcia-Prat, L. Bizien, A. Parra-Martínez, R. Yang, L. Haljasmägi, M. Migaud, K. Särekannu, J. Maslovskaja, N. de Prost, Y. Tandjaoui-Lambiotte, C.-E. Luyt, B. Amador-Borrero, A. Gaudet, J. Poissy, P. Morel, P. Richard, F. Cognasse, J. Troya, S. Trouillet-Assant, A. Belot, K. Saker, P. Garçon, J. G. Rivière, J.-C. Lagier, S. Gentile, L. B. Rosen, E. Shaw, T. Morio, J. Tanaka, D. Dalmau, P.-L. Tharaux, D. Sene, A. Stepanian, B. Megarbane, V. Triantafyllia, A. Fekkar, J. R. Heath, J. L. Franco, J.-M. Anaya, J. Solé-Violán, L. Imberti, A. Biondi, P. Bonfanti, R. Castagnoli, O. M. Delmonte, Y. Zhang, A. L. Snow, S. M. Holland, C. Biggs, M. Moncada-Vélez, A. A. Arias, L. Lorenzo, S. Boucherit, B. Coulibaly, D. Anglicheau, A. M. Planas, F. Haerynck, S. Duvlis, R. L. Nussbaum, T. Ozcelik, S. Keles, A. A. Bousfiha, J. El Bakkouri, C. Ramirez-Santana, S. Paul, Q. Pan-Hammarström, L. Hammarström, A. Dupont, A. Kurolap, C. N. Metz, A. Aiuti, G. Casari, V. Lampasona, F. Ciceri, L. A. Barreiros, E. Dominguez-Garrido, M. Vidigal, M. Zatz, D. van de Beek, S. Sahanic, I. Tancevski, Y. Stepanovskyy, O. Boyarchuk, Y. Nukui, M. Tsumura, L. Vidaur, S. G. Tangye, S. Burrel, D. Duffy, L. Quintana-Murci, A. Klocperk, N. Y. Kann, A. Shcherbina, Y.-L. Lau, D. Leung, M. Coulongeat, J. Marlet, R. Koning, L. F. Reyes, A. Chauvineau-Grenier, F. Venet, G. Monneret, M. C. Nussenzweig, R. Arrestier, I. Boudhabhay, H. Baris-Feldman, D. Hagin, J. Wauters, I. Meyts, A. H. Dyer, S. P. Kennelly, N. M. Bourke, R. Halwani, N. S. Sharif-Askari, K. Dorgham, J. Sallette, S. M. Sedkaoui, S. AlKhater, R. Rigo-Bonnin, F. Morandeira, L. Roussel, D. C. Vinh, S. R. Ostrowski, A. Condino-Neto, C. Prando, A. Bonradenko, A. N. Spaan, L. Gilardin, J. Fellay, S. Lyonnet, K. Bilguvar, R. P. Lifton, S. Mane, M. S. Anderson, B. Boisson, V. Béziat, S.-Y. Zhang, E. Vandreakos, O. Hermine, A. Pujol, P. Peterson, T. H. Mogensen, L. Rowen, J. Mond, S. Debette, X. de Lamballerie, X. Duval, F. Mentré, M. Zins, P. Soler-Palacin, R. Colobran, G. Gorochov, X. Solanich, S. Susen, J. Martinez-Picado, D. Raoult, M. Vasse, P. K. Gregersen, L. Piemonti, C. Rodríguez-Gallego, L. D. Notarangelo, H. C. Su, K. Kisand, S. Okada, A. Puel, E. Jouanguy, C. M. Rice, P. Tiberghien, Q. Zhang, A. Cobat, L. Abel, J.-L. Casanova; HGID Lab; COVID Clinicians; COVID-STORM Clinicians; NIAID Immune Response to COVID Group; NH-COVAIR Study Group; Danish CHGE; Danish Blood Donor Study; St. James's Hospital; SARS CoV2 Interest group; French COVID Cohort Study Group; Imagine COVID-Group; Milieu Intérieur Consortium; CoV-Contact Cohort; Amsterdam UMC Covid-19; Biobank Investigators; COVID Human Genetic Effort; CONSTANCES cohort; 3C-Dijon Study; Cerba Health-Care; Etablissement du Sang study group, Autoantibodies neutralizing type I IFNs are present in ~4% of uninfected individuals over 70 years old and account for ~20% of COVID-19 deaths. *Sci. Immunol.* **6**, eabl4340 (2021). doi:10.1126/sciimmunol.abl4340 Medline

9. Q. Zhang, D. Matuozzo, J. Le Pen, D. Lee, L. Moens, T. Asano, J. Bohlen, Z. Liu, M. Moncada-Velez, Y. Kendir-Demirkol, H. Jing, L. Bizien, A. Marchal, H. Abolhassani, S. Delafontaine, G. Bucciol, G. I. Bayhan, S. Keles, A. Kiykim, S. Hancerli, F. Haerynck, B. Florkin, N. Hatipoglu, T. Ozcelik, G. Morelle, M. Zatz, L. F. P. Ng, D. C. Lye, B. E. Young, Y. S. Leo, C. L. Dalgard, R. P. Lifton, L. Renia, I. Meyts, E. Jouanguy, L. Hammarström, Q. Pan-Hammarström, B. Boisson, P. Bastard, H. C. Su, S. Boisson-Dupuis, L. Abel, C. M. Rice, S. Y. Zhang, A. Cobat, J. L. Casanova; COVID Human

Genetic Effort, Recessive inborn errors of type I IFN immunity in children with COVID-19 pneumonia. *J. Exp. Med.* **219**, e20220131 (2022). doi:10.1084/jem.20220131 Medline

10. E. Pairo-Castineira, S. Clohisey, L. Klaric, A. D. Bretherick, K. Rawlik, D. Pasko, S. Walker, N. Parkinson, M. H. Fourman, C. D. Russell, J. Furniss, A. Richmond, E. Gountouna, N. Wrobel, D. Harrison, B. Wang, Y. Wu, A. Meynert, F. Griffiths, W. Oosthuyzen, A. Kousathanas, L. Moutsianas, Z. Yang, R. Zhai, C. Zheng, G. Grimes, R. Beale, J. Millar, B. Shih, S. Keating, M. Zechner, C. Haley, D. J. Porteous, C. Hayward, J. Yang, J. Knight, C. Summers, M. Shankar-Hari, P. Klenerman, L. Turtle, A. Ho, S. C. Moore, C. Hinds, P. Horby, A. Nichol, D. Maslove, L. Ling, D. McAuley, H. Montgomery, T. Walsh, A. C. Pereira, A. Renieri, X. Shen, C. P. Ponting, A. Fawkes, A. Tenesa, M. Caulfield, R. Scott, K. Rowan, L. Murphy, P. J. M. Openshaw, M. G. Semple, A. Law, V. Vitart, J. F. Wilson, J. K. Baillie; GenOMICC Investigators; ISARIC4C Investigators; COVID-19 Human Genetics Initiative; 23andMe Investigators; BRACOVID Investigators; Gen-COVID Investigators, Genetic mechanisms of critical illness in COVID-19. *Nature* **591**, 92–98 (2021). doi:10.1038/s41586-020-03065-y Medline

11. H. Zeberg, S. Pääbo, The major genetic risk factor for severe COVID-19 is inherited from Neanderthals. *Nature* **587**, 610–612 (2020). doi:10.1038/s41586-020-2818-3 Medline

12. A. Kousathanas, E. Pairo-Castineira, K. Rawlik, A. Stuckey, C. A. Odhams, S. Walker, C. D. Russell, T. Malinauskas, Y. Wu, J. Millar, X. Shen, K. S. Elliott, F. Griffiths, W. Oosthuyzen, K. Morrice, S. Keating, B. Wang, D. Rhodes, L. Klaric, M. Zechner, N. Parkinson, A. Siddiq, P. Goddard, S. Donovan, D. Maslove, A. Nichol, M. G. Semple, T. Zainy, F. Maleady-Crowe, L. Todd, S. Salehi, J. Knight, G. Elgar, G. Chan, P. Arumugam, C. Patch, A. Rendon, D. Bentley, C. Kingsley, J. A. Kosmicki, J. E. Horowitz, A. Baras, G. R. Abecasis, M. A. R. Ferreira, A. Justice, T. Mirshahi, M. Oetjens, D. J. Rader, M. D. Ritchie, A. Verma, T. A. Fowler, M. Shankar-Hari, C. Summers, C. Hinds, P. Horby, L. Ling, D. McAuley, H. Montgomery, P. J. M. Openshaw, P. Elliott, T. Walsh, A. Tenesa, A. Fawkes, L. Murphy, K. Rowan, C. P. Ponting, V. Vitart, J. F. Wilson, J. Yang, A. D. Bretherick, R. H. Scott, S. C. Hendry, L. Moutsianas, A. Law, M. J. Caulfield, J. K. Baillie; GenOMICC investigators; 23andMe investigators; COVID-19 Human Genetics Initiative, Whole-genome sequencing reveals host factors underlying critical COVID-19. *Nature* **607**, 97–103 (2022). doi:10.1038/s41586-022-04576-6 Medline

13. COVID-19 Host Genetics Initiative, Mapping the human genetic architecture of COVID-19. *Nature* **600**, 472–477 (2021). doi:10.1038/s41586-021-03767-x Medline

14. S. B. Morris, N. G. Schwartz, P. Patel, L. Abbo, L. Beauchamps, S. Balan, E. H. Lee, R. Paneth-Pollak, A. Geevarughese, M. K. Lash, M. S. Dorsinville, V. Ballen, D. P. Eiras, C. Newton-Cheh, E. Smith, S. Robinson, P. Stogsdill, S. Lim, S. E. Fox, G. Richardson, J. Hand, N. T. Oliver, A. Kofman, B. Bryant, Z. Ende, D. Datta, E. Belay, S. Godfred-Cato, Case series of multisystem inflammatory syndrome in adults associated with SARS-CoV-2 infection — United Kingdom and United States, March–August 2020. *MMWR Morb. Mortal. Wkly. Rep.* **69**, 1450–1456 (2020). doi:10.15585/mmwr.mm6940e1 Medline

15. V. Sancho-Shimizu, P. Brodin, A. Cobat, C. M. Biggs, J. Toubiana, C. L. Lucas, S. E. Henrickson, A. Belot, S. G. Tangye, J. D. Milner, M. Levin, L. Abel, D. Bogunovic, J. L.

Casanova, S. Y. Zhang; MIS-C@CHGE, SARS-CoV-2–related MIS-C: A key to the viral and genetic causes of Kawasaki disease? *J. Exp. Med.* **218**, e20210446 (2021). doi:10.1084/jem.20210446 Medline

16. E. Whittaker, A. Bamford, J. Kenny, M. Kaforou, C. E. Jones, P. Shah, P. Ramnarayan, A. Fraisse, O. Miller, P. Davies, F. Kucera, J. Brierley, M. McDougall, M. Carter, A. Tremoulet, C. Shimizu, J. Herberg, J. C. Burns, H. Lyall, M. Levin; PIMS-TS Study Group and EUCLIDS and PERFORM Consortia, Clinical characteristics of 58 children with a pediatric inflammatory multisystem syndrome temporally associated with SARS-CoV-2. *JAMA* **324**, 259–269 (2020). doi:10.1001/jama.2020.10369 Medline

17. M. Ahmed, S. Advani, A. Moreira, S. Zoretic, J. Martinez, K. Chorath, S. Acosta, R. Naqvi, F. Burmeister-Morton, F. Burmeister, A. Tarriela, M. Petershack, M. Evans, A. Hoang, K. Rajasekaran, S. Ahuja, A. Moreira, Multisystem inflammatory syndrome in children: A systematic review. *EClinicalMedicine* **26**, 100527 (2020). doi:10.1016/j.eclinm.2020.100527 Medline

18. E. M. Dufort, E. H. Koumans, E. J. Chow, E. M. Rosenthal, A. Muse, J. Rowlands, M. A. Barranco, A. M. Maxted, E. S. Rosenberg, D. Easton, T. Udo, J. Kumar, W. Pulver, L. Smith, B. Hutton, D. Blog, H. Zucker; New York State and Centers for Disease Control and Prevention Multisystem Inflammatory Syndrome in Children Investigation Team, Multisystem inflammatory syndrome in children in New York State. *N. Engl. J. Med.* **383**, 347–358 (2020). doi:10.1056/NEJMoa2021756 Medline

19. A. B. Payne, Z. Gilani, S. Godfred-Cato, E. D. Belay, L. R. Feldstein, M. M. Patel, A. G. Randolph, M. Newhams, D. Thomas, R. Magleby, K. Hsu, M. Burns, E. Dufort, A. Maxted, M. Pietrowski, A. Longenberger, S. Bidol, J. Henderson, L. Sosa, A. Edmundson, M. Tobin-D’Angelo, L. Edison, S. Heidemann, A. R. Singh, J. S. Giuliano Jr., L. C. Kleinman, K. M. Tarquinio, R. F. Walsh, J. C. Fitzgerald, K. N. Clouser, S. J. Gertz, R. W. Carroll, C. L. Carroll, B. E. Hoots, C. Reed, F. S. Dahlgren, M. E. Oster, T. J. Pierce, A. T. Curns, G. E. Langley, A. P. Campbell, N. Balachandran, T. S. Murray, C. Burkholder, T. Brancard, J. Lifshitz, D. Leach, I. Charpie, C. Tice, S. E. Coffin, D. Perella, K. Jones, K. L. Marohn, P. H. Yager, N. D. Fernandes, H. R. Flori, M. L. Koncicki, K. S. Walker, M. C. Di Pentima, S. Li, S. M. Horwitz, S. Gaur, D. C. Coffey, I. Harwayne-Gidansky, S. R. Hymes, N. J. Thomas, K. G. Ackerman, J. M. Cholette; MIS-C Incidence Authorship Group, Incidence of multisystem inflammatory syndrome in children among US persons infected with SARS-CoV-2. *JAMA Netw. Open* **4**, e2116420 (2021). doi:10.1001/jamanetworkopen.2021.16420 Medline

20. L. R. Feldstein, M. W. Tenforde, K. G. Friedman, M. Newhams, E. B. Rose, H. Dapul, V. L. Soma, A. B. Maddux, P. M. Mourani, C. Bowens, M. Maamari, M. W. Hall, B. J. Riggs, J. S. Giuliano Jr., A. R. Singh, S. Li, M. Kong, J. E. Schuster, G. E. McLaughlin, S. P. Schwartz, T. C. Walker, L. L. Loftis, C. V. Hobbs, N. B. Halasa, S. Doymaz, C. J. Babbitt, J. R. Hume, S. J. Gertz, K. Irby, K. N. Clouser, N. Z. Cvijanovich, T. T. Bradford, L. S. Smith, S. M. Heidemann, S. P. Zackai, K. Wellnitz, R. A. Nofziger, S. M. Horwitz, R. W. Carroll, C. M. Rowan, K. M. Tarquinio, E. H. Mack, J. C. Fitzgerald, B. M. Coates, A. M. Jackson, C. C. Young, M. B. F. Son, M. M. Patel, J. W. Newburger, A. G. Randolph; Overcoming COVID-19 Investigators, Characteristics and outcomes of US children and adolescents with multisystem inflammatory syndrome in children (MIS-C)

compared with severe acute COVID-19. *JAMA* **325**, 1074–1087 (2021). doi:10.1001/jama.2021.2091 Medline

21. M. J. Carter, M. Fish, A. Jennings, K. J. Doores, P. Wellman, J. Seow, S. Acors, C. Graham, E. Timms, J. Kenny, S. Neil, M. H. Malim, S. M. Tibby, M. Shankar-Hari, Peripheral immunophenotypes in children with multisystem inflammatory syndrome associated with SARS-CoV-2 infection. *Nat. Med.* **26**, 1701–1707 (2020). doi:10.1038/s41591-020-1054-6 Medline

22. C. R. Consiglio, N. Cotugno, F. Sardh, C. Pou, D. Amodio, L. Rodriguez, Z. Tan, S. Zicari, A. Ruggiero, G. R. Pascucci, V. Santilli, T. Campbell, Y. Bryceson, D. Eriksson, J. Wang, A. Marchesi, T. Lakshmikanth, A. Campana, A. Villani, P. Rossi, N. Landegren, P. Palma, P. Brodin; CACTUS Study Team, The immunology of multisystem inflammatory syndrome in children with COVID-19. *Cell* **183**, 968–981.e7 (2020). doi:10.1016/j.cell.2020.09.016 Medline

23. L. Hoste, R. Van Paemel, F. Haerynck, Multisystem inflammatory syndrome in children related to COVID-19: A systematic review. *Eur. J. Pediatr.* **180**, 2019–2034 (2021). doi:10.1007/s00431-021-03993-5 Medline

24. J. Toubiana, J. F. Cohen, J. Brice, C. Poirault, F. Bajolle, W. Curtis, F. Moulin, S. Matczak, M. Leruez, J.-L. Casanova, M. Chalumeau, M. Taylor, S. Allali, Distinctive features of Kawasaki disease following SARS-CoV-2 infection: a controlled study in Paris, France. *J. Clin. Immunol.* **41**, 526–535 (2021). doi:10.1007/s10875-020-00941-0 Medline

25. B. Cherqaoui, I. Koné-Paut, H. Yager, F. L. Bourgeois, M. Piram, Delineating phenotypes of Kawasaki disease and SARS-CoV-2-related inflammatory multisystem syndrome: A French study and literature review. *Rheumatology* **60**, 4530–4537 (2021). doi:10.1093/rheumatology/keab026 Medline

26. C. N. Gruber, R. S. Patel, R. Trachtman, L. Lepow, F. Amanat, F. Krammer, K. M. Wilson, K. Onel, D. Geanon, K. Tuballes, M. Patel, K. Mouskas, T. O'Donnell, E. Merritt, N. W. Simons, V. Barcessat, D. M. Del Valle, S. Udondem, G. Kang, S. Gangadharan, G. Ofori-Amanfo, U. Laserson, A. Rahman, S. Kim-Schulze, A. W. Charney, S. Gnjatic, B. D. Gelb, M. Merad, D. Bogunovic, Mapping systemic inflammation and antibody responses in multisystem inflammatory syndrome in children (MIS-C). *Cell* **183**, 982–995.e14 (2020). doi:10.1016/j.cell.2020.09.034 Medline

27. C. Diorio, S. E. Henrickson, L. A. Vella, K. O. McNerney, J. Chase, C. Burudpakdee, J. H. Lee, C. Jasen, F. Balamuth, D. M. Barrett, B. L. Banwell, K. M. Bernt, A. M. Blatz, K. Chiotos, B. T. Fisher, J. C. Fitzgerald, J. S. Gerber, K. Gollomp, C. Gray, S. A. Grupp, R. M. Harris, T. J. Kilbaugh, A. R. O. John, M. Lambert, E. J. Liebling, M. E. Paessler, W. Petrosa, C. Phillips, A. F. Reilly, N. D. Romberg, A. Seif, D. A. Sesok-Pizzini, K. E. Sullivan, J. Vardaro, E. M. Behrens, D. T. Teachey, H. Bassiri, Multisystem inflammatory syndrome in children and COVID-19 are distinct presentations of SARS-CoV-2. *J. Clin. Invest.* **130**, 5967–5975 (2020). doi:10.1172/JCI140970 Medline

28. P. Y. Lee, M. Day-Lewis, L. A. Henderson, K. G. Friedman, J. Lo, J. E. Roberts, M. S. Lo, C. D. Platt, J. Chou, K. J. Hoyt, A. L. Baker, T. M. Banzon, M. H. Chang, E. Cohen, S. D. de Ferranti, A. Dionne, S. Habiballah, O. Halyabar, J. S. Hausmann, M. M. Hazen, E. Janssen, E. Meidan, R. W. Nelson, A. A. Nguyen, R. P. Sundel, F. Dedeoglu, P. A.

Nigrovic, J. W. Newburger, M. B. F. Son, Distinct clinical and immunological features of SARS-CoV-2-induced multisystem inflammatory syndrome in children. *J. Clin. Invest.* **130**, 5942–5950 (2020). doi:10.1172/JCI141113 Medline

29. A. Esteve-Sole, J. Anton, R. M. Pino-Ramirez, J. Sanchez-Manubens, V. Fumadó, C. Fortuny, M. Rios-Barnes, J. Sanchez-de-Toledo, M. Girona-Alarcón, J. M. Mosquera, S. Ricart, C. Launes, M. F. de Sevilla, C. Jou, C. Muñoz-Almagro, E. González-Roca, A. Vergara, J. Carrillo, M. Juan, D. Cuadras, A. Noguera-Julian, I. Jordan, L. Alsina, Similarities and differences between the immunopathogenesis of COVID-19-related pediatric multisystem inflammatory syndrome and Kawasaki disease. *J. Clin. Invest.* **131**, e144554 (2021). doi:10.1172/JCI144554 Medline

30. H. Bukulmez, Current understanding of multisystem inflammatory syndrome (MIS-C) following COVID-19 and its distinction from Kawasaki disease. *Curr. Rheumatol. Rep.* **23**, 58 (2021). doi:10.1007/s11926-021-01028-4 Medline

31. L. A. Vella, J. R. Giles, A. E. Baxter, D. A. Oldridge, C. Diorio, L. Kuri-Cervantes, C. Alanio, M. B. Pampena, J. E. Wu, Z. Chen, Y. J. Huang, E. M. Anderson, S. Gouma, K. O. McNerney, J. Chase, C. Burudpakdee, J. H. Lee, S. A. Apostolidis, A. C. Huang, D. Mathew, O. Kuthuru, E. C. Goodwin, M. E. Weirick, M. J. Bolton, C. P. Arevalo, A. Ramos, C. J. Jasen, P. E. Conrey, S. Sayed, H. M. Giannini, K. D'Andrea, N. J. Meyer, E. M. Behrens, H. Bassiri, S. E. Hensley, S. E. Henrickson, D. T. Teachey, M. R. Betts, E. J. Wherry; UPenn COVID Processing Unit, Deep immune profiling of MIS-C demonstrates marked but transient immune activation compared to adult and pediatric COVID-19. *Sci. Immunol.* **6**, eabf7570 (2021). doi:10.1126/sciimmunol.abf7570 Medline

32. M. Moreews, K. Le Gouge, S. Khaldi-Plassart, R. Pescarmona, A.-L. Mathieu, C. Malcus, S. Djebali, A. Bellomo, O. Dauwalder, M. Perret, M. Villard, E. Chopin, I. Rouvet, F. Vandenesh, C. Dupieux, R. Pouyau, S. Teyssedre, M. Guerder, T. Louazon, A. Moulin-Zinsch, M. Duperril, H. Patural, L. Giovannini-Chami, A. Portefaix, B. Kassai, F. Venet, G. Monneret, C. Lombard, H. Flodrops, J.-M. De Guillebon, F. Bajolle, V. Launay, P. Bastard, S.-Y. Zhang, V. Dubois, O. Thaunat, J.-C. Richard, M. Mezidi, O. Allatif, K. Saker, M. Dreux, L. Abel, J.-L. Casanova, J. Marvel, S. Trouillet-Assant, D. Klatzmann, T. Walzer, E. Mariotti-Ferrandiz, E. Javouhey, A. Belot, Polyclonal expansion of TCR Vβ 21.3$^+$ CD4$^+$ and CD8$^+$ T cells is a hallmark of multisystem inflammatory syndrome in children. *Sci. Immunol.* **6**, eabh1516 (2021). doi:10.1126/sciimmunol.abh1516 Medline

33. C. de Cevins, M. Luka, N. Smith, S. Meynier, A. Magérus, F. Carbone, V. García-Paredes, L. Barnabei, M. Batignes, A. Boullé, M. C. Stolzenberg, B. P. Pérot, B. Charbit, T. Fali, V. Pirabakaran, B. Sorin, Q. Riller, G. Abdessalem, M. Beretta, L. Grzelak, P. Goncalves, J. P. Di Santo, H. Mouquet, O. Schwartz, M. Zarhrate, M. Parisot, C. Bole-Feysot, C. Masson, N. Cagnard, A. Corneau, C. Brunaud, S. Y. Zhang, J. L. Casanova, B. Bader-Meunier, J. Haroche, I. Melki, M. Lorrot, M. Oualha, F. Moulin, D. Bonnet, Z. Belhadjer, M. Leruez, S. Allali, C. Gras-Leguen, L. de Pontual, A. Fischer, D. Duffy, F. Rieux-Laucat, J. Toubiana, M. M. Ménager; Pediatric-Biocovid Study Group, A monocyte/dendritic cell molecular signature of SARS-CoV-2-related multisystem inflammatory syndrome in children with severe myocarditis. *Med (N Y)* **2**, 1072–1092.e7 (2021). doi:10.1016/j.medj.2021.08.002 Medline

34. A. Ramaswamy, N. N. Brodsky, T. S. Sumida, M. Comi, H. Asashima, K. B. Hoehn, N. Li, Y. Liu, A. Shah, N. G. Ravindra, J. Bishai, A. Khan, W. Lau, B. Sellers, N. Bansal, P. Guerrerio, A. Unterman, V. Habet, A. J. Rice, J. Catanzaro, H. Chandnani, M. Lopez, N. Kaminski, C. S. Dela Cruz, J. S. Tsang, Z. Wang, X. Yan, S. H. Kleinstein, D. van Dijk, R. W. Pierce, D. A. Hafler, C. L. Lucas, Immune dysregulation and autoreactivity correlate with disease severity in SARS-CoV-2-associated multisystem inflammatory syndrome in children. *Immunity* **54**, 1083–1095.e7 (2021). doi:10.1016/j.immuni.2021.04.003 Medline

35. L. A. Vella, A. H. Rowley, Current insights into the pathophysiology of multisystem inflammatory syndrome in children. *Curr. Pediatr. Rep.* **9**, 83–92 (2021). doi:10.1007/s40124-021-00257-6 Medline

36. K. Sacco, R. Castagnoli, S. Vakkilainen, C. Liu, O. M. Delmonte, C. Oguz, I. M. Kaplan, S. Alehashemi, P. D. Burbelo, F. Bhuyan, A. A. de Jesus, K. Dobbs, L. B. Rosen, A. Cheng, E. Shaw, M. S. Vakkilainen, F. Pala, J. Lack, Y. Zhang, D. L. Fink, V. Oikonomou, A. L. Snow, C. L. Dalgard, J. Chen, B. A. Sellers, G. A. Montealegre Sanchez, K. Barron, E. Rey-Jurado, C. Vial, M. C. Poli, A. Licari, D. Montagna, G. L. Marseglia, F. Licciardi, U. Ramenghi, V. Discepolo, A. Lo Vecchio, A. Guarino, E. M. Eisenstein, L. Imberti, A. Sottini, A. Biondi, S. Mató, D. Gerstbacher, M. Truong, M. A. Stack, M. Magliocco, M. Bosticardo, T. Kawai, J. J. Danielson, T. Hulett, M. Askenazi, S. Hu, J. I. Cohen, H. C. Su, D. B. Kuhns, M. S. Lionakis, T. M. Snyder, S. M. Holland, R. Goldbach-Mansky, J. S. Tsang, L. D. Notarangelo; NIAID Immune Response to COVID Group; Chile MIS-C Group; Pavia Pediatric COVID-19 Group, Immunopathological signatures in multisystem inflammatory syndrome in children and pediatric COVID-19. *Nat. Med.* **28**, 1050–1062 (2022). doi:10.1038/s41591-022-01724-3 Medline

37. R. A. Porritt, L. Paschold, M. N. Rivas, M. H. Cheng, L. M. Yonker, H. Chandnani, M. Lopez, D. Simnica, C. Schultheiß, C. Santiskulvong, J. Van Eyk, J. K. McCormick, A. Fasano, I. Bahar, M. Binder, M. Arditi, HLA class I–associated expansion of TRBV11-2 T cells in multisystem inflammatory syndrome in children. *J. Clin. Invest.* **131**, e146614 (2021). doi:10.1172/JCI146614 Medline

38. L. Hoste, L. Roels, L. Naesens, V. Bosteels, S. Vanhee, S. Dupont, C. Bosteels, R. Browaeys, N. Vandamme, K. Verstaen, J. Roels, K. F. A. Van Damme, B. Maes, E. De Leeuw, J. Declercq, H. Aegerter, L. Seys, U. Smole, S. De Prijck, M. Vanheerswynghels, K. Claes, V. Debacker, G. Van Isterdael, L. Backers, K. B. M. Claes, P. Bastard, E. Jouanguy, S.-Y. Zhang, G. Mets, J. Dehoorne, K. Vandekerckhove, P. Schelstraete, J. Willems, P. Stordeur, S. Janssens, R. Beyaert, Y. Saeys, J. L. Casanova, B. N. Lambrecht, F. Haerynck, S. J. Tavernier; MIS-C Clinicians, TIM3^{+}*TRBV11-2* T cells and IFNγ signature in patrolling monocytes and CD16^{+} NK cells delineate MIS-C. *J. Exp. Med.* **219**, e20211381 (2022). doi:10.1084/jem.20211381 Medline

39. J. L. Casanova, L. Abel, Mechanisms of viral inflammation and disease in humans. *Science* **374**, 1080–1086 (2021). doi:10.1126/science.abj7965 Medline

40. S. Y. Zhang, Q. Zhang, J. L. Casanova, H. C. Su; COVID Team, Severe COVID-19 in the young and healthy: Monogenic inborn errors of immunity? *Nat. Rev. Immunol.* **20**, 455–456 (2020). doi:10.1038/s41577-020-0373-7 Medline

41. Y. Itan, L. Shang, B. Boisson, E. Patin, A. Bolze, M. Moncada-Vélez, E. Scott, M. J. Ciancanelli, F. G. Lafaille, J. G. Markle, R. Martinez-Barricarte, S. J. de Jong, X.-F. Kong, P. Nitschke, A. Belkadi, J. Bustamante, A. Puel, S. Boisson-Dupuis, P. D. Stenson, J. G. Gleeson, D. N. Cooper, L. Quintana-Murci, J.-M. Claverie, S.-Y. Zhang, L. Abel, J.-L. Casanova, The human gene damage index as a gene-level approach to prioritizing exome variants. *Proc. Natl. Acad. Sci. U.S.A.* **112**, 13615–13620 (2015). doi:10.1073/pnas.1518646112 Medline

42. S. L. Schwartz, G. L. Conn, RNA regulation of the antiviral protein 2′-5′-oligoadenylate synthetase. *Wiley Interdiscip. Rev. RNA* **10**, e1534 (2019). doi:10.1002/wrna.1534 Medline

43. B. Dong, R. H. Silverman, 2-5A-dependent RNase molecules dimerize during activation by 2-5A. *J. Biol. Chem.* **270**, 4133–4137 (1995). doi:10.1074/jbc.270.8.4133 Medline

44. M. Uhlén, L. Fagerberg, B. M. Hallström, C. Lindskog, P. Oksvold, A. Mardinoglu, Å. Sivertsson, C. Kampf, E. Sjöstedt, A. Asplund, I. Olsson, K. Edlund, E. Lundberg, S. Navani, C. A.-K. Szigyarto, J. Odeberg, D. Djureinovic, J. O. Takanen, S. Hober, T. Alm, P.-H. Edqvist, H. Berling, H. Tegel, J. Mulder, J. Rockberg, P. Nilsson, J. M. Schwenk, M. Hamsten, K. von Feilitzen, M. Forsberg, L. Persson, F. Johansson, M. Zwahlen, G. von Heijne, J. Nielsen, F. Pontén, Tissue-based map of the human proteome. *Science* **347**, 1260419 (2015). doi:10.1126/science.1260419 Medline

45. L. Zhao, L. D. Birdwell, A. Wu, R. Elliott, K. M. Rose, J. M. Phillips, Y. Li, J. Grinspan, R. H. Silverman, S. R. Weiss, Cell-type-specific activation of the oligoadenylate synthetase–RNase L pathway by a murine coronavirus. *J. Virol.* **87**, 8408–8418 (2013). doi:10.1128/JVI.00769-13 Medline

46. S. Banerjee, A. Chakrabarti, B. K. Jha, S. R. Weiss, R. H. Silverman, Cell-type-specific effects of RNase L on viral induction of beta interferon. *mBio* **5**, e00856-14 (2014). doi:10.1128/mBio.00856-14 Medline

47. F. Rapaport, B. Boisson, A. Gregor, V. Béziat, S. Boisson-Dupuis, J. Bustamante, E. Jouanguy, A. Puel, J. Rosain, Q. Zhang, S.-Y. Zhang, J. G. Gleeson, L. Quintana-Murci, J.-L. Casanova, L. Abel, E. Patin, Negative selection on human genes underlying inborn errors depends on disease outcome and both the mode and mechanism of inheritance. *Proc. Natl. Acad. Sci. U.S.A.* **118**, e2001248118 (2021). doi:10.1073/pnas.2001248118 Medline

48. B. Dong, M. Niwa, P. Walter, R. H. Silverman, Basis for regulated RNA cleavage by functional analysis of RNase L and Ire1p. *RNA* **7**, 361–373 (2001). doi:10.1017/S1355838201002230 Medline

49. R. H. Silverman, J. J. Skehel, T. C. James, D. H. Wreschner, I. M. Kerr, rRNA cleavage as an index of ppp(A2'p)nA activity in interferon-treated encephalomyocarditis virus-infected cells. *J. Virol.* **46**, 1051–1055 (1983). doi:10.1128/jvi.46.3.1051-1055.1983 Medline

50. D. H. Wreschner, T. C. James, R. H. Silverman, I. M. Kerr, Ribosomal RNA cleavage, nuclease activation and 2-5A (ppp(A2'p)$_n$A) in interferon-treated cells. *Nucleic Acids Res.* **9**, 1571–1581 (1981). doi:10.1093/nar/9.7.1571 Medline

51. Y. Xiang, Z. Wang, J. Murakami, S. Plummer, E. A. Klein, J. D. Carpten, J. M. Trent, W. B. Isaacs, G. Casey, R. H. Silverman, Effects of RNase L mutations associated with prostate cancer on apoptosis induced by 2′,5′-oligoadenylates. *Cancer Res.* **63**, 6795–6801 (2003). Medline

52. L. A. Henderson, S. W. Canna, K. G. Friedman, M. Gorelik, S. K. Lapidus, H. Bassiri, E. M. Behrens, K. F. Kernan, G. S. Schulert, P. Seo, M. B. F. Son, A. H. Tremoulet, C. VanderPluym, R. S. M. Yeung, A. S. Mudano, A. S. Turner, D. R. Karp, J. J. Mehta, American College of Rheumatology clinical guidance for multisystem inflammatory syndrome in children associated with SARS-CoV-2 and hyperinflammation in pediatric COVID-19: version 3. *Arthritis Rheumatol.* **74**, e1–e20 (2022). doi:10.1002/art.42062 Medline

53. A. Wickenhagen, E. Sugrue, S. Lytras, S. Kuchi, M. Noerenberg, M. L. Turnbull, C. Loney, V. Herder, J. Allan, I. Jarmson, N. Cameron-Ruiz, M. Varjak, R. M. Pinto, J. Y. Lee, L. Iselin, N. Palmalux, D. G. Stewart, S. Swingler, E. J. D. Greenwood, T. W. M. Crozier, Q. Gu, E. L. Davies, S. Clohisey, B. Wang, F. Trindade Maranhão Costa, M. Freire Santana, L. C. de Lima Ferreira, L. Murphy, A. Fawkes, A. Meynert, G. Grimes, J. L. Da Silva Filho, M. Marti, J. Hughes, R. J. Stanton, E. C. Y. Wang, A. Ho, I. Davis, R. F. Jarrett, A. Castello, D. L. Robertson, M. G. Semple, P. J. M. Openshaw, M. Palmarini, P. J. Lehner, J. K. Baillie, S. J. Rihn, S. J. Wilson; ISARIC4C Investigators, A prenylated dsRNA sensor protects against severe COVID-19. *Science* **374**, eabj3624 (2021). doi:10.1126/science.abj3624 Medline

54. O. Danziger, R. S. Patel, E. J. DeGrace, M. R. Rosen, B. R. Rosenberg, Inducible CRISPR activation screen for interferon-stimulated genes identifies OAS1 as a SARS-CoV-2 restriction factor. *PLOS Pathog.* **18**, e1010464 (2022). doi:10.1371/journal.ppat.1010464 Medline

55. Y. Li, D. M. Renner, C. E. Comar, J. N. Whelan, H. M. Reyes, F. L. Cardenas-Diaz, R. Truitt, L. H. Tan, B. Dong, K. D. Alysandratos, J. Huang, J. N. Palmer, N. D. Adappa, M. A. Kohanski, D. N. Kotton, R. H. Silverman, W. Yang, E. E. Morrisey, N. A. Cohen, S. R. Weiss, SARS-CoV-2 induces double-stranded RNA-mediated innate immune responses in respiratory epithelial-derived cells and cardiomyocytes. *Proc. Natl. Acad. Sci. U.S.A.* **118**, e2022643118 (2021). doi:10.1073/pnas.2022643118 Medline

56. P. Bastard, J. Manry, J. Chen, J. Rosain, Y. Seeleuthner, O. AbuZaitun, L. Lorenzo, T. Khan, M. Hasek, N. Hernandez, B. Bigio, P. Zhang, R. Lévy, S. Shrot, E. J. G. Reino, Y.-S. Lee, S. Boucherit, M. Aubart, R. Gijsbers, V. Béziat, Z. Li, S. Pellegrini, F. Rozenberg, N. Marr, I. Meyts, B. Boisson, A. Cobat, J. Bustamante, Q. Zhang, E. Jouangy, L. Abel, R. Somech, J.-L. Casanova, S.-Y. Zhang, Herpes simplex encephalitis in a patient with a distinctive form of inherited IFNAR1 deficiency. *J. Clin. Invest.* **131**, e139980 (2021). doi:10.1172/JCI139980 Medline

57. X. Song, W. Hu, H. Yu, L. Zhao, Y. Zhao, X. Zhao, H.-H. Xue, Y. Zhao, Little to no expression of angiotensin-converting enzyme-2 on most human peripheral blood immune cells but highly expressed on tissue macrophages. *Cytometry A* 10.1002/cyto.a.24285 (2020). doi:10.1002/cyto.a.24285 Medline

58. T. S. Rodrigues, K. S. G. de Sá, A. Y. Ishimoto, A. Becerra, S. Oliveira, L. Almeida, A. V. Gonçalves, D. B. Perucello, W. A. Andrade, R. Castro, F. P. Veras, J. E. Toller-Kawahisa, D. C. Nascimento, M. H. F. de Lima, C. M. S. Silva, D. B. Caetite, R. B. Martins, I. A. Castro, M. C. Pontelli, F. C. de Barros, N. B. do Amaral, M. C. Giannini, L. P. Bonjorno, M. I. F. Lopes, R. C. Santana, F. C. Vilar, M. Auxiliadora-Martins, R. Luppino-Assad, S. C. L. de Almeida, F. R. de Oliveira, S. S. Batah, L. Siyuan, M. N. Benatti, T. M. Cunha, J. C. Alves-Filho, F. Q. Cunha, L. D. Cunha, F. G. Frantz, T. Kohlsdorf, A. T. Fabro, E. Arruda, R. D. R. de Oliveira, P. Louzada-Junior, D. S. Zamboni, Inflammasomes are activated in response to SARS-CoV-2 infection and are associated with COVID-19 severity in patients. *J. Exp. Med.* **218**, e20201707 (2021). doi:10.1084/jem.20201707 Medline

59. J. Zheng, Y. Wang, K. Li, D. K. Meyerholz, C. Allamargot, S. Perlman, Severe acute respiratory syndrome coronavirus 2–induced immune activation and death of monocyte-derived human macrophages and dendritic cells. *J. Infect. Dis.* **223**, 785–795 (2021). doi:10.1093/infdis/jiaa753 Medline

60. I. Gresser, F. Vignaux, F. Belardelli, M. G. Tovey, M. T. Maunoury, Injection of mice with antibody to mouse interferon alpha/beta decreases the level of 2′-5′ oligoadenylate synthetase in peritoneal macrophages. *J. Virol.* **53**, 221–227 (1985). doi:10.1128/jvi.53.1.221-227.1985 Medline

61. W. Chanput, J. J. Mes, H. J. Wichers, THP-1 cell line: An in vitro cell model for immune modulation approach. *Int. Immunopharmacol.* **23**, 37–45 (2014). doi:10.1016/j.intimp.2014.08.002 Medline

62. W. B. Lee, W. Y. Choi, D.-H. Lee, H. Shim, J. Kim-Ha, Y.-J. Kim, OAS1 and OAS3 negatively regulate the expression of chemokines and interferon-responsive genes in human macrophages. *BMB Rep.* **52**, 133–138 (2019). doi:10.5483/BMBRep.2019.52.2.129 Medline

63. T. Kawai, S. Akira, Innate immune recognition of viral infection. *Nat. Immunol.* **7**, 131–137 (2006). doi:10.1038/ni1303 Medline

64. A. Chakrabarti, B. K. Jha, R. H. Silverman, New insights into the role of RNase L in innate immunity. *J. Interferon Cytokine Res.* **31**, 49–57 (2011). doi:10.1089/jir.2010.0120 Medline

65. A. G. Hovanessian, J. Wood, E. Meurs, L. Montagnier, Increased nuclease activity in cells treated with pppA2′p5′A2′p5′ A. *Proc. Natl. Acad. Sci. U.S.A.* **76**, 3261–3265 (1979). doi:10.1073/pnas.76.7.3261 Medline

66. A. Zhou, B. A. Hassel, R. H. Silverman, Expression cloning of 2-5A-dependent RNAase: A uniquely regulated mediator of interferon action. *Cell* **72**, 753–765 (1993). doi:10.1016/0092-8674(93)90403-D Medline

67. K. Malathi, J. M. Paranjape, E. Bulanova, M. Shim, J. M. Guenther-Johnson, P. W. Faber, T. E. Eling, B. R. G. Williams, R. H. Silverman, A transcriptional signaling pathway in the IFN system mediated by 2′-5′-oligoadenylate activation of RNase L. *Proc. Natl. Acad. Sci. U.S.A.* **102**, 14533–14538 (2005). doi:10.1073/pnas.0507551102 Medline

68. J. M. Burke, S. L. Moon, T. Matheny, R. Parker, RNase L reprograms translation by widespread mRNA turnover escaped by antiviral mRNAs. *Mol. Cell* **75**, 1203–1217.e5 (2019). doi:10.1016/j.molcel.2019.07.029 Medline

69. M. Knight, P. J. Cayley, R. H. Silverman, D. H. Wreschner, C. S. Gilbert, R. E. Brown, I. M. Kerr, Radioimmune, radiobinding and HPLC analysis of 2-5A and related oligonucleotides from intact cells. *Nature* **288**, 189–192 (1980). doi:10.1038/288189a0 Medline

70. A. Asthana, C. Gaughan, B. Dong, S. R. Weiss, R. H. Silverman, Specificity and mechanism of coronavirus, rotavirus, and mammalian two-histidine phosphoesterases that antagonize antiviral innate immunity. *mBio* **12**, e0178121 (2021). doi:10.1128/mBio.01781-21 Medline

71. A. Liberzon, C. Birger, H. Thorvaldsdóttir, M. Ghandi, J. P. Mesirov, P. Tamayo, The Molecular Signatures Database (MSigDB) hallmark gene set collection. *Cell Syst.* **1**, 417–425 (2015). doi:10.1016/j.cels.2015.12.004 Medline

72. E. J. Mendoza, K. Manguiat, H. Wood, M. Drebot, Two detailed plaque assay protocols for the quantification of infectious SARS-CoV-2. *Curr. Protoc. Microbiol.* **57**, ecpmc105 (2020). doi:10.1002/cpmc.105 Medline

73. A. C. G. Salina, D. Dos-Santos, T. S. Rodrigues, M. Fortes-Rocha, E. G. Freitas-Filho, D. L. Alzamora-Terrel, I. M. S. Castro, T. F. C. Fraga da Silva, M. H. F. de Lima, D. C. Nascimento, C. M. Silva, J. E. Toller-Kawahisa, A. Becerra, S. Oliveira, D. B. Caetité, L. Almeida, A. Y. Ishimoto, T. M. Lima, R. B. Martins, F. Veras, N. B. do Amaral, M. C. Giannini, L. P. Bonjorno, M. I. F. Lopes, M. N. Benatti, S. S. Batah, R. C. Santana, F. C. Vilar, M. A. Martins, R. L. Assad, S. C. L. de Almeida, F. R. de Oliveira, E. Arruda Neto, T. M. Cunha, J. C. Alves-Filho, V. L. D. Bonato, F. Q. Cunha, A. T. Fabro, H. I. Nakaya, D. S. Zamboni, P. Louzada-Junior, R. D. R. Oliveira, L. D. Cunha, Efferocytosis of SARS-CoV-2-infected dying cells impairs macrophage anti-inflammatory functions and clearance of apoptotic cells. *eLife* **11**, e74443 (2022). doi:10.7554/eLife.74443 Medline

74. S. Jin, C. F. Guerrero-Juarez, L. Zhang, I. Chang, R. Ramos, C.-H. Kuan, P. Myung, M. V. Plikus, Q. Nie, Inference and analysis of cell-cell communication using CellChat. *Nat. Commun.* **12**, 1088 (2021). doi:10.1038/s41467-021-21246-9 Medline

75. A. Chakrabarti, S. Banerjee, L. Franchi, Y.-M. Loo, M. Gale Jr., G. Núñez, R. H. Silverman, RNase L activates the NLRP3 inflammasome during viral infections. *Cell Host Microbe* **17**, 466–477 (2015). doi:10.1016/j.chom.2015.02.010 Medline

76. N. Magusali, A. C. Graham, T. M. Piers, P. Panichnantakul, U. Yaman, M. Shoai, R. H. Reynolds, J. A. Botia, K. J. Brookes, T. Guetta-Baranes, E. Bellou, S. Bayram, D. Sokolova, M. Ryten, C. Sala Frigerio, V. Escott-Price, K. Morgan, J. M. Pocock, J. Hardy, D. A. Salih, A genetic link between risk for Alzheimer's disease and severe COVID-19 outcomes via the *OAS1* gene. *Brain* **144**, 3727–3741 (2021). doi:10.1093/brain/awab337 Medline

77. S. Zhou, G. Butler-Laporte, T. Nakanishi, D. R. Morrison, J. Afilalo, M. Afilalo, L. Laurent, M. Pietzner, N. Kerrison, K. Zhao, E. Brunet-Ratnasingham, D. Henry, N. Kimchi, Z. Afrasiabi, N. Rezk, M. Bouab, L. Petitjean, C. Guzman, X. Xue, C. Tselios, B.

Vulesevic, O. Adeleye, T. Abdullah, N. Almamlouk, Y. Chen, M. Chassé, M. Durand, C. Paterson, J. Normark, R. Frithiof, M. Lipcsey, M. Hultström, C. M. T. Greenwood, H. Zeberg, C. Langenberg, E. Thysell, M. Pollak, V. Mooser, V. Forgetta, D. E. Kaufmann, J. B. Richards, A Neanderthal OAS1 isoform protects individuals of European ancestry against COVID-19 susceptibility and severity. *Nat. Med.* **27**, 659–667 (2021). doi:10.1038/s41591-021-01281-1 Medline

78. H. Zeberg, S. Pääbo, A genomic region associated with protection against severe COVID-19 is inherited from Neandertals. *Proc. Natl. Acad. Sci. U.S.A.* **118**, e2026309118 (2021). doi:10.1073/pnas.2026309118 Medline

79. J. E. Huffman, G. Butler-Laporte, A. Khan, E. Pairo-Castineira, T. G. Drivas, G. M. Peloso, T. Nakanishi, A. Ganna, A. Verma, J. K. Baillie, K. Kiryluk, J. B. Richards, H. Zeberg; COVID-19 Host Genetics Initiative, Multi-ancestry fine mapping implicates *OAS1* splicing in risk of severe COVID-19. *Nat. Genet.* **54**, 125–127 (2022). doi:10.1038/s41588-021-00996-8 Medline

80. K. Cho, M. Yamada, K. Agematsu, H. Kanegane, N. Miyake, M. Ueki, T. Akimoto, N. Kobayashi, S. Ikemoto, M. Tanino, A. Fujita, I. Hayasaka, S. Miyamoto, M. Tanaka-Kubota, K. Nakata, M. Shiina, K. Ogata, H. Minakami, N. Matsumoto, T. Ariga, Heterozygous mutations in *OAS1* cause infantile-onset pulmonary alveolar proteinosis with hypogammaglobulinemia. *Am. J. Hum. Genet.* **102**, 480–486 (2018). doi:10.1016/j.ajhg.2018.01.019 Medline

81. T. Magg, T. Okano, L. M. Koenig, D. F. R. Boehmer, S. L. Schwartz, K. Inoue, J. Heimall, F. Licciardi, J. Ley-Zaporozhan, R. M. Ferdman, A. Caballero-Oteyza, E. N. Park, B. M. Calderon, D. Dey, H. Kanegane, K. Cho, D. Montin, K. Reiter, M. Griese, M. H. Albert, M. Rohlfs, P. Gray, C. Walz, G. L. Conn, K. E. Sullivan, C. Klein, T. Morio, F. Hauck, Heterozygous *OAS1* gain-of-function variants cause an autoinflammatory immunodeficiency. *Sci. Immunol.* **6**, eabf9564 (2021). doi:10.1126/sciimmunol.abf9564 Medline

82. A. G. Hovanessian, R. E. Brown, I. M. Kerr, Synthesis of low molecular weight inhibitor of protein synthesis with enzyme from interferon-treated cells. *Nature* **268**, 537–540 (1977). doi:10.1038/268537a0 Medline

83. I. M. Kerr, R. E. Brown, pppA2′p5′A2′p5′A: an inhibitor of protein synthesis synthesized with an enzyme fraction from interferon-treated cells. *Proc. Natl. Acad. Sci. U.S.A.* **75**, 256–260 (1978). doi:10.1073/pnas.75.1.256 Medline

84. M. Drappier, T. Michiels, Inhibition of the OAS/RNase L pathway by viruses. *Curr. Opin. Virol.* **15**, 19–26 (2015). doi:10.1016/j.coviro.2015.07.002 Medline

85. R. H. Silverman, Viral encounters with 2′,5′-oligoadenylate synthetase and RNase L during the interferon antiviral response. *J. Virol.* **81**, 12720–12729 (2007). doi:10.1128/JVI.01471-07 Medline

86. R. J. Lin, H.-P. Yu, B.-L. Chang, W.-C. Tang, C.-L. Liao, Y.-L. Lin, Distinct antiviral roles for human 2′,5′-oligoadenylate synthetase family members against dengue virus infection. *J. Immunol.* **183**, 8035–8043 (2009). doi:10.4049/jimmunol.0902728 Medline

87. Y. C. Kwon, J. I. Kang, S. B. Hwang, B. Y. Ahn, The ribonuclease L-dependent antiviral roles of human 2′,5′-oligoadenylate synthetase family members against hepatitis C virus. *FEBS Lett.* **587**, 156–164 (2013). doi:10.1016/j.febslet.2012.11.010 Medline

88. Y. Li, S. Banerjee, Y. Wang, S. A. Goldstein, B. Dong, C. Gaughan, R. H. Silverman, S. R. Weiss, Activation of RNase L is dependent on OAS3 expression during infection with diverse human viruses. *Proc. Natl. Acad. Sci. U.S.A.* **113**, 2241–2246 (2016). doi:10.1073/pnas.1519657113 Medline

89. J. N. Whelan, Y. Li, R. H. Silverman, S. R. Weiss, Zika virus production is resistant to RNase L antiviral activity. *J. Virol.* **93**, e00313-19 (2019). doi:10.1128/JVI.00313-19 Medline

90. C. S. Thakur, Z. Xu, Z. Wang, Z. Novince, R. H. Silverman, A convenient and sensitive fluorescence resonance energy transfer assay for RNase L and 2′,5′ oligoadenylates. *Methods Mol. Med.* **116**, 103–113 (2005). doi:10.1385/1-59259-939-7:103 Medline

91. K. Sacco, R. Castagnoli, S. Vakkilainen, C. Liu, O. M. Delmonte, C. Oguz, I. M. Kaplan, S. Alehashemi, P. D. Burbelo, F. Bhuyan, A. A. de Jesus, K. Dobbs, L. B. Rosen, A. Cheng, E. Shaw, M. S. Vakkilainen, F. Pala, J. Lack, Y. Zhang, D. L. Fink, V. Oikonomou, A. L. Snow, C. L. Dalgard, J. Chen, B. A. Sellers, G. A. Montealegre Sanchez, K. Barron, E. Rey, C. Vial, M. C. Poli, A. Licari, D. Montagna, G. L. Marseglia, F. Licciardi, U. Ramenghi, V. Discepolo, A. L. Vecchio, A. Guarino, E. M. Eisenstein, L. Imberti, A. Sottini, A. Biondi, S. Mató, D. Gertsbacher, M. Truong, M. A. Stack, M. Magliocco, M. Bosticardo, T. Kawai, J. J. Danielson, T. Hulett, M. Askenazi, J. I. Cohen, H. C. Su, D. B. Kuhns, M. S. Lionakis, T. M. Snyder, S. M. Holland, R. Goldbach-Mansky, J. S. Tsang, L. D. Notarangelo; NIH, NIAID Immune Response to COVID Group; Chile MIS-C Group; Pavia Pediatric COVID-19 Group, Multiomics approach identifies novel age-, time- and treatment-related immunopathological signatures in MIS-C and pediatric COVID-19, version 1.0.0, Zenodo (2021); https://doi.org/10.5281/zenodo.5524378.

92. M. A. DePristo, E. Banks, R. Poplin, K. V. Garimella, J. R. Maguire, C. Hartl, A. A. Philippakis, G. del Angel, M. A. Rivas, M. Hanna, A. McKenna, T. J. Fennell, A. M. Kernytsky, A. Y. Sivachenko, K. Cibulskis, S. B. Gabriel, D. Altshuler, M. J. Daly, A framework for variation discovery and genotyping using next-generation DNA sequencing data. *Nat. Genet.* **43**, 491–498 (2011). doi:10.1038/ng.806 Medline

93. H. Li, R. Durbin, Fast and accurate short read alignment with Burrows–Wheeler transform. *Bioinformatics* **25**, 1754–1760 (2009). doi:10.1093/bioinformatics/btp324 Medline

94. W. McLaren, L. Gil, S. E. Hunt, H. S. Riat, G. R. S. Ritchie, A. Thormann, P. Flicek, F. Cunningham, The Ensembl Variant Effect Predictor. *Genome Biol.* **17**, 122 (2016). doi:10.1186/s13059-016-0974-4 Medline

95. N. Hernandez, I. Melki, H. Jing, T. Habib, S. S. Y. Huang, J. Danielson, T. Kula, S. Drutman, S. Belkaya, V. Rattina, L. Lorenzo-Diaz, A. Boulai, Y. Rose, N. Kitabayashi, M. P. Rodero, C. Dumaine, S. Blanche, M.-N. Lebras, M. C. Leung, L. S. Mathew, B. Boisson, S.-Y. Zhang, S. Boisson-Dupuis, S. Giliani, D. Chaussabel, L. D. Notarangelo, S. J. Elledge, M. J. Ciancanelli, L. Abel, Q. Zhang, N. Marr, Y. J. Crow, H. C. Su, J.-L. Casanova, Life-threatening influenza pneumonitis in a child with inherited IRF9 deficiency. *J. Exp. Med.* **215**, 2567–2585 (2018). doi:10.1084/jem.20180628 Medline

96. I. Korsunsky, N. Millard, J. Fan, K. Slowikowski, F. Zhang, K. Wei, Y. Baglaenko, M. Brenner, P. R. Loh, S. Raychaudhuri, Fast, sensitive and accurate integration of single-cell data with Harmony. *Nat. Methods* **16**, 1289–1296 (2019). doi:10.1038/s41592-019-0619-0 Medline

97. D. Aran, A. P. Looney, L. Liu, E. Wu, V. Fong, A. Hsu, S. Chak, R. P. Naikawadi, P. J. Wolters, A. R. Abate, A. J. Butte, M. Bhattacharya, Reference-based analysis of lung single-cell sequencing reveals a transitional profibrotic macrophage. *Nat. Immunol.* **20**, 163–172 (2019). doi:10.1038/s41590-018-0276-y Medline

98. G. Monaco, B. Lee, W. Xu, S. Mustafah, Y. Y. Hwang, C. Carré, N. Burdin, L. Visan, M. Ceccarelli, M. Poidinger, A. Zippelius, J. Pedro de Magalhães, A. Larbi, RNA-seq signatures normalized by mRNA abundance allow absolute deconvolution of human immune cell types. *Cell Rep.* **26**, 1627–1640.e7 (2019). doi:10.1016/j.celrep.2019.01.041 Medline

99. M. I. Love, W. Huber, S. Anders, Moderated estimation of fold change and dispersion for RNA-seq data with DESeq2. *Genome Biol.* **15**, 550 (2014). doi:10.1186/s13059-014-0550-8 Medline

100. R. C. Team, R: A Language and Environment for Statistical Computing (R Foundation for Statistical Computing, 2018); https://www.R-project.org/.
