# Supplementary material for: Inborn errors of OAS–RNase L in SARS-CoV-2–related multisystem inflammatory syndrome in children
Source: Science. 2023 Feb 10;379(6632):eabo3627. doi: 10.1126/science.abo3627 (PMC10451000; doi:10.1126/science.abo3627)
Supplement: Supplementary file 4 — Data S1 and S2 [file science.abo3627_data_s1_and_s2.zip › science.abo3627_data_captions.pdf]

Data S1. SARS-CoV-2–induced immune responses across all PBMC cell types

Data S2. OAS–RNase L-deficient myeloid cells display enhanced pro-inflammatory responses to SARS-CoV-2
